# Temporal Gene Expression Kinetics for Human Keratinocytes Exposed to Hyperthermic Stress

**DOI:** 10.3390/cells2020224

**Published:** 2013-04-10

**Authors:** Ibtissam Echchgadda, Caleb C. Roth, Cesario Z. Cerna, Gerald J. Wilmink

**Affiliations:** 1 Air Force Research Laboratory, 711th Human Performance Wing, Human Effectiveness Directorate, Bioeffects Division, Radio Frequency Bioeffects Branch, 4141 Petroleum Road, Bldg. 3260, Fort Sam Houston, TX 78234, USA; E-Mail: ibtissam.echchgadda.ctr@mail.mil; 2 National Academy of Sciences NRC Research Associateship, Fort Sam Houston, TX 78234, USA; 3 Department of Radiology, University of Texas Health Science Center San Antonio, San Antonio, TX 78229, USA; E-Mail: rothc@livemail.uthscsa.edu; 4 General Dynamics Information Technology, Fort Sam Houston, TX 78234, USA; E-Mail: cesario.z.cerna.ctr@mail.mil

**Keywords:** keratinocytes, heat shock, gene expression, cellular stress response, bioinformatics

## Abstract

The gene expression kinetics for human cells exposed to hyperthermic stress are not well characterized. In this study, we identified and characterized the genes that are differentially expressed in human epidermal keratinocyte (HEK) cells exposed to hyperthermic stress. In order to obtain temporal gene expression kinetics, we exposed HEK cells to a heat stress protocol (44 °C for 40 min) and used messenger RNA (mRNA) microarrays at 0 h, 4 h and 24 h post-exposure. Bioinformatics software was employed to characterize the chief biological processes and canonical pathways associated with these heat stress genes. The data shows that the genes encoding for heat shock proteins (HSPs) that function to prevent further protein denaturation and aggregation, such as HSP40, HSP70 and HSP105, exhibit maximal expression immediately after exposure to hyperthermic stress. In contrast, the smaller HSPs, such as HSP10 and HSP27, which function in mitochondrial protein biogenesis and cellular adaptation, exhibit maximal expression during the “recovery phase”, roughly 24 h post-exposure. These data suggest that the temporal expression kinetics for each particular HSP appears to correlate with the cellular function that is required at each time point. In summary, these data provide additional insight regarding the expression kinetics of genes that are triggered in HEK cells exposed to hyperthermic stress.

## 1. Introduction

Human beings frequently encounter a wide variety of hyperthermic stressors. Common examples include exposure to hot conducting objects, elevated environmental temperatures, as well as electromagnetic radiation from the sun and numerous man-made sources, such as lasers, communication devices and imaging systems [1,2,3,4,5,6,7,8,9,10]. The heat generated by hyperthermic stressors can be harmful to organisms, because it can temporarily or permanently alter the conformation and function of vital intracellular biomacromolecules (*i.e.*, lipids, proteins and nucleic acids). Such modifications can result in an array of damaging effects, including cellular morphological alterations (*i.e.*, shape, size, irregularities and roughness), cytoskeleton restructuring, organelle reorganization and/or fragmentation and direct interference with intracellular transport processes [11].

Hyperthermic effects can interfere with an organism’s normal homeostasis, and thus, if they are not adequately counteracted, they can threaten its survival. In order to survive and minimize the effects of hyperthermic stress, virtually all cells in living organisms have evolved sophisticated sensing, response and adaptation mechanisms. The crux of these mechanisms is the molecular defense reaction, most commonly referred to as the cellular stress response (CSR). The CSR is immediately activated in response to stress stimuli, and a hallmark of this response is the robust transcriptional expression of hundreds of diverse genes. These genes encode for a multitude of proteins involved in many important functions, including cellular survival, deoxyribonucleic acid (DNA) sensing and repair, redox regulation, proteolysis, molecular chaperoning, cell cycle control and apoptosis [12,13]. Furthermore, each CSR gene exhibits unique expression profiles, which differ in magnitude, onset time and duration of expression [4,10]. 

The epidermis, the outermost layer of an organism’s skin, regularly encounters a wide variety of stressors. In fact, the primary function of the epidermis is to give organisms a physiological protection barrier against environmental stimuli, such as hyperthermic stressors. This protective role is mediated primarily by keratinocytes, the predominant epithelial cell type (~95%). Due to their anatomical location, keratinocytes are habitually exposed to environmental temperature variations, and thus, they are the primary and frequent target of hyperthermic stress. In addition, their frequent exposure to heat stress has also been reported to render them with specialized cellular responses, which make them more resistant to stress than virtually all other cell types [14,15]. Clearly, keratinocytes carry a disproportionate burden in maintaining homeostasis for organisms; thus, they are vital players in sensing, responding and adapting to the burdens of hyperthermic stress.

Over the past decade, numerous microarray experiments have been performed to better understand the stress response of human epidermal keratinocytes (HEK) [15,16,17,18,19]. These studies have been particularly fruitful, because they have resulted in the identification of hundreds of stress-inducible genes. However, previous gene expression studies have failed to provide a comprehensive picture of the cellular stress response for several reasons. First, the microarray gene chips utilized in most of the previous studies were incomplete and, thus, provided expression kinetics for only a fraction of the genes in the human genome. Second, many previous studies made gene expression measurements at a single time point; as a result, temporal gene expression kinetics could not be ascertained. Finally, the majority of past studies examined the response of skin cells to ultraviolet stress; however, few studies have specifically analyzed the transcriptional response of HEK to hyperthermic stress [10].

**Figure 1:**
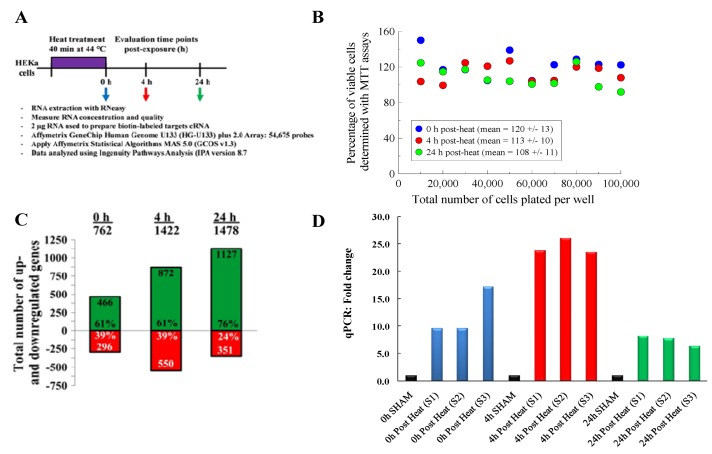
(**A**) A schematic of the heat shock exposure protocol and post analysis techniques. (**B**) Cellular MTT viability for keratinocytes exposed to hyperthermic stress. Viability percentage data (relative to untreated samples) was measured for wells with concentration densities ranging from 10,000 to 100,000 cells per well. Viability was measured at 0, 4 and 24 h post-heat treatment. (**C**) Microarray global gene expression profiles. The data shows that 762, 1,422 and 1,478 total genes were differentially expressed at 0, 4 and 24 h post-exposure, respectively. (**D**) Gene expression for minimal stress protein, heat shock protein 70 (HSPA6) using qRT-PCR. The mRNA expression fold values were measured for sham and heat treatment groups. For each time point, three samples (S1, S2 and S3) were assessed, each in triplicates. Values were calculated in relation to β-actin and normalized to a separate RNA calibrator.

In this study, we sought to identify and characterize the genes that are differentially expressed in HEK exposed to hyperthermic stress. To obtain temporal gene expression kinetics, we exposed HEK to a heat stress protocol (44 °C for 40 min) and used mRNA microarrays at three time points: 0 h, 4 h and 24 h post-heat treatment, as outlined in Figure 1A. Then, we used bioinformatics software to characterize the chief biological processes and canonical pathways associated with these genes. These data provide valuable new insights that give us a much clearer picture of the genes and intracellular signaling pathways that are triggered in human cells exposed to hyperthermic stress.

## 2. Results and Discussion

### 2.1. Keratinocytes Induce an Appreciable Stress Response When Exposed To Hyperthermic Stress

We conducted an initial set of experiments to investigate the impact that our selected hyperthermia stress protocol had on cellular viability. For these studies, HEKs were exposed to hyperthermic stress and cellular metabolic activity was measured at 0, 4 and 24 h post-exposure using MTT assays (Figure 1B). For all cell densities tested, the data shows that greater than 90% of the cells survive after being exposed to our hyperthermic stress protocol. This data confirms that the HEKs cells were viable post-heat exposure.

After confirming that the HEK cells survived post-exposure, we then sought to test whether our selected hyperthermic stress protocol induced an appreciable transcriptional stress response To investigate the transcriptional stress response, we exposed HEK cells to hyperthermic stress and used microarray gene chips to quantify the expression level of genes at 0, 4 and 24 h post-exposure (Figure 1A). Since a signature feature of an appreciable stress response is the rapid and marked upregulation of minimal stress genes [12], we conducted microarray and PCR analyses to examine the genes that HEKs express when exposed to hyperthermia (Figure 1C,D). Using Ingenuity Pathway Analysis (IPA) software, we filtered the microarray data using the following cut-offs: (i) an absolute value of expression magnitude (log_2_ ratio relative to control) greater or equal to 1.5; and (ii) a *p*-value less than or equal to 0.05. Of the 54,675 probe sets tested on each gene chip, we found that 762, 1,422 and 1,478 genes were differentially expressed at the 0, 4 and 24 h time points, respectively (Figure 1C). The number of upregulated genes increased in a logarithmic fashion from 466 at 0 h, 872 at 4 h and up to 1,127 at 24 h. Interestingly, roughly 60% of the transcripts regulated at 0 and 4 h were upregulated, but by 24 h, ~75% of the differentially expressed genes were upregulated.

Specifically, we found that the heat-treated groups expressed transcripts for many heat shock proteins (Hsps). We also found that the transcripts encoding for heat shock protein 70 (HSPA6) exhibited the greatest increase in expression and the highest level of statistical significance. To validate these microarray results, we then conducted qRT-PCR analyses for HSPA6 (Hsp70) (Figure 1D). For all of the samples tested, we found that the heat-treated treatment group exhibited statistically significant increases in HSPA6 expression, ranging between 10- and 25-fold compared to the sham group. These results were consistent with the microarray data, which showed that HSPA6 expression increased between 4- and 32-fold.

### 2.2. Temporal Gene Expression Profiles for Keratinocytes Exposed To Hyperthermic Stress

**Figure 2:**
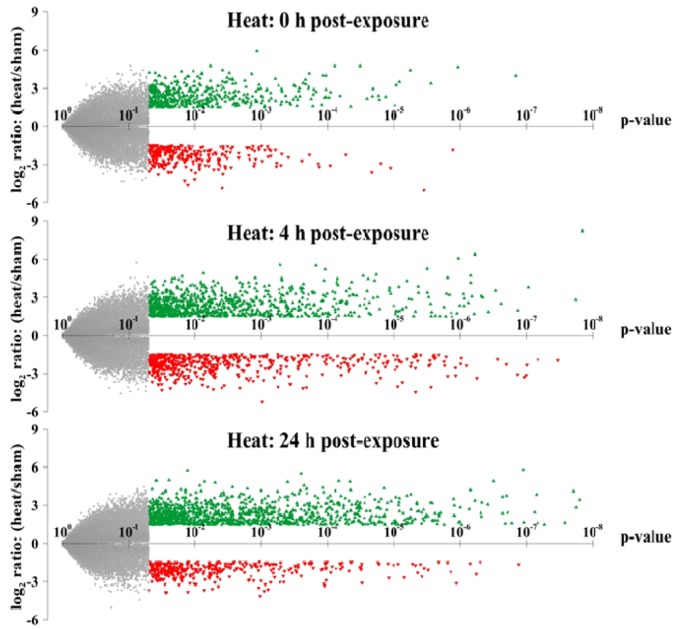
Volcano plots of the gene expression profiles at each time point. The magnitude of differential expression (log_2_ fold-change) is plotted *versus* the level of statistical significance (*p*-value) of all genes in the microarray for 0, 4 and 24 h post-heat exposure. Upregulated and downregulated genes with absolute log_2_ ratios ≥1.5 and *p*-values ≤0.05 are depicted with green and red triangle symbols, respectively. Insignificant genes are denoted with light gray symbols.

In order to visualize the global distribution of the gene expression profiles, we then created volcano plots for all genes at each time point. In these plots, the level of statistical significance (*p*-value) for each gene is plotted *versus* the fold-change in expression level relative to untreated sham (Figure 2). Genes with the highest level of statistical significance appear on the right side of the plot. In addition, upregulated genes that display large-magnitude fold-changes appear towards the top of the plot (denoted with green triangles), whereas downregulated genes appear towards the bottom (denoted with red triangles). We found that considerably more genes were differentially expressed at 4 h and 24 h than at the 0 h time point. We also found that the transcripts expressed at the later time points also exhibited lower *p*-values and larger-magnitude changes in expression level than the genes at 0 h. Collectively, our results show that hyperthermic stress triggers a rapid, robust and extensive transcriptional response in HEK cells. The temporal kinetics expression data also show that this stress response is immediately activated in response to stress and is sustained for many hours after exposure.

### 2.3. Biological Functions of Heat-Shock-Induced Genes

**Figure 3:**
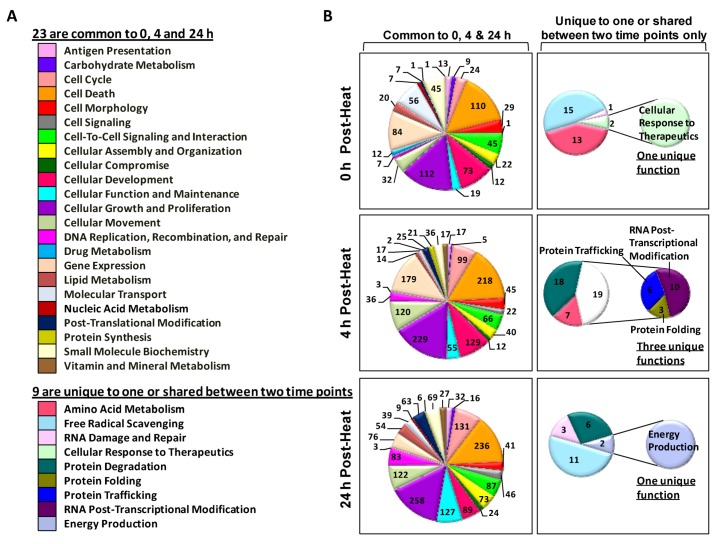
Diagrams of cellular and molecular functions associated with genes expressed in human epidermal keratinocyte (HEK) cells exposed to heat shock. Following an Ingenuity Pathway Analysis (IPA) core analysis, the molecular and cellular functions linked to each recovery period were sorted and illustrated. (**A**) A list of the 23 biological functions common to all three time points and nine functions unique to one time point or shared between two time points; (**B**) Pie charts of gene functions at 0, 4 and 24 h post-exposure. The pie chart sectors represent the number of genes associated with each function.

After identifying the genes expressed in response to hyperthermic stress, we then used IPA software to identify each gene’s primary biological functions. Figure 3 contains pie charts of the total number of genes associated with each cellular and molecular function. We found that 23 biological functions were common to all three time points. Interestingly, only nine functions in this group were either unique to one or shared by two time points (Figure 3). In general, the 0 h and 24 h time points share many genes with functions required for free radical scavenging, whereas only the 4 h time point contains genes with functions dedicated primarily to protein regulation (*i.e.*, protein trafficking and protein folding) (Figure 3B).

**Figure 4:**
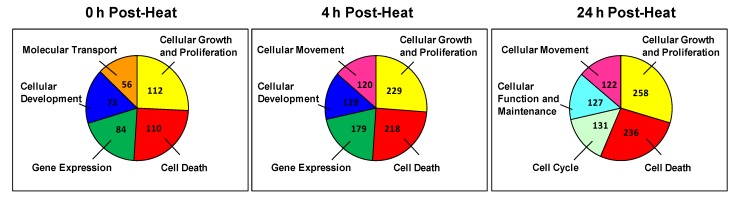
Pie chart of the top five cellular and molecular functions at each time point. Each sector of the pie chart represents the total number of genes expressed for each cellular function. Genes were filtered for an absolute value log_2_ ratio ≥1.5 and a significance value of *p* ≤ 0.05.

Next, we identified the biological function groups that had the greatest number of differentially expressed genes. For each time point, we generated color-coded pie charts containing the top five biological functions and the number of genes in each group (Figure 4). We found that two biological functions (*i.e.*, cellular growth and proliferation and cell death) appeared in the top five at each time point. In addition to these functions, the 0 h and 4 h time points also shared two more top functions, which are associated with cellular development and gene expression. The only major difference between these time points was that the 0 h time point had more genes associated with molecular transport, whereas the 4 h group had more genes involved in cellular movement functions. In contrast, despite exhibiting a similar number of regulated genes, the 4 h and 24 h groups only shared three top functions: cellular movement, cell death and cell growth. The primary difference between these time points is that the 4 h group had more genes dedicated to cellular development and gene expression, whereas the 24 h group exhibited genes with cellular function and cell cycle processes.

### 2.4. Identification of Shared and Unique Genes

**Figure 5:**
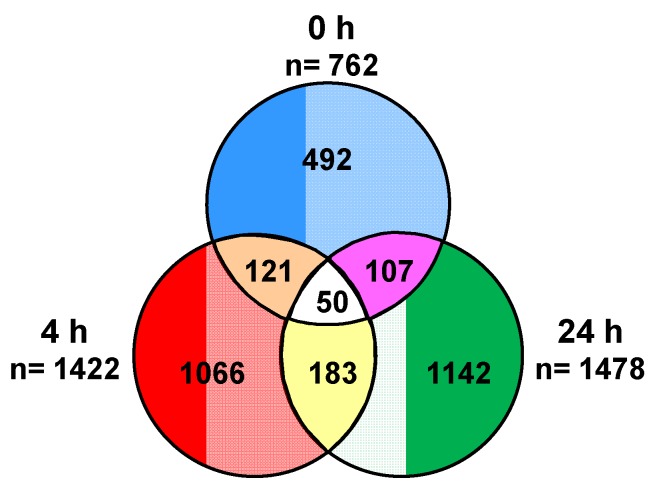
Identification of unique and shared genes. The Venn diagram indicates the number of unique and common differentially expressed genes at each time point. Biomarker comparison analysis was performed in IPA.

We next sought to determine whether any of the differentially expressed genes were observed at all time points. To answer this question, we performed a biomarker filter analysis. This tool allowed us to identify the genes that were unique and shared by each group. The Venn diagram in Figure 5 shows the total number of genes that were shared and unique to each time point. A total of 492, 1,066 and 1,142 genes were unique to the 0 h, 4 h and 24 h evaluation groups, respectively. We found that 121 genes were common to both the 0 h and 4 h, 107 genes to the 0 h and 24 h and 183 genes to the 4 h and 24 h. Notably, only 50 genes were shared among all three recovery time points. A comprehensive list of these genes is provided as supplementary material (Supplementary Tables 1–6).

**Figure 6:**
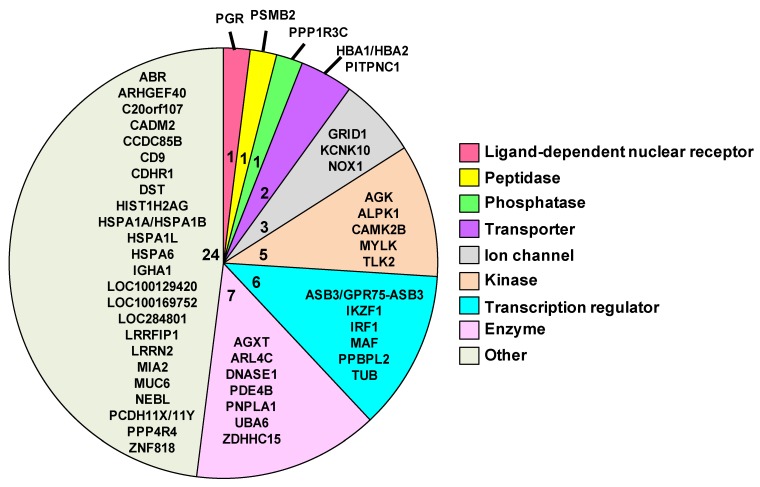
Pie chart of 50 shared biomarkers. Each sector of the pie represents the number of genes associated with each function. The symbols and number of genes are also provided for each function. Genes were filtered for an absolute value log_2_ ratio ≥1.5 and a significance value of *p* ≤ 0.05.

After identifying the 50 genes that were expressed at each time point, we then performed a series of analyses to better understand the function of each gene. For these analyses, we used the following web-based resources: IPA, GeneCards Human Gene Database, HGNC and Gene Ontology (GO). The pie chart in Figure 6 contains the symbols for each of the 50 common genes. This pie chart is also further divided by the family of each gene and the number of genes per family, which is denoted by the number towards the center of each sector of the pie chart. The gene families are color coded and are specified in the legend. The following eight families are provided in the figure: ligand-dependent nuclear receptor, peptidase, phosphatase, transporter, ion channel, kinase, transcription regulator, enzymes and others. The twenty-four genes included in the “other” group consist of genes that were not classified by IPA.

**Figure 7:**
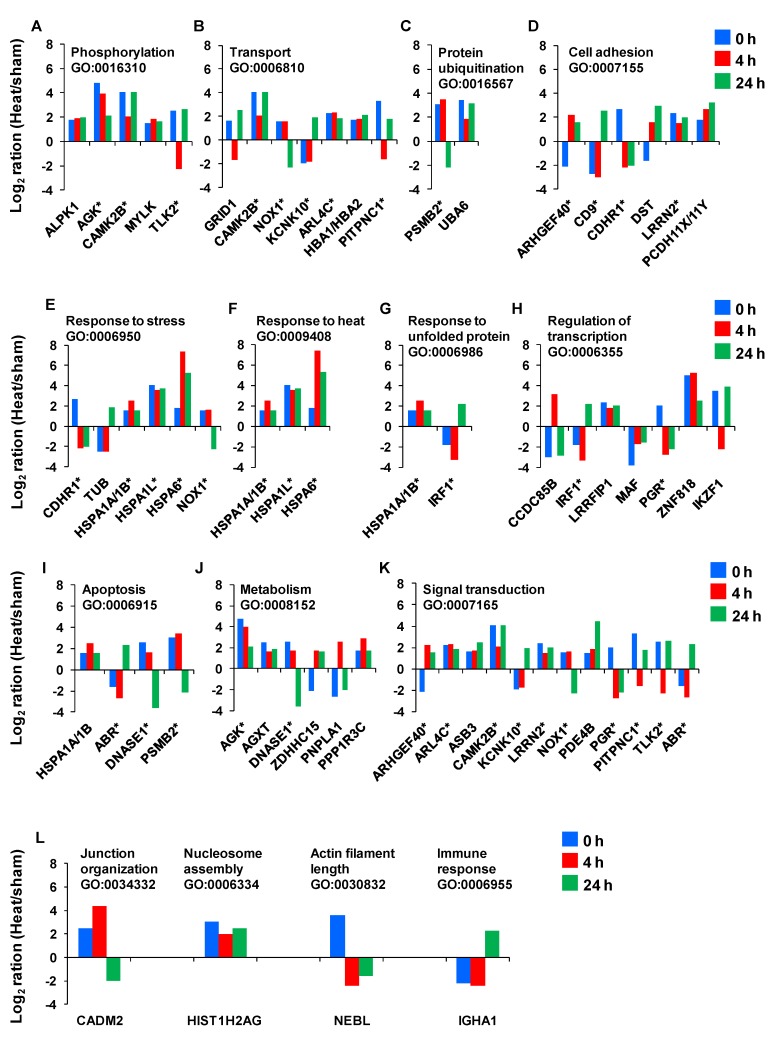
The temporal expression kinetics for the genes with known gene ontology (GO). Asterisks indicate the genes with more than one biological process.

Next, we sought to examine the temporal expression kinetics for the genes with known biological functions. We found that only 44 of the 50 genes identified had well-characterized biological processes and GO annotations. Figure 7 provides the magnitude of the fold-change in expression for each of these genes as a function of time. The asterisks denote genes that have multiple key biological functions. The genes include the chaperones that function in response to stress and protein unfolding. These genes encode for the following heat shock 70 kDa proteins: HSPA6, HSPA1L and HSPA1A/A1B.

Additional genes responsible for the response to stress or heat are CDHR1 (cadherin-related family member 1), TUB (tubby protein homolog), NOX1 (NADPH oxidase 1) and IRF1 (interferon regulatory factor 1). Two genes—PSMB2 (proteasome subunit beta type 2) and UBA6 (ubiquitin-like modifier activating enzyme 6)—play roles in protein ubiquitination. CDHR1, ARHGEF40 (Rho guanine nucleotide exchange factor (GEF) 40), CD9 (CD9 molecule), DST (dystonin), LRRN2 (leucine rich repeat neuronal 2) and PCDH11X/Y (protocadherin 11 Y-linked) support cell adhesion. ALPK1 (alpha-kinase 1), AGK (acylglycerol kinase), CAMK2B (calcium/calmodulin-dependent protein kinase II beta), MYLK (myosin light chain kinase) and TLK2 (tousled-like kinase 2) assist in phosphorylation, whereas, GRID1 (glutamate receptor delta-1 subunit), KCNK10 (potassium channel subfamily K member 10), ARL4C (ADP-ribosylation factor-like 4), HBA1/HBA2 (hemoglobin alpha 1), PITPNC1 (cytoplasmic phosphatidylinositol transfer protein 1), CAMK2B and NOX1 support the transport function. In addition, CADM2 (cell adhesion molecule 2), HIST1H2AG (histone cluster 1, H2ag), NEBL (nebulette) and IGHA1 play roles in junction organization, nucleosome assembly, actin filament length and immune response, respectively. Furthermore, as indicated in Figure 7, some of the above mentioned genes and the remaining ones are involved in processes related to homeostasis response. These include regulation of transcription, apoptosis, metabolism and signal transduction related-genes.

**Figure 8:**
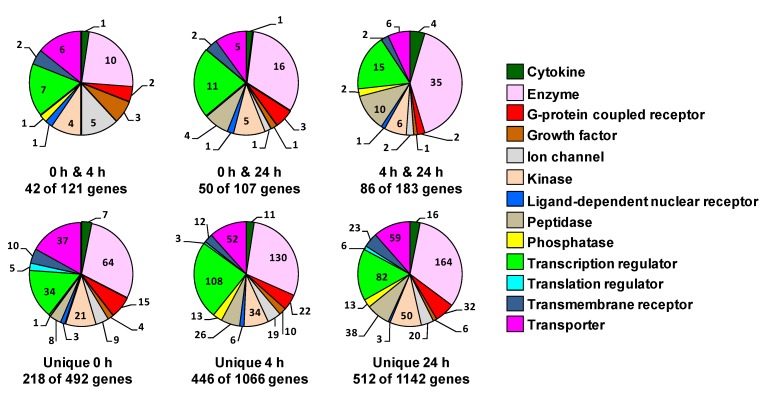
Gene family of the genes that are unique or are shared at two time points. The pie chart sectors represent the number of genes associated with each function indicated in the legend. The numbers of these genes are indicated for each function. The genes classified by IPA as “other” were not included in this representation. Genes were filtered for an absolute value log_2_ ratio ≥1.5 and a significance value of *p* ≤ 0.05.

The gene families related to the unique genes or the genes shared between each two time points, as well as the number of genes per each family are illustrated in the pie charts in Figure 8. The gene families included those mentioned for the 50 common genes in addition to other gene families, such as G-protein coupled receptor, growth factor, cytokine, translation regulator and transmembrane receptor family. The genes classified by IPA as “other” were omitted in this representation. A list of all these genes and fold-change following heat-shock in the different interval of recovery is added as tables in the supplemental materials.

**Figure 9:**
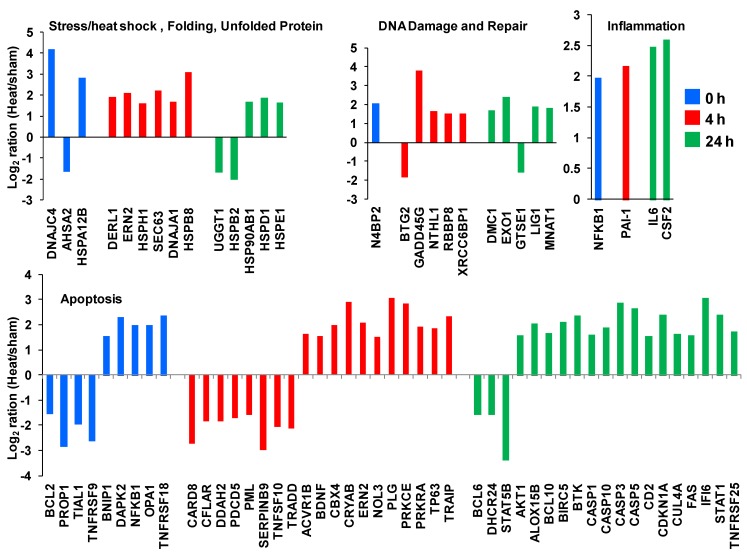
Gene expression profiles for a set of unique genes involved in several well-characterized pathways of the cellular stress response.

In Figure 9, we highlight a set of genes from the unique genes list that are recognized to play roles in cellular stress response. These include genes involved as heat shock proteins and genes supporting DNA damage and repair, inflammation and apoptosis.

### 2.5. Pathway Analysis of Microarray Analyzed Data

We next used IPA to analyze the metabolic and signaling pathways that are affected in response to heat stress following the three post-heat time points. We found that at 0 h, 33 of 247 canonical pathways were expressed with statistical significance, defined as [(−log(*p*-value) ≥ 1.3 or *p*-value ≤ 0.05] (Table 1). At 4 h post-heat, 19 out of 198 scored Ingenuity Canonical Pathways had significant likelihood (Table 2). At 24 h post-heat, IPA scored 229 Ingenuity Canonical Pathways, of which 36 had significant *p*-values (Table 3).

**Table 1:** Significant canonical pathways at 0 h post-heat exposure. Two-hundred seventy-four Ingenuity Canonical Pathways were scored at 0 h post-heat exposure; only the 33 listed pathways fit the cut-off of *p*-value ≤ 0.05.

Ingenuity Canonical Pathways	−log(*p*-value)	Ratio	# of Molecules
IL-12 Signaling and Production in Macrophages	4.66	0.118	16
Glucocorticoid Receptor Signaling	1.83	0.061	16
Role of Macrophages, Fibroblasts and Endothelial Cells in Rheumatoid Arthritis	1.41	0.054	16
Production of Nitric Oxide and Reactive Oxygen Species in Macrophages	1.85	0.070	13
Clathrin-Mediated Endocytosis Signaling	1.80	0.069	13
Dopamine-DARPP32 Feedback in cAMP Signaling	1.46	0.067	11
NF-κB Signaling	1.42	0.065	11
RAR Activation	1.35	0.063	11
Neuropathic Pain Signaling in Dorsal Horn Neurons	2.35	0.094	10
Type II Diabetes Mellitus Signaling	2.07	0.079	10
Virus Entry via Endocytic Pathways	2.28	0.098	9
Synaptic Long-Term Potentiation	1.88	0.087	9
fMLP Signaling in Neutrophils	1.78	0.077	9
PKCθ Signaling in T-lymphocytes	1.64	0.071	9
Role of Pattern Recognition Receptors in Recognition of Bacteria and Viruses	2.29	0.104	8
T-cell Receptor Signaling	1.61	0.078	8
iCOS-iCOSL Signaling in T Helper Cells	1.45	0.071	8
Rac Signaling	1.45	0.068	8
Pancreatic Adenocarcinoma Signaling	1.41	0.070	8
Renin-Angiotensin Signaling	1.33	0.070	8
Erythropoietin Signaling	1.95	0.095	7
Macropinocytosis Signaling	1.92	0.092	7
NF-κB Activation by Viruses	1.76	0.089	7
Ceramide Signaling	1.62	0.085	7
RANK Signaling in Osteoclasts	1.42	0.076	7
Calcium-induced T-lymphocyte Apoptosis	1.72	0.098	6
Growth Hormone Signaling	1.42	0.085	6
Complement System	2.24	0.152	5
MSP-RON Signaling Pathway	1.66	0.109	5
Cell Cycle: G1/S Checkpoint Regulation	1.33	0.085	5
Role of CHK Proteins in Cell Cycle Checkpoint Control	1.50	0.114	4
IL-9 Signaling	1.46	0.105	4
April Mediated Signaling	1.31	0.095	4

**Table 2:** Significant canonical pathways in 4 h post-heat exposure. 198 Ingenuity Canonical Pathways were scored at 4 h post-heat exposure; only the 19 listed pathways fit the cut-off of *p*-value ≤ 0.05.

Ingenuity Canonical Pathways	−log(*p*-value)	Ratio	# of Molecules
Leukocyte Extravasation Signaling	2.58	0.127	24
Huntington’s Disease Signaling	2.39	0.114	26
Tight Junction Signaling	2.34	0.127	20
Bladder Cancer Signaling	2.15	0.144	13
VDR/RXR Activation	2.12	0.152	12
ATM Signaling	2.04	0.167	9
Coagulation System	2.02	0.200	7
Colorectal Cancer Metastasis Signaling	1.90	0.107	26
Wnt/β-catenin Signaling	1.90	0.118	20
Cyclins and Cell Cycle Regulation	1.76	0.126	11
Role of Macrophages, Fibroblasts and Endothelial Cells in Rheumatoid Arthritis	1.70	0.096	30
Nur77 Signaling in T-lymphocytes	1.62	0.140	8
IL-17A Signaling in Gastric Cells	1.57	0.200	5
Cell Cycle: G1/S Checkpoint Regulation	1.49	0.136	8
IL-17A Signaling in Fibroblasts	1.49	0.154	6
Nitrogen Metabolism	1.49	0.171	6
Production of Nitric Oxide and Reactive Oxygen Species in Macrophages	1.37	0.103	19
Ceramide Signaling	1.35	0.122	10
Aldosterone Signaling in Epithelial Cells	1.35	0.106	17

**Table 3:** Significant canonical pathways at 24 h post-heat exposure. 229 Ingenuity Canonical Pathways were scored at 24 h post-heat exposure; only the 36 listed pathways fit the cut-off of *p*-value ≤ 0.05.

Ingenuity Canonical Pathways	−log(p-value)	Ratio	# of Molecules
Aryl Hydrocarbon Receptor Signaling	4.33	0.170	23
Linoleic Acid Metabolism	2.78	0.197	11
Role of IL-17A in Psoriasis	2.74	0.385	4
Bladder Cancer Signaling	2.72	0.167	14
Oncostatin M Signaling	2.52	0.235	7
TREM1 Signaling	2.39	0.189	9
Cell Cycle: G1/S Checkpoint Regulation	2.21	0.169	9
Fcγ Receptor-mediated Phagocytosis in Macrophages and Monocytes	2.08	0.149	13
Role of CHK Proteins in Cell Cycle Checkpoint Control	2.00	0.200	6
Glucocorticoid Receptor Signaling	1.94	0.108	29
Atherosclerosis Signaling	1.90	0.128	15
Cell Cycle: G2/M DNA Damage Checkpoint Regulation	1.88	0.167	7
Amino sugars Metabolism	1.88	0.159	10
Metabolism of Xenobiotics by Cytochrome P450	1.86	0.148	12
IL-8 Signaling	1.77	0.118	20
Airway Pathology in Chronic Obstructive Pulmonary Disease	1.75	0.375	2
LPS/IL-1 Mediated Inhibition of RXR Function	1.74	0.112	22
Chronic Myeloid Leukemia Signaling	1.71	0.127	12
Arachidonic Acid Metabolism	1.71	0.137	13
Sphingosine-1-phosphate Signaling	1.70	0.126	14
Hereditary Breast Cancer Signaling	1.67	0.125	14
Leukocyte Extravasation Signaling	1.66	0.116	21
Role of BRCA1 in DNA Damage Response	1.65	0.153	8
Pancreatic Adenocarcinoma Signaling	1.61	0.123	13
Role of IL-17F in Allergic Inflammatory Airway Diseases	1.56	0.167	6
IL-10 Signaling	1.53	0.139	9
IL-22 Signaling	1.52	0.208	4
Cellular Effects of Sildenafil (Viagra)	1.47	0.121	15
Inhibition of Angiogenesis by TSP1	1.47	0.182	5
Role of JAK Family Kinases in IL-6-type Cytokine Signaling	1.45	0.192	4
Tryptophan Metabolism	1.45	0.127	13
Purine Metabolism	1.41	0.106	27
Parkinson’s Signaling	1.39	0.222	3
p53 Signaling	1.35	0.126	11
cAMP-Mediated Signaling	1.33	0.107	22
IL-15 Production	1.32	0.172	4

**Figure 10:**
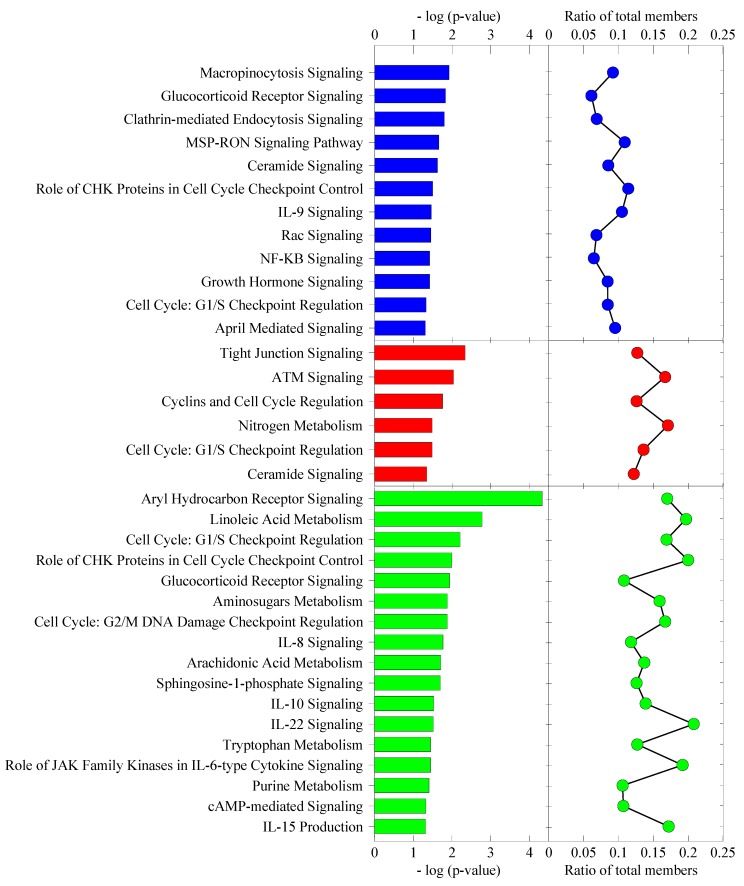
Classification of differentially expressed genes into various cellular stress response implicated canonical pathways. The [−log(*p*-value)] is the maximum *p*-value for all of the genes in each particular canonical pathway family. The ratio plot indicates the number of the genes expressed at each time point relative to the total number of genes in that particular canonical pathway group.

Subsequently, in order to highlight the pathways more relevant to cellular stress response, we further refined our analysis by excluding signaling pathway categories, such as cancer, cardiovascular and nervous system signaling, toxicity list and disease-specific pathways, in addition to non-skin cell-type-specific canonical pathways. Figure 10 shows a refined presentation of the top canonical pathways for each post-heat recovery time point. For the cell cycle, the G1/S checkpoint regulation pathway was affected in all three time points**. **The ceramide signaling pathway was affected in both 0 h and 4 h. The glucocorticoid receptor signaling pathway and role of CHK proteins in cell cycle checkpoint control were affected in both 0 h and 24 h. All the remaining canonical pathways involved were exclusive to a particular time point. Notably, although most of the canonical pathways in each post-heat time point were unique, they actually serve overlapping top cellular functions. The canonical pathways playing a role in cellular function and maintenance that were affected in 0 h post-heat consisted of MSP-RON signaling, Rac signaling pathways and two endocytosis pathways. Both endocytosis pathways, namely, macropinocytosis and clathrin-mediated endocytosis signaling pathways, play important roles in plasma membrane reorganization, internalization of extracellular molecules and in transduction of signals within the cell and between cells. Deregulation of seven and 13 genes in macropinocytosis signaling and clathrin-mediated endocytosis signaling, respectively, starting 0 h post-heat, implicate an immediate effect of heat shock on the plasma membrane and signal transduction. Macropinocytosis depends on signaling to the actin cytoskeleton, such as the Rac signaling pathway, which was also affected at 0 h post-heat, with eight deregulated genes, including the Rac activator, TIAM1 (T-lymphoma invasion and metastasis 1). Immediately, TIAM1 mRNA showed more than a 4-fold increase. Interestingly, TIAM1 has been reported to prevent cell death in heat-stressed wild-type murine keratinocytes, since the TIAM1 knock-out cells displayed increased apoptosis [20].

Of the canonical pathways affected at 4 h post-heat, tight junction signaling plays a role in cellular function and maintenance, whereas those affected at 24 h post-heat included amino sugar metabolism and cAMP-mediated signaling pathways. The latter is also important in controlling cellular assembly and organization in addition to post-translational modification. Notably, tight junction signaling also plays a role in cellular assembly and organization in addition to cell-to-cell signaling and interaction. The 0 h time-point post-heat affected pathways playing roles in the regulation of cellular development and/or in cellular growth and proliferation comprised cell cycle G1/S checkpoint regulation, the glucocorticoid receptor, IL-9, NF-κB, April-mediated and growth hormone signaling. The 24 h post-heat assessment showed that pathways regulating these functions are the cell cycle G1/S checkpoint regulation, the glucocorticoid receptor, IL-10 signaling, the role of JAK family kinases in IL-6-type cytokine signaling and purine metabolism, while affected pathways at 4 h post-heat included cell cycle G1/S checkpoint regulation only. The cell cycle control function is regulated by two pathways at 0 h post-heat, namely cell cycle G1/S checkpoint regulation and growth hormone signaling.

Whereas, four of the canonical pathways affected at 4 h post-heat (ATM signaling, cyclins, cell cycle regulation, nitrogen metabolism and cell cycle G1/S checkpoint regulation) function in the control of cell cycle progression. Six of the 17 canonical pathways affected at 24 h post-heat play a role in cell cycle control. These are aryl hydrocarbon receptor signaling, cell cycle G1/S checkpoint regulation, the role of CHK proteins in cell cycle checkpoint control, cell cycle G2/M DNA damage checkpoint regulation and the two metabolic pathways, linoleic acid metabolism and tryptophan metabolism. Cell death, on the other hand, is controlled by the ceramide signaling pathway that was affected at 0 and 4 h post-heat and by the related sphingosine-1-phosphate signaling pathway, as well as by the aryl hydrocarbon receptor signaling and cell cycle G2/M DNA damage checkpoint regulation, which were affected at 24 h post-heat. Interestingly, the canonical pathways involved in inflammatory response were only affected at 24 h post-heat. They consist of IL-10 and IL-22 signaling. The DNA replication, recombination and repair function involves the role of CHK proteins in cell cycle checkpoint control that was affected at 4 h and 24 h post-heat, the ATM signaling pathway that was affected at 4 h post-heat only and cell cycle G2/M DNA damage checkpoint regulation that was affected at 24 h post-heat only.

## 3. Experimental Section

### 3.1. Cell Culture Conditions and Hyperthermia Stress Protocol

HEK were cultured in EpiLife^®^ medium containing supplement S7, as per the manufacturer’s recommendations (Life Technologies). For the heat-shock experiment, cells were put in 1.7 mL tubes (1.0 × 10^6^ cells/tube) that were sealed with Para-film^®^ and placed in a circulating water bath at 44 °C for 40 min. No significant change in pH was observed. Following hyperthermia, cells were either harvested within 5 min or were first allowed to recover in the incubator at 37 °C/95% humidity/5% CO_2_ for 4 h or for 24 h. Control cells harvested alongside the heat-treated cells were also put in 1.7 mL tubes (1.0 × 10^6^ cells/tube), but were maintained at all time in an incubator at 37 °C/95% humidity/5% CO_2_.

### 3.2. Cellular Viability Assays

Viability was evaluated at different post-exposure time points using MTT (3-(4,5-dimethylthiazol-2-yl)-2,5-diphenyltetrazolium bromide) assays (ATCC, Manassas, VA, USA). In brief, HEKs were exposed to hyperthermic stress, then were incubated for 5 min (0 h post-heat), 4 h (4 h post-heat) or 24 h (24 h post-heat). Subsequently, MTT reagents and detergents were added, as per the manufacturer’s instructions. Absorbance readings were measured at 570 nm with a Synergy HT Plate Reader (Biotech, Winooski, VT, USA). Absorbance readings were also measured for a ladder of serial dilutions (10^3^–10^6^ cells) in culture medium. Absorbance values were plotted *versus* the cell number, and these curves were used to determine the number of viable cells in each well.

### 3.3. RNA Isolation and Normalization

RNA was extracted from the heat-treated and control cells using a RNeasy Mini-Kit (Qiagen), according to the manufactures instructions. RNA extraction was performed at three different time intervals as follows: within 5 min following heat-shock (0 h post-heat), after a 4 h recovery period at 37 °C (4 h post-heat) or after a 24 h recovery period at 37 °C (24 h post-heat). At each time point, the RNA concentration was assessed on a NanoDrop Spectrophotometer (NanoDrop Technologies), and the quality was measured on a 2100 Bioanalyzer™ (Agilent Technologies). RNA samples with a RNA Integrity Number greater than 9.5 were subjected to microarray analysis.

### 3.4. Microarray Data Analysis

Expression analysis was performed for each time point in triplicates (three control and three heat-shock treated) using the Affymetrix GeneChip^®^ Human Genome U133 (HG-U133) plus 2.0 Array that contains 54,675 probe sets. Briefly, two micrograms of RNA were used for preparation of biotin-labeled targets (cRNA) using MessageAmp™-based protocols (Ambion, Inc.). Labeled cRNA was fragmented (0.5 μg/μL per reaction) and was used for array hybridization and washing. The cRNA was mixed with a hybridization cocktail, heated to 99 °C for 5 min and then incubated at 45 °C for 5 min. Hybridization arrays were conducted for 16 h in an Affymetrix Model 640 hybridization oven (45 °C, 60 rpm). Arrays were washed and stained on an FS450 Fluidics station and were scanned on a GeneChip^®^ Scanner 3000 7G. Image signal data, detection calls and annotations were generated for every gene using the Affymetrix Statistical Algorithm MAS 5.0 (GeneChip^®^ Operating Software v1.3). A log_2_ transformation was conducted and a Student’s *t*-test was performed for comparison of the two groups (control and heat-shocked). We conducted multiple testing correction—Benjamini and Hochberg—to determine the false discovery rate, and statistical significant genes were identified using Bonferroni correction procedures (−log_10_ p_cutoff_ > 6.04) [21].

For interpretation of the results, the Ingenuity Pathways Analysis tool (IPA version 8.7, Ingenuity^®^ Systems Inc., Redwood City, CA, USA; [22]) was used. IPA is a web-based software application, which enables filtering and dataset comparisons, to identify biological mechanisms, pathways and functions most relevant to experimental datasets or differentially expressed genes. The cut-off criteria for our IPA analysis were: an absolute value of log_2_ ratio ≥1.5 and a *p*-value ≤0.05. Other web-based resources, such as the GeneCards Human Gene Database [23], The HUGO Gene Nomenclature Committee (HGNC, [24]) and the Gene Ontology [25] were also used to further supplement the analysis. The targets identified in the microarray study were validated using qRT-PCR. Runs were performed using TaqMan^®^ RNA-to-CT™ 1-Step Kits and TaqMan^®^ Assays for: HSPA6 and GAPDH (Applied Biosystems). Calibrator RNA was used as the control (Cell Applications Inc.). All assays were performed by Asuragen Services.

## 4. Conclusions

In this study, we examined the temporal gene expression kinetics for HEK cells exposed to hyperthermic stress. Collectively, our results demonstrate that hyperthermic stress triggers a rapid, robust and extensive transcriptional response in HEK cells. The temporal kinetics expression data show that this cellular response is immediately activated in response to stress and is sustained for many hours after exposure. Specifically, we show that the number of upregulated genes increased in logarithmic fashion as a function of time, ranging from 466 at 0 h, 872 at 4 h and up to 1,127 at 24 h. This finding is consistent with previous *in vivo* studies, which show that HSP70 expression is bi-phasic and peaks at both 12 h and 24 h post-exposure [5,7,8]. However, this data is in contrast to previous *in vitro* reports, which show that the number of regulated genes is maximal roughly 4 to 12 h post-exposure [3,4,5]. Furthermore, in this study, we show that many of the genes activated at 24 h encode for chemokines that play central roles in inflammation (*i.e.*, IL-8, IL-10, IL-22, IL-15 and JAK family kinases). This suggests that the second expression peak at 24 h is likely due to cellular inflammation.

We identified a large group of diverse heat-inducible genes in this work. The temporal kinetics data shows that each individual gene exhibits distinct expression profiles, which vary dramatically in magnitude, onset time and duration of expression. From the data, we determined that the heat-inducible genes can be grouped according to their temporal expression phases. The first group belongs to an immediate-expression phase consisting of genes that are rapidly expressed shortly (*i.e.*, 5–20 min) after exposure to hyperthermic stress. The second group consists of genes expressed several minutes up to 4 h post exposure, and the third group can be considered an “adaption or recovery phase” that occurs 4 h to 24 h post exposure.

The majority of genes expressed during the immediate expression phase encode for proteins involved in molecular transport, cellular growth and heat shock response. One notable molecular transport gene is HBA1/HBA2, which functions to facilitate the delivery of oxygen. Another interesting finding is that only the well characterized HSPs (*i.e.*, HSP40 and HSP70) are expressed at the early time points, while the smaller HSPs and chaperonins (HSPE1, HSP60; HSPE1, HSP10; HSPB2, HSP27) are expressed at roughly 24 h post-exposure. Furthermore, other HSPs, such as HSPH1 (HSP105), which function primarily to prevent protein aggregation, exhibit maximal expression at the 4 h time-point. This data strongly supports that each particular type of HSP exhibits expression kinetics that appear to be directly correlated with the function that is required at each time point. In summary, these data provide valuable new insights that give us a much clearer picture of the genes and intracellular signaling pathways that are triggered in human epithelial keratinocytes exposed to hyperthermic stress.

## Supplementary Files

Supplementary File 1 Supplementary Table (PDF, 2889 KB)
