# Supplementary material for: Temporal Gene Expression Kinetics for Human Keratinocytes Exposed to Hyperthermic Stress
Source: Cells. 2013 Apr 10;2(2):224–43. doi: 10.3390/cells2020224 (PMC3972685; doi:10.3390/cells2020224)
Supplement: Supplementary File 1 — Supplementary Table (PDF, 2889 KB) [file cells-02-00224-s001.pdf]

Supplementary Table 1. 492 genes are unique to 0 h post-heat timepoint. The name, p-value, fold change, location and family of each gene are indicated. Genes were filtered for an absolute value log2 ration ≥1.5 and a significance value of $p \leq 0.05$.

Symbol	p-value	Log Ratio	Gene Name	Location	Family
ABCA13	1.87E-02	3.292	ATP-binding cassette, sub-family A (ABC1), member 13	unknown	transporter
ABCB1	1.93E-02	−1.819	ATP-binding cassette, sub-family B (MDR/TAP), member 1	Plasma Membrane	transporter
ABCC3	2.83E-02	2.016	ATP-binding cassette, sub-family C (CFTR/MRP), member 3	Plasma Membrane	transporter
ABHD6	7.79E-03	−2.717	abhydrolase domain containing 6	Cytoplasm	enzyme
ACAT1	4.10E-02	3.009	acetyl-CoA acetyltransferase 1	Cytoplasm	enzyme
ACBD4	2.66E-03	1.722	acyl-CoA binding domain containing 4	unknown	other
ACSL5	1.86E-02	−2.876	acyl-CoA synthetase long-chain family member 5	Cytoplasm	enzyme
ADAM23	3.33E-02	−3.008	ADAM metallopeptidase domain 23	Plasma Membrane	peptidase
ADAM29	5.58E-03	3.463	ADAM metallopeptidase domain 29	Plasma Membrane	peptidase
ADAMTS17	2.67E-04	3.051	ADAM metallopeptidase with thrombospondin type 1 motif, 17	Extracellular Space	other
ADCYAP1R1	1.20E-02	1.848	adenylate cyclase activating polypeptide 1 (pituitary) receptor type I	Plasma Membrane	G-protein coupled receptor
ADH6 (includes EG:130)	4.02E-02	−1.845	alcohol dehydrogenase 6 (class V)	Cytoplasm	enzyme
AHSA2	1.54E-04	−1.6	AHA1, activator of heat shock 90kDa protein ATPase homolog 2 (yeast)	unknown	other
AK5	3.32E-02	1.658	adenylate kinase 5	Cytoplasm	kinase
AK7	4.13E-04	1.967	adenylate kinase 7	unknown	kinase
AKR1C1/AKR1C2	3.91E-04	−2.488	aldo-keto reductase family 1, member C2 (dihydrodiol dehydrogenase 2; bile acid binding protein; 3-alpha hydroxysteroid dehydrogenase, type III)	Cytoplasm	enzyme
ALK	4.28E-02	−2.332	anaplastic lymphoma receptor tyrosine kinase	Plasma Membrane	kinase
AMBP	9.89E-04	−1.57	alpha-1-microglobulin/bikunin precursor	Extracellular Space	transporter
ANKRD24	6.21E-03	3.017	ankyrin repeat domain 24	unknown	other
ANKRD33	4.85E-02	2.899	ankyrin repeat domain 33	Nucleus	transcription regulator
ANKS1B	4.35E-02	−3.129	ankyrin repeat and sterile alpha	Nucleus	other

			motif domain containing 1B		
AP3B2	4.27E-02	1.669	adaptor-related protein complex 3, beta 2 subunit	Cytoplasm	transporter
APBA3	3.14E-02	3.313	amyloid beta (A4) precursor protein-binding, family A, member 3	Cytoplasm	transporter
APC	2.71E-03	−1.643	adenomatous polyposis coli	Nucleus	enzyme
APOBR	4.90E-02	−1.59	apolipoprotein B receptor	Plasma Membrane	transmembrane receptor
APOM	9.55E-04	2.057	apolipoprotein M	Plasma Membrane	transporter
AQP2	3.06E-02	−3.041	aquaporin 2 (collecting duct)	Plasma Membrane	transporter
ARHGAP9	1.35E-02	1.559	Rho GTPase activating protein 9	Cytoplasm	other
ARID4B	1.90E-02	−1.844	AT rich interactive domain 4B (RBP1-like)	Nucleus	other
ARL10	7.68E-04	2.053	ADP-ribosylation factor-like 10	unknown	other
ASAH2B	2.72E-02	2.863	N-acylsphingosine amidohydrolase (non-lysosomal ceramidase) 2B	Cytoplasm	other
ASCL3	2.66E-03	2.198	achaete-scute complex homolog 3 (Drosophila)	Nucleus	transcription regulator
ASTN2	4.60E-03	3.203	astrotactin 2	unknown	other
ATP2B2	1.54E-02	2.707	ATPase, Ca++ transporting, plasma membrane 2	Plasma Membrane	transporter
ATP9A	5.77E-03	−1.882	ATPase, class II, type 9A	Plasma Membrane	transporter
B3GALT6	4.84E-02	−1.707	UDP-Gal:betaGal beta 1,3-galactosyltransferase polypeptide 6	Cytoplasm	enzyme
B9D2	9.06E-05	2.724	B9 protein domain 2	Cytoplasm	other
BCL2	4.93E-02	−1.516	B-cell CLL/lymphoma 2	Cytoplasm	transporter
BHMT2	1.81E-02	−2.498	betaine--homocysteine S-methyltransferase 2	Cytoplasm	enzyme
BNIP1	4.10E-03	1.545	BCL2/adenovirus E1B 19kDa interacting protein 1	Cytoplasm	other
BSND	4.82E-02	−2.09	Bartter syndrome, infantile, with sensorineural deafness (Barttin)	Plasma Membrane	ion channel
BTBD16	4.02E-03	3.285	BTB (POZ) domain containing 16	unknown	other
BTRC	1.85E-02	2.727	beta-transducin repeat containing	Cytoplasm	enzyme
C10orf12	3.79E-03	−2.205	chromosome 10 open reading frame 12	unknown	other
C11orf52	1.56E-02	2.011	chromosome 11 open reading frame 52	unknown	other
C12orf69	1.06E-04	4.046	chromosome 12 open reading	unknown	other

			frame 69		
C13orf30	4.45E-02	−2.442	chromosome 13 open reading frame 30	unknown	other
C14orf182	2.26E-02	−3.651	chromosome 14 open reading frame 182	unknown	other
C14orf166B	3.25E-02	−3.164	chromosome 14 open reading frame 166B	unknown	other
C17orf28	3.05E-02	−1.839	chromosome 17 open reading frame 28	Plasma Membrane	other
C17orf46	1.18E-02	2.921	chromosome 17 open reading frame 46	unknown	other
C17orf78	1.46E-02	3.47	chromosome 17 open reading frame 78	unknown	other
C17orf89	3.62E-02	1.989	chromosome 17 open reading frame 89	Cytoplasm	other
C17orf101	1.00E-02	2.716	chromosome 17 open reading frame 101	unknown	enzyme
C18orf42	2.83E-02	2.229	chromosome 18 open reading frame 42	unknown	other
C1orf63	5.28E-05	−2.281	chromosome 1 open reading frame 63	unknown	other
C1orf145	7.40E-03	2.595	chromosome 1 open reading frame 145	unknown	other
C1QTNF2	5.91E-04	−2.921	C1q and tumor necrosis factor related protein 2	Extracellular Space	other
C1QTNF4	3.79E-02	−1.532	C1q and tumor necrosis factor related protein 4	Extracellular Space	other
C20orf166-AS1	2.70E-02	1.846	C20orf166 antisense RNA 1 (non-protein coding)	unknown	other
C3orf15	4.11E-02	1.799	chromosome 3 open reading frame 15	Cytoplasm	other
C3orf58	4.47E-03	−3.439	chromosome 3 open reading frame 58	Cytoplasm	other
C3orf62	9.18E-03	−2.216	chromosome 3 open reading frame 62	unknown	other
C4B (includes others)	4.35E-02	1.738	complement component 4B (Chido blood group)	Extracellular Space	other
C6orf52	8.96E-03	1.589	chromosome 6 open reading frame 52	unknown	other
C6orf164	4.09E-02	−3.128	chromosome 6 open reading frame 164	unknown	other
C6orf201	2.99E-03	3.75	chromosome 6 open reading frame 201	unknown	other
C7orf53	1.21E-03	−2.63	chromosome 7 open reading frame 53	unknown	other
C8A	9.97E-04	1.862	complement component 8, alpha	Extracellular	other

			polypeptide	Space	
C8orf50	3.89E-03	2.171	chromosome 8 open reading frame 50	unknown	other
C8orf83	3.62E-02	−3.838	chromosome 8 open reading frame 83	unknown	other
CAB39L	2.41E-02	−1.594	calcium binding protein 39-like	Cytoplasm	kinase
CACNG2	3.73E-02	1.845	calcium channel, voltage-dependent, gamma subunit 2	Plasma Membrane	ion channel
CAMKMT	1.04E-02	2.453	calmodulin-lysine N-methyltransferase	unknown	other
CAPN5	2.67E-02	2.793	calpain 5	Cytoplasm	peptidase
CAPS	1.23E-02	1.838	calcyphosine	Cytoplasm	other
CARM1	2.38E-02	−2.128	coactivator-associated arginine methyltransferase 1	Nucleus	transcription regulator
CATSPERB	2.26E-02	−1.943	cation channel, sperm-associated, beta	Plasma Membrane	other
CBFA2T2	5.50E-04	3.259	core-binding factor, runt domain, alpha subunit 2; translocated to, 2	Nucleus	transcription regulator
CCDC40	3.75E-02	−2.049	coiled-coil domain containing 40	unknown	other
CCDC83	4.83E-02	−1.787	coiled-coil domain containing 83	unknown	other
CCDC74B	1.41E-02	1.587	coiled-coil domain containing 74B	unknown	other
CCL16	2.96E-02	−2.929	chemokine (C-C motif) ligand 16	Extracellular Space	cytokine
CD7	4.56E-02	−1.898	CD7 molecule	Plasma Membrane	other
CD46	6.62E-03	3.14	CD46 molecule, complement regulatory protein	Plasma Membrane	other
CDH6	2.36E-02	−2.424	cadherin 6, type 2, K-cadherin (fetal kidney)	Plasma Membrane	other
CDH23	3.78E-02	−2.639	cadherin-related 23	Plasma Membrane	transporter
CES1	4.24E-02	−2.128	carboxylesterase 1	Cytoplasm	enzyme
CHIC1	3.68E-02	2.427	cysteine-rich hydrophobic domain 1	Plasma Membrane	other
CLEC11A	2.18E-04	3.368	C-type lectin domain family 11, member A	Extracellular Space	growth factor
CLEC2L	2.44E-03	2.46	C-type lectin domain family 2, member L	unknown	other
CLEC7A	1.33E-02	−2.047	C-type lectin domain family 7, member A	Plasma Membrane	transmembrane receptor
CLGN	2.70E-03	3.396	calmegin	Cytoplasm	peptidase
CMTM7	9.23E-03	1.558	CKLF-like MARVEL transmembrane domain containing 7	Extracellular Space	cytokine
CNGB1	2.61E-02	−1.917	cyclic nucleotide gated channel	Plasma	ion channel

			beta 1	Membrane	
CNPPD1	8.17E-03	−2.056	cyclin Pas1/PHO80 domain containing 1	unknown	other
COG1 (includes EG:100334475)	4.09E-03	1.559	component of oligomeric golgi complex 1	Cytoplasm	transporter
COL4A3	3.49E-02	−2.331	collagen, type IV, alpha 3 (Goodpasture antigen)	Extracellular Space	other
CORO1A	4.18E-02	−2.029	coronin, actin binding protein, 1A	Cytoplasm	other
CR1	4.71E-02	1.76	complement component (3b/4b) receptor 1 (Knops blood group)	Plasma Membrane	other
CRYGA	2.55E-02	2.524	crystallin, gamma A	unknown	other
CWF19L2	1.12E-02	2.78	CWF19-like 2, cell cycle control (S. pombe)	unknown	other
CYP4F3	2.01E-02	1.607	cytochrome P450, family 4, subfamily F, polypeptide 3	Cytoplasm	enzyme
DAPK2	5.80E-03	2.304	death-associated protein kinase 2	Cytoplasm	kinase
DDX11/DDX12P	1.42E-02	2.426	DEAD/H (Asp-Glu-Ala-Asp/His) box polypeptide 11	Nucleus	enzyme
DEFB125	3.89E-02	−2.499	defensin, beta 125	Extracellular Space	other
DENND4C	2.76E-02	3.533	DENN/MADD domain containing 4C	unknown	other
DENND5B	1.70E-03	2.326	DENN/MADD domain containing 5B	unknown	other
DIP2A	1.12E-02	2.347	DIP2 disco-interacting protein 2 homolog A (Drosophila)	Nucleus	transcription regulator
DLEC1	1.09E-02	−1.612	deleted in lung and esophageal cancer 1	Cytoplasm	other
DLG2	1.31E-02	−2.612	discs, large homolog 2 (Drosophila)	Plasma Membrane	kinase
DLG5	7.62E-03	1.634	discs, large homolog 5 (Drosophila)	Plasma Membrane	other
DLGAP3	3.71E-03	−1.802	discs, large (Drosophila) homolog-associated protein 3	Cytoplasm	other
DNAH1	4.99E-02	2.509	dynein, axonemal, heavy chain 1	unknown	other
DNAJC4	1.40E-03	4.175	DnaJ (Hsp40) homolog, subfamily C, member 4	Cytoplasm	other
DOCK5	1.34E-04	−3.227	dedicator of cytokinesis 5	Cytoplasm	other
DPYSL4	1.36E-02	1.773	dihydropyrimidinase-like 4	Cytoplasm	enzyme
DRP2	4.65E-02	1.945	dystrophin related protein 2	Plasma Membrane	other
DSCAML1	3.62E-02	2.884	Down syndrome cell adhesion molecule like 1	Plasma Membrane	other
DUOX1	4.96E-02	−2.692	dual oxidase 1	Plasma Membrane	enzyme
EFEMP1	4.99E-02	−2.011	EGF containing fibulin-like	Extracellular	enzyme

			extracellular matrix protein 1	Space	
EIF4ENIF1	2.00E-02	2.183	eukaryotic translation initiation factor 4E nuclear import factor 1	Cytoplasm	translation regulator
EPHB6	2.06E-05	2.989	EPH receptor B6	Plasma Membrane	kinase
ESYT3	4.97E-02	2.836	extended synaptotagmin-like protein 3	unknown	other
EVC2	1.66E-04	1.616	Ellis van Creveld syndrome 2	unknown	other
FAH	3.35E-03	−2.04	fumarylacetoacetate hydrolase (fumarylacetoacetase)	Cytoplasm	enzyme
FAM120A	3.99E-02	4.262	family with sequence similarity 120A	Cytoplasm	other
FAM120AOS	3.49E-03	2.756	family with sequence similarity 120A opposite strand	unknown	other
FAM150B	2.94E-02	−2.858	family with sequence similarity 150, member B	unknown	other
FAM195A	2.24E-02	−2.26	family with sequence similarity 195, member A	unknown	other
FAM26F	9.22E-03	−2.162	family with sequence similarity 26, member F	unknown	other
FAM43A	8.20E-03	−2.316	family with sequence similarity 43, member A	unknown	other
FAM66D	2.86E-03	−3.284	family with sequence similarity 66, member D	unknown	other
FAM69A	1.32E-03	−1.584	family with sequence similarity 69, member A	unknown	other
FAM71A	2.58E-02	1.883	family with sequence similarity 71, member A	unknown	other
FAM91A2	3.92E-02	2.312	family with sequence similarity 91, member A2	unknown	other
FBLN1	3.31E-03	3.535	fibulin 1	Extracellular Space	other
FBXL13	7.61E-03	2.092	F-box and leucine-rich repeat protein 13	unknown	enzyme
FBXO21	2.34E-02	−1.788	F-box protein 21	unknown	enzyme
FDCSP	4.86E-02	3.133	follicular dendritic cell secreted protein	Extracellular Space	other
FKBP7	1.21E-02	2.303	FK506 binding protein 7	Cytoplasm	enzyme
FLJ11235	4.23E-02	3.184	uncharacterized FLJ11235	unknown	other
FLJ25917	3.49E-02	2.801	uncharacterized LOC401585	unknown	other
FMNL3	6.69E-03	2.914	formin-like 3	Cytoplasm	other
FOXP1	3.79E-03	3.577	forkhead box P1	Nucleus	transcription regulator
FOXP2	3.95E-02	2.832	forkhead box P2	Nucleus	transcription regulator
FSHB	3.63E-02	−2.96	follicle stimulating hormone, beta	Extracellular	other

			polypeptide	Space	
FUCA1	6.07E-03	2.573	fucosidase, alpha-L- 1, tissue	Cytoplasm	enzyme
FUT10	6.14E-03	1.921	fucosyltransferase 10 (alpha (1,3) fucosyltransferase)	Cytoplasm	enzyme
GAD2	4.68E-02	−2.819	glutamate decarboxylase 2 (pancreatic islets and brain, 65kDa)	Cytoplasm	enzyme
GALNTL1	4.31E-02	2.912	UDP-N-acetyl-alpha-D-galactosamine:polypeptide N-acetylgalactosaminyltransferase-like 1	Cytoplasm	enzyme
GAMT	4.12E-03	−1.638	guanidinoacetate N-methyltransferase	Cytoplasm	enzyme
GAS7	1.43E-03	1.724	growth arrest-specific 7	Cytoplasm	transcription regulator
GATM	1.67E-02	2.34	glycine amidinotransferase (L-arginine:glycine amidinotransferase)	Cytoplasm	enzyme
GATS	1.82E-02	2.03	GATS, stromal antigen 3 opposite strand	unknown	other
GC	2.00E-02	3.955	group-specific component (vitamin D binding protein)	Extracellular Space	transporter
GIMAP4	1.32E-02	−2.412	GTPase, IMAP family member 4	Nucleus	other
GIP	4.90E-02	1.517	gastric inhibitory polypeptide	Extracellular Space	other
GIPR	2.86E-02	−2.149	gastric inhibitory polypeptide receptor	Plasma Membrane	G-protein coupled receptor
GIT2	3.79E-02	2.398	G protein-coupled receptor kinase interacting ArfGAP 2	Nucleus	other
GLCCI1	3.25E-02	−1.623	glucocorticoid induced transcript 1	Cytoplasm	other
GLI4	3.62E-04	1.749	GLI family zinc finger 4	Nucleus	other
GLRA2	3.95E-02	−2.921	glycine receptor, alpha 2	Plasma Membrane	ion channel
GP9	1.44E-02	−1.936	glycoprotein IX (platelet)	Plasma Membrane	other
GPR116	8.50E-03	−2.6	G protein-coupled receptor 116	Plasma Membrane	G-protein coupled receptor
GPR123	1.94E-02	−2.106	G protein-coupled receptor 123	Plasma Membrane	G-protein coupled receptor
GREB1L	4.29E-02	−1.533	growth regulation by estrogen in breast cancer-like	unknown	other
GRIN3A	1.21E-02	2.093	glutamate receptor, ionotropic,	Plasma	ion channel

			N-methyl-D-aspartate 3A	Membrane	
GRM3	2.28E-02	2.542	glutamate receptor, metabotropic 3	Plasma Membrane	G-protein coupled receptor
GUCA1A	1.74E-03	1.843	guanylate cyclase activator 1A (retina)	Cytoplasm	other
GYPC	3.56E-02	−1.724	glycophorin C (Gerbich blood group)	Plasma Membrane	other
HAND2	3.72E-03	−1.609	heart and neural crest derivatives expressed 2	Nucleus	transcription regulator
HBE1	6.10E-03	−1.927	hemoglobin, epsilon 1	Cytoplasm	transporter
HDC	2.51E-05	2.211	histidine decarboxylase	Cytoplasm	enzyme
HEATR5B	2.12E-02	3.011	HEAT repeat containing 5B	unknown	other
HEATR7A	2.20E-03	1.674	HEAT repeat containing 7A	unknown	other
HIRIP3	8.81E-04	2.317	HIRA interacting protein 3	Nucleus	other
HIST1H1T	1.53E-02	1.679	histone cluster 1, H1t	Nucleus	other
HLA-DOA	2.65E-02	−3.016	major histocompatibility complex, class II, DO alpha	Plasma Membrane	transmembrane receptor
HNRPDL	4.57E-02	−1.864	heterogeneous nuclear ribonucleoprotein D-like	Nucleus	other
HOXB13	1.01E-02	−2.24	homeobox B13	Nucleus	transcription regulator
HRASLS	4.65E-02	3.25	HRAS-like suppressor	Cytoplasm	other
HS3ST6	4.16E-02	1.83	heparan sulfate (glucosamine) 3-O-sulfotransferase 6	Cytoplasm	enzyme
HSD11B1	2.27E-02	2.212	hydroxysteroid (11-beta) dehydrogenase 1	Cytoplasm	enzyme
HSPA12B	2.61E-02	2.806	heat shock 70kD protein 12B	unknown	other
IAPP	2.26E-02	−1.621	islet amyloid polypeptide	Extracellular Space	other
IDH1	2.36E-04	3.445	isocitrate dehydrogenase 1 (NADP+), soluble	Cytoplasm	enzyme
IFNA1/IFNA13	4.39E-02	−3.042	interferon, alpha 1	Extracellular Space	cytokine
IGF2BP3	4.60E-02	2.928	insulin-like growth factor 2 mRNA binding protein 3	Cytoplasm	translation regulator
IGHMBP2	6.25E-03	1.582	immunoglobulin mu binding protein 2	Nucleus	enzyme
INE2	3.29E-02	1.846	inactivation escape 2 (non-protein coding)	unknown	other
ING1	3.01E-02	−1.648	inhibitor of growth family, member 1	Nucleus	transcription regulator
INSL6	3.61E-02	2.21	insulin-like 6	Extracellular Space	other
INSR	4.76E-02	1.705	insulin receptor	Plasma Membrane	kinase

INTS4	3.26E-05	4.789	integrator complex subunit 4	Nucleus	other
IST1	4.74E-02	2.797	increased sodium tolerance 1 homolog (yeast)	Cytoplasm	other
ITGB3	1.60E-02	4.383	integrin, beta 3 (platelet glycoprotein IIIa, antigen CD61)	Plasma Membrane	transmembrane receptor
ITGB1BP2	1.16E-02	2.487	integrin beta 1 binding protein (melusin) 2	unknown	other
ITM2C	1.64E-03	1.976	integral membrane protein 2C	Cytoplasm	other
ITSN1	8.31E-03	1.626	intersectin 1 (SH3 domain protein)	Cytoplasm	other
JAZF1-AS1	1.69E-02	−2.571	JAZF1 antisense RNA 1 (non-protein coding)	unknown	other
JMJD1C	3.48E-02	2.378	jumonji domain containing 1C	Nucleus	other
KATNAL2	4.81E-02	2.387	katanin p60 subunit A-like 2	unknown	other
KCNJ4	3.82E-02	−2.155	potassium inwardly-rectifying channel, subfamily J, member 4	Plasma Membrane	ion channel
KCNQ4	3.08E-02	2.295	potassium voltage-gated channel, KQT-like subfamily, member 4	Plasma Membrane	ion channel
KHDRBS2	4.58E-02	2.097	KH domain containing, RNA binding, signal transduction associated 2	Nucleus	other
KIAA0825	1.25E-03	−1.973	KIAA0825	unknown	other
KIAA1683	8.02E-04	−1.611	KIAA1683	Cytoplasm	other
KIF16B	4.56E-03	1.525	kinesin family member 16B	Cytoplasm	enzyme
KLHL20	4.79E-02	2.213	kelch-like 20 (Drosophila)	Plasma Membrane	enzyme
KLRF1	3.97E-02	2.076	killer cell lectin-like receptor subfamily F, member 1	Plasma Membrane	transmembrane receptor
KRTAP4-1	7.60E-03	1.543	keratin associated protein 4-1	unknown	other
KRTAP4-12	4.91E-02	−1.897	keratin associated protein 4-12	unknown	other
LAMA5	3.93E-02	−2.186	laminin, alpha 5	Extracellular Space	other
LCK	1.16E-03	2.004	lymphocyte-specific protein tyrosine kinase	Cytoplasm	kinase
LDB3	4.26E-02	−1.614	LIM domain binding 3	Cytoplasm	transporter
LILRB4	3.02E-03	2.688	leukocyte immunoglobulin-like receptor, subfamily B (with TM and ITIM domains), member 4	unknown	other
LIM2	1.61E-04	2.936	lens intrinsic membrane protein 2, 19kDa	Plasma Membrane	other
LIN7B	4.47E-03	−2.748	lin-7 homolog B (C. elegans)	Cytoplasm	other
LINC00277	8.22E-03	2.023	long intergenic non-protein coding RNA 277	unknown	other
LINC00474	3.49E-02	1.522	long intergenic non-protein coding RNA 474	unknown	other
LMF2	1.58E-02	−1.566	lipase maturation factor 2	unknown	other

LMNA	4.26E-02	−2.083	lamin A/C	Nucleus	other
LOC93444	1.55E-03	−1.793	uncharacterized LOC93444	unknown	other
LOC127841	4.21E-02	−1.67	uncharacterized LOC127841	unknown	other
LOC142937	2.61E-02	3.566	uncharacterized protein BC008131	unknown	other
LOC145837	4.58E-02	−3.355	uncharacterized LOC145837	unknown	other
LOC146513	4.96E-02	−1.654	uncharacterized LOC146513	unknown	other
LOC153682	4.99E-02	1.739	uncharacterized LOC153682	unknown	other
LOC157273	1.73E-02	−2.339	uncharacterized LOC157273	unknown	other
LOC157503	1.49E-04	2.763	uncharacterized LOC157503	unknown	other
LOC158696	4.16E-02	2.823	uncharacterized LOC158696	unknown	other
LOC282980	1.26E-02	3.967	uncharacterized LOC282980	unknown	other
LOC283856	2.08E-02	2.651	uncharacterized LOC283856	unknown	other
LOC283861	1.90E-02	−1.745	uncharacterized LOC283861	unknown	other
LOC284263	4.05E-02	3.115	uncharacterized LOC284263	unknown	other
LOC284661	2.88E-02	−3.001	uncharacterized LOC284661	unknown	other
LOC285326	3.53E-02	2.747	uncharacterized LOC285326	unknown	other
LOC338817	2.07E-02	−3.267	uncharacterized LOC338817	unknown	other
LOC415056	4.67E-02	2.147	uncharacterized LOC415056	unknown	other
LOC440117	4.40E-02	−2.436	uncharacterized LOC440117	unknown	other
LOC441086	1.50E-04	3.282	uncharacterized LOC441086	unknown	other
LOC642620	4.07E-02	2.386	hCG1999814	unknown	other
LOC643648	1.60E-03	2.209	uncharacterized LOC643648	unknown	other
LOC728819	4.69E-03	1.893	hCG1645220	unknown	other
LOC100128071	1.56E-03	−2.332	uncharacterized LOC100128071	unknown	other
LOC100128840	3.29E-02	2.072	uncharacterized LOC100128840	unknown	other
LOC100129620	5.24E-03	−3.445	uncharacterized LOC100129620	unknown	other
LOC100133920	5.97E-03	2.575	uncharacterized LOC100133920	unknown	other
LOC100288238	3.95E-04	4.524	uncharacterized LOC100288238	unknown	other
LOC100289061	1.82E-02	−1.641	uncharacterized LOC100289061	unknown	other
LOC100506190	2.49E-02	2.961	uncharacterized LOC100506190	unknown	other
LOC100506195	2.94E-03	−2.081	uncharacterized LOC100506195	unknown	other
LOC100506462	2.13E-03	2.206	uncharacterized LOC100506462	unknown	other
LOC100506667	3.83E-02	1.82	uncharacterized LOC100506667	unknown	other
LOC100507389	5.49E-03	1.617	uncharacterized LOC100507389	unknown	other
LPAR4	2.49E-02	3.145	lysophosphatidic acid receptor 4	Plasma Membrane	G-protein coupled receptor
LRRC27	2.49E-02	2.618	leucine rich repeat containing 27	unknown	other
LRRC43	2.37E-02	1.545	leucine rich repeat containing 43	unknown	other
LTN1	3.38E-02	1.998	listerin E3 ubiquitin protein ligase 1	Nucleus	enzyme
LUZP2	3.77E-02	1.936	leucine zipper protein 2	unknown	other
LZTS2	2.37E-02	2.598	leucine zipper, putative tumor suppressor 2	Cytoplasm	other
MAGEA4	1.09E-02	2.798	melanoma antigen family A, 4	Cytoplasm	other

MAGEA5	7.25E-04	2.679	melanoma antigen family A, 5	unknown	other
MAGEA3/MAGEA6	1.51E-03	−1.704	melanoma antigen family A, 3	unknown	other
MARVELD1	3.94E-02	3.101	MARVEL domain containing 1	unknown	other
MAST1	3.96E-02	−1.522	microtubule associated serine/threonine kinase 1	Cytoplasm	kinase
MAST3	1.01E-02	2.142	microtubule associated serine/threonine kinase 3	unknown	kinase
MB	2.40E-02	−1.893	myoglobin	Cytoplasm	transporter
MDH1B	3.53E-02	2.699	malate dehydrogenase 1B, NAD (soluble)	unknown	enzyme
MEG8	4.56E-03	3.222	maternally expressed 8 (non-protein coding)	unknown	other
METTL19	1.34E-03	2.396	methyltransferase like 19	unknown	other
MGAT4C	4.55E-02	3.028	mannosyl (alpha-1,3-)-glycoprotein beta-1,4-N-acetylglucosaminyltransferase, isozyme C (putative)	Cytoplasm	enzyme
MGC12982	2.05E-02	2.728	uncharacterized protein MGC12982	unknown	other
MGC14436	2.77E-02	3.198	uncharacterized LOC84983	unknown	other
MGC15885	2.42E-03	1.845	uncharacterized protein MGC15885	unknown	other
MITF	1.57E-02	2.082	microphthalmia-associated transcription factor	Nucleus	transcription regulator
MLLT4	3.79E-03	4.476	myeloid/lymphoid or mixed-lineage leukemia (trithorax homolog, Drosophila); translocated to, 4	Nucleus	other
MPZ	3.02E-04	2.366	myelin protein zero	Plasma Membrane	other
MRS2 (includes EG:380836)	3.42E-02	3.398	MRS2 magnesium homeostasis factor homolog (S. cerevisiae)	Cytoplasm	transporter
MS4A14	2.97E-03	2.142	membrane-spanning 4-domains, subfamily A, member 14	unknown	other
MSH4	1.34E-02	3.314	mutS homolog 4 (E. coli)	Nucleus	enzyme
MSRA	4.36E-02	2.348	methionine sulfoxide reductase A	Cytoplasm	enzyme
MST1 (includes EG:15235)	3.73E-02	−2.026	macrophage stimulating 1 (hepatocyte growth factor-like)	Extracellular Space	growth factor
MSTN	7.91E-03	4.178	myostatin	Extracellular Space	growth factor
MT4	4.56E-02	1.527	metallothionein 4	unknown	other
MTSS1	3.46E-03	1.978	metastasis suppressor 1	Cytoplasm	other
MUC3A	1.05E-02	1.501	mucin 3A, cell surface associated	Extracellular Space	other
MYH2	2.69E-03	3.711	myosin, heavy chain 2, skeletal muscle, adult	Cytoplasm	enzyme

MYLPF	4.74E-02	1.762	myosin light chain, phosphorylatable, fast skeletal muscle	Cytoplasm	other
MYNN	1.75E-02	2.471	myoneurin	Nucleus	transcription regulator
MYOM3	3.74E-02	1.666	myomesin family, member 3	unknown	other
MYRIP	2.97E-02	−2.76	myosin VIIA and Rab interacting protein	Cytoplasm	other
N4BP2	6.37E-03	2.04	NEDD4 binding protein 2	Cytoplasm	kinase
N4BP2L1	3.72E-02	−2.334	NEDD4 binding protein 2-like 1	unknown	other
NAT8	2.32E-02	−1.925	N-acetyltransferase 8 (GCN5-related, putative)	Nucleus	transcription regulator
NAT16	6.16E-03	−2.725	N-acetyltransferase 16 (GCN5-related, putative)	unknown	other
NAV3	2.74E-02	1.759	neuron navigator 3	Nucleus	other
NCAM1	1.18E-02	−1.835	neural cell adhesion molecule 1	Plasma Membrane	other
NCAN	6.86E-03	1.827	neurocan	Extracellular Space	other
NCOA2	7.21E-03	−1.967	nuclear receptor coactivator 2	Nucleus	transcription regulator
NDST4	1.06E-02	4.399	N-deacetylase/N-sulfotransferase (heparan glucosaminyl) 4	Cytoplasm	enzyme
NEU3	2.10E-05	2.4	sialidase 3 (membrane sialidase)	Plasma Membrane	enzyme
NFATC2	1.38E-02	−3.231	nuclear factor of activated T-cells, cytoplasmic, calcineurin-dependent 2	Nucleus	transcription regulator
NFIA	3.83E-02	−1.999	nuclear factor I/A	Nucleus	transcription regulator
NFKB1	4.18E-02	1.973	nuclear factor of kappa light polypeptide gene enhancer in B-cells 1	Nucleus	transcription regulator
NHLRC3	2.14E-02	1.927	NHL repeat containing 3	unknown	other
NOC2L	2.87E-02	2.45	nucleolar complex associated 2 homolog (S. cerevisiae)	Nucleus	transcription regulator
NOXRED1	1.33E-05	2.363	NADP-dependent oxidoreductase domain containing 1	unknown	other
NPAS2	2.24E-02	−2.362	neuronal PAS domain protein 2	Nucleus	transcription regulator
NPHP3-AS1	4.87E-02	4.279	NPHP3 antisense RNA 1 (non-protein coding)	unknown	other
NPVF	4.27E-02	−2.686	neuropeptide VF precursor	Extracellular Space	other
NR1I3	4.48E-02	1.738	nuclear receptor subfamily 1, group I, member 3	Nucleus	ligand-dependent

					nuclear receptor
NR4A1	7.66E-03	−1.631	nuclear receptor subfamily 4, group A, member 1	Nucleus	ligand-dependent nuclear receptor
NR4A3	8.60E-03	−3.024	nuclear receptor subfamily 4, group A, member 3	Nucleus	ligand-dependent nuclear receptor
NRXN1	3.45E-03	−3.342	neurexin 1	Plasma Membrane	transporter
NTNG2	3.36E-02	3.348	netrin G2	Plasma Membrane	other
NXF5	3.75E-02	−1.899	nuclear RNA export factor 5	Nucleus	other
NXF2/NXF2B	1.48E-03	1.518	nuclear RNA export factor 2	Nucleus	transporter
OCIAD1	1.96E-02	−2.994	OCIA domain containing 1	Cytoplasm	other
ODF3	2.89E-02	−3.372	outer dense fiber of sperm tails 3	Cytoplasm	other
ODF2L	4.18E-02	2.666	outer dense fiber of sperm tails 2-like	Cytoplasm	other
OK/SW-CL.58	1.68E-05	3.404	OK/SW-CL.58	unknown	other
OPA1	1.39E-02	1.997	optic atrophy 1 (autosomal dominant)	Cytoplasm	enzyme
OPRK1	5.66E-03	2.546	opioid receptor, kappa 1	Plasma Membrane	G-protein coupled receptor
OPRM1	4.08E-02	2.335	opioid receptor, mu 1	Plasma Membrane	G-protein coupled receptor
OR12D3	1.71E-02	3.066	olfactory receptor, family 12, subfamily D, member 3	Plasma Membrane	G-protein coupled receptor
OR13C4	2.92E-02	2.359	olfactory receptor, family 13, subfamily C, member 4	Plasma Membrane	G-protein coupled receptor
OR2F1	3.76E-02	1.804	olfactory receptor, family 2, subfamily F, member 1	Plasma Membrane	G-protein coupled receptor
OR2W1	1.90E-03	2.127	olfactory receptor, family 2, subfamily W, member 1	Plasma Membrane	G-protein coupled receptor
OR51B5	2.63E-02	2.638	olfactory receptor, family 51, subfamily B, member 5	Plasma Membrane	other
OR7C1	2.35E-02	2.866	olfactory receptor, family 7, subfamily C, member 1	Plasma Membrane	G-protein coupled receptor

ORF1	1.47E-02	2.461	uncharacterized protein, clone pT-Adv JuaX22	unknown	other
OSGEP	3.10E-02	1.503	O-sialoglycoprotein endopeptidase	unknown	peptidase
P2RX5	4.32E-03	2.459	purinergic receptor P2X, ligand-gated ion channel, 5	Plasma Membrane	ion channel
PACRG	2.75E-03	2.275	PARK2 co-regulated	Cytoplasm	other
PADI2	1.59E-02	1.807	peptidyl arginine deiminase, type II	Cytoplasm	enzyme
PAH	4.43E-02	−1.702	phenylalanine hydroxylase	Cytoplasm	enzyme
PAPOLG	2.68E-02	2.008	poly(A) polymerase gamma	Nucleus	enzyme
PCDH20	2.03E-02	2.982	protocadherin 20	unknown	other
PCP4L1	3.24E-04	3.28	Purkinje cell protein 4 like 1	unknown	other
PCSK7	1.67E-03	−1.686	proprotein convertase subtilisin/kexin type 7	Cytoplasm	peptidase
PDCD4	6.15E-04	−1.892	programmed cell death 4 (neoplastic transformation inhibitor)	Nucleus	other
PDCL2	1.29E-02	−4.623	phosducin-like 2	unknown	other
PDPR	2.49E-02	2.794	pyruvate dehydrogenase phosphatase regulatory subunit	Cytoplasm	enzyme
PDZD2	2.88E-02	−1.972	PDZ domain containing 2	Plasma Membrane	other
PEBP4	2.42E-02	1.655	phosphatidylethanolamine-binding protein 4	Cytoplasm	other
PET112	2.17E-03	−1.69	PET112 homolog (yeast)	Cytoplasm	translation regulator
PHIP	2.98E-02	−3.346	pleckstrin homology domain interacting protein	Nucleus	other
PHLDB3	2.66E-02	−2.079	pleckstrin homology-like domain, family B, member 3	unknown	other
PIK3CG	6.20E-04	3.782	phosphoinositide-3-kinase, catalytic, gamma polypeptide	Cytoplasm	kinase
PLD1	8.16E-04	2.948	phospholipase D1, phosphatidylcholine-specific	Cytoplasm	enzyme
PLD4	3.05E-02	2.638	phospholipase D family, member 4	unknown	enzyme
PLIN5	4.23E-02	1.778	perilipin 5	Plasma Membrane	other
PLK4	3.96E-02	−2.218	polo-like kinase 4	Cytoplasm	kinase
PLXNA4	1.50E-02	2.476	plexin A4	Plasma Membrane	transmembrane receptor
POLR2A	2.50E-02	1.543	polymerase (RNA) II (DNA directed) polypeptide A, 220kDa	Nucleus	enzyme
POU2F1	3.30E-02	2.523	POU class 2 homeobox 1	Nucleus	transcription regulator

PPBP	3.90E-02	2.778	pro-platelet basic protein (chemokine (C-X-C motif) ligand 7)	Extracellular Space	cytokine
PPP1R10	5.03E-03	−2.102	protein phosphatase 1, regulatory subunit 10	Nucleus	other
PRDM5	1.55E-02	2.351	PR domain containing 5	Nucleus	other
PRKCB	1.21E-03	2.107	protein kinase C, beta	Cytoplasm	kinase
PRKD1	3.57E-03	2.46	protein kinase D1	Cytoplasm	kinase
PROP1	4.20E-02	−2.832	PROP paired-like homeobox 1	Nucleus	transcription regulator
PRR4	5.76E-03	2.537	proline rich 4 (lacrimal)	Extracellular Space	other
PRRT1	1.55E-02	−1.937	proline-rich transmembrane protein 1	unknown	other
PSORS1C3	6.45E-03	−2.753	psoriasis susceptibility 1 candidate 3 (non-protein coding)	unknown	other
PTGER2	4.85E-02	−2.874	prostaglandin E receptor 2 (subtype EP2), 53kDa	Plasma Membrane	G-protein coupled receptor
PTH	3.86E-02	2.929	parathyroid hormone	Extracellular Space	other
PTPN7	4.02E-02	1.974	protein tyrosine phosphatase, non-receptor type 7	Cytoplasm	phosphatase
PUS10	4.12E-02	2.653	pseudouridylate synthase 10	unknown	other
QTRTD1	1.15E-02	−2.501	queuine tRNA-ribosyltransferase domain containing 1	Cytoplasm	other
RASA4/RASA4B	4.99E-02	−1.563	RAS p21 protein activator 4	Cytoplasm	other
RBKS	5.99E-04	−3.194	ribokinase	unknown	kinase
RBM20	4.63E-02	2.025	RNA binding motif protein 20	unknown	other
RDH10	1.44E-02	−4.364	retinol dehydrogenase 10 (all-trans)	Nucleus	enzyme
RDM1	1.19E-02	1.785	RAD52 motif 1	unknown	other
REXO2	1.00E-03	2.428	REX2, RNA exonuclease 2 homolog (S. cerevisiae)	Cytoplasm	enzyme
RGS11	3.38E-02	−2.413	regulator of G-protein signaling 11	Plasma Membrane	enzyme
RMND5A	2.18E-02	1.945	required for meiotic nuclear division 5 homolog A (S. cerevisiae)	unknown	other
RNF14	5.64E-03	3.073	ring finger protein 14	Nucleus	transcription regulator
ROBO2	2.52E-02	−3.64	roundabout, axon guidance receptor, homolog 2 (Drosophila)	Plasma Membrane	transmembrane receptor
RPS6	2.82E-04	−1.707	ribosomal protein S6	Cytoplasm	other
RPS14	1.98E-02	−3.185	ribosomal protein S14	Cytoplasm	translation regulator

RXFP1	1.85E-02	3.607	relaxin/insulin-like family peptide receptor 1	Plasma Membrane	G-protein coupled receptor
S100A6	2.87E-03	−1.775	S100 calcium binding protein A6	Cytoplasm	transporter
SAMD5	4.42E-02	2.107	sterile alpha motif domain containing 5	unknown	other
SCGB1A1	1.91E-04	3.147	secretoglobin, family 1A, member 1 (uteroglobin)	Extracellular Space	cytokine
SCN9A	2.48E-02	−3.674	sodium channel, voltage-gated, type IX, alpha subunit	Plasma Membrane	ion channel
SDCCAG3	5.83E-03	4.801	serologically defined colon cancer antigen 3	unknown	other
SDHAP3	2.11E-02	−1.964	succinate dehydrogenase complex, subunit A, flavoprotein pseudogene 3	unknown	other
SDR9C7	4.81E-04	2.798	short chain dehydrogenase/reductase family 9C, member 7	unknown	enzyme
4-Sep	3.82E-02	−1.631	septin 4	Cytoplasm	enzyme
SEPT7P2	3.27E-03	−2.55	septin 7 pseudogene 2	unknown	other
SERHL	3.17E-02	−1.635	serine hydrolase-like	Cytoplasm	enzyme
SFTA3	5.42E-03	2.815	surfactant associated 3	unknown	other
SFTPD	3.50E-02	−3.214	surfactant protein D	Extracellular Space	other
SFXN1	4.44E-03	1.53	sideroflexin 1	Cytoplasm	transporter
SFXN5	6.39E-03	−1.603	sideroflexin 5	Cytoplasm	transporter
SGPL1	8.85E-04	1.638	sphingosine-1-phosphate lyase 1	Cytoplasm	enzyme
SIPA1L2	3.60E-02	2.218	signal-induced proliferation-associated 1 like 2	unknown	other
SIRPG	1.71E-03	3.15	signal-regulatory protein gamma	Plasma Membrane	other
SLC13A4	1.90E-02	2.041	solute carrier family 13 (sodium/sulfate symporters), member 4	Plasma Membrane	transporter
SLC17A3	1.73E-02	1.546	solute carrier family 17 (sodium phosphate), member 3	Plasma Membrane	transporter
SLC18A2	3.45E-02	−2.131	solute carrier family 18 (vesicular monoamine), member 2	Plasma Membrane	transporter
SLC22A7	4.31E-02	3.307	solute carrier family 22 (organic anion transporter), member 7	Plasma Membrane	transporter
SLC25A18	2.60E-02	3.073	solute carrier family 25 (mitochondrial carrier), member 18	Cytoplasm	transporter
SLC26A1	4.86E-04	−2.215	solute carrier family 26 (sulfate transporter), member 1	Plasma Membrane	transporter
SLC30A6	4.27E-04	2.444	solute carrier family 30 (zinc	Cytoplasm	transporter

			transporter), member 6		
SLC35B3	8.55E-03	3.381	solute carrier family 35, member B3	Cytoplasm	other
SLC35E4	2.20E-02	−2.558	solute carrier family 35, member E4	unknown	other
SLC36A1	6.84E-03	2.788	solute carrier family 36 (proton/amino acid symporter), member 1	Plasma Membrane	transporter
SLC38A4	4.67E-02	3.156	solute carrier family 38, member 4	Plasma Membrane	transporter
SLC38A7	2.76E-02	2.361	solute carrier family 38, member 7	Plasma Membrane	transporter
SLC7A14	3.35E-02	−2.133	solute carrier family 7 (orphan transporter), member 14	unknown	other
SMAD4	3.34E-02	−1.715	SMAD family member 4	Nucleus	transcription regulator
SMC1B	1.05E-02	−2.765	structural maintenance of chromosomes 1B	Nucleus	transporter
SMCR5	6.90E-03	2.143	Smith-Magenis syndrome chromosome region, candidate 5 (non-protein coding)	unknown	other
SNORA37	9.57E-05	2.295	small nucleolar RNA, H/ACA box 37	unknown	other
SNORD123	9.28E-03	2.184	small nucleolar RNA, C/D box 123	unknown	other
SNX8	4.94E-02	3.07	sorting nexin 8	Cytoplasm	transporter
SOBP	2.12E-02	2.446	sine oculis binding protein homolog (Drosophila)	unknown	other
SOHLH2	1.38E-02	3.208	spermatogenesis and oogenesis specific basic helix-loop-helix 2	unknown	other
SP140/SP140L	4.11E-02	2.408	SP140 nuclear body protein-like	Nucleus	transcription regulator
SPATA18	2.17E-02	1.539	spermatogenesis associated 18 homolog (rat)	Cytoplasm	other
SPHK2	8.65E-03	1.852	sphingosine kinase 2	Cytoplasm	kinase
SPI1 (includes EG:20375)	1.15E-02	−2.225	spleen focus forming virus (SFFV) proviral integration oncogene spi1	Nucleus	transcription regulator
SPNS3	1.54E-02	−2.392	spinster homolog 3 (Drosophila)	unknown	other
SRP72 (includes EG:327335)	1.38E-03	4.178	signal recognition particle 72kDa	Nucleus	kinase
ST3GAL2	2.50E-02	−2.006	ST3 beta-galactoside alpha-2,3-sialyltransferase 2	Cytoplasm	enzyme
STK24	3.61E-02	−3.011	serine/threonine kinase 24	Cytoplasm	kinase
STK32B	9.58E-03	−2.425	serine/threonine kinase 32B	unknown	kinase
SUN1	4.99E-04	1.727	Sad1 and UNC84 domain	Nucleus	other

			containing 1		
SYNDIG1L	6.54E-03	2.252	synapse differentiation inducing 1-like	Cytoplasm	other
TAS2R19	7.87E-05	4.796	taste receptor, type 2, member 19	unknown	other
TBC1D26/TBC1D28	4.04E-02	2.71	TBC1 domain family, member 26	unknown	other
TET2	3.81E-02	−2.273	tet methylcytosine dioxygenase 2	unknown	other
TEX13A	9.70E-04	−2.783	testis expressed 13A	unknown	other
TF	1.77E-02	−2.479	transferrin	Extracellular Space	transporter
TGFB3	1.11E-02	−2.308	transforming growth factor, beta 3	Extracellular Space	growth factor
THPO	1.08E-02	3.198	thrombopoietin	Extracellular Space	cytokine
TIAL1	3.58E-02	−1.951	TIA1 cytotoxic granule-associated RNA binding protein-like 1	Nucleus	transcription regulator
TLR4	4.67E-02	2.186	toll-like receptor 4	Plasma Membrane	transmembrane receptor
TLR10	3.08E-04	3.194	toll-like receptor 10	Plasma Membrane	transmembrane receptor
TMC6	2.89E-04	1.635	transmembrane channel-like 6	Cytoplasm	transporter
TMEM75	2.70E-02	2.555	transmembrane protein 75	unknown	other
TMEM219	4.83E-02	1.807	transmembrane protein 219	Cytoplasm	other
TMEM229B	4.06E-02	2.064	transmembrane protein 229B	unknown	other
TMEM59L	2.47E-02	−2.651	transmembrane protein 59-like	Cytoplasm	other
TNFRSF9	9.18E-03	−2.594	tumor necrosis factor receptor superfamily, member 9	Plasma Membrane	other
TNFRSF18	3.15E-03	2.35	tumor necrosis factor receptor superfamily, member 18	Plasma Membrane	transmembrane receptor
TNFSF13	2.62E-03	2.709	tumor necrosis factor (ligand) superfamily, member 13	Extracellular Space	cytokine
TNRC6C	4.70E-02	−2.206	trinucleotide repeat containing 6C	unknown	other
TPMT	3.19E-02	1.967	thiopurine S-methyltransferase	Cytoplasm	enzyme
TPSAB1/TPSB2	3.95E-03	−2.292	tryptase alpha/beta 1	Extracellular Space	peptidase
TREML4	4.01E-02	2.546	triggering receptor expressed on myeloid cells-like 4	unknown	other
TRERF1	4.46E-03	−2.611	transcriptional regulating factor 1	Nucleus	transcription regulator
TRIM2	3.48E-02	−1.861	tripartite motif containing 2	Cytoplasm	enzyme
TRIM50	6.07E-03	−1.991	tripartite motif containing 50	unknown	other
TRIM59	3.43E-03	2.507	tripartite motif containing 59	unknown	other
TTC38	6.11E-03	−2.272	tetratricopeptide repeat domain 38	unknown	other
TTLL4	4.02E-03	2.93	tubulin tyrosine ligase-like	unknown	enzyme

			family, member 4		
TTLL10	2.91E-02	1.703	tubulin tyrosine ligase-like family, member 10	Extracellular Space	other
U2SURP	1.42E-02	1.561	U2 snRNP-associated SURP domain containing	Nucleus	other
UBC	4.89E-02	−1.655	ubiquitin C	Cytoplasm	enzyme
UBE2U	1.20E-03	2.805	ubiquitin-conjugating enzyme E2U (putative)	unknown	enzyme
UBE2W	4.36E-02	1.722	ubiquitin-conjugating enzyme E2W (putative)	unknown	enzyme
UMPS	1.64E-03	2.575	uridine monophosphate synthetase	Cytoplasm	enzyme
USP33	3.62E-02	2.675	ubiquitin specific peptidase 33	Cytoplasm	peptidase
VAX2	4.63E-03	2.545	ventral anterior homeobox 2	Nucleus	transcription regulator
VHL	8.84E-03	−1.967	von Hippel-Lindau tumor suppressor	Nucleus	other
WDR17	2.25E-02	3.426	WD repeat domain 17	unknown	other
WFIKKN1	3.73E-02	−1.722	WAP, follistatin/kazal, immunoglobulin, kunitz and netrin domain containing 1	Cytoplasm	other
WT1-AS	2.73E-02	−1.658	WT1 antisense RNA (non-protein coding)	unknown	other
YBX2	2.13E-02	−2.076	Y box binding protein 2	Cytoplasm	translation regulator
ZBTB45	4.85E-03	−2.225	zinc finger and BTB domain containing 45	Nucleus	other
ZDHHC24	1.70E-03	1.621	zinc finger, DHHC-type containing 24	Plasma Membrane	other
ZEB1	4.49E-02	3.104	zinc finger E-box binding homeobox 1	Nucleus	transcription regulator
ZFAND4	2.10E-02	1.999	zinc finger, AN1-type domain 4	unknown	other
ZFC3H1	2.43E-02	−2.93	zinc finger, C3H1-type containing	unknown	other
ZIC3	3.81E-02	−2.453	Zic family member 3	Nucleus	transcription regulator
ZNF24	2.14E-02	−1.935	zinc finger protein 24	Nucleus	transcription regulator
ZNF208	1.00E-02	−4.249	zinc finger protein 208	Nucleus	other
ZNF474	1.06E-04	2.929	zinc finger protein 474	unknown	other
ZNF496	3.75E-02	1.837	zinc finger protein 496	Nucleus	transcription regulator
ZNF549	4.99E-04	−3.112	zinc finger protein 549	unknown	other
ZNF568	4.12E-02	−3.213	zinc finger protein 568	unknown	other
ZNF573	3.17E-02	−1.787	zinc finger protein 573	Nucleus	other
ZNF578	1.38E-02	1.871	zinc finger protein 578	Nucleus	other

ZNF704	2.05E-02	−1.654	zinc finger protein 704	unknown	other
ZNF321P	2.78E-02	3.857	zinc finger protein 321, pseudogene	unknown	other
ZSCAN10	4.27E-02	−2.417	zinc finger and SCAN domain containing 10	Nucleus	transcription regulator

Supplementary Table 2. 1066 genes are unique to 4 h post-heat timepoint. The name, p-value, fold change, location and family of each gene are indicated. Genes were filtered for an absolute value log2 ration ≥1.5 and a significance value of p ≤ 0.05.

**Symbol**	**p-value**	**Log Ratio**	**Gene Name**	**Location**	**Family**
AAAS	1.01E-02	1.777	achalasia, adrenocortical insufficiency, alacrimia	Nucleus	other
AAK1	2.55E-02	2.451	AP2 associated kinase 1	Cytoplasm	kinase
ABCA3	1.23E-02	2.503	ATP-binding cassette, sub-family A (ABC1), member 3	Plasma Membrane	transporter
ABCB4	4.41E-02	2.817	ATP-binding cassette, sub-family B (MDR/TAP), member 4	Plasma Membrane	transporter
ABHD10	3.69E-04	1.789	abhydrolase domain containing 10	Cytoplasm	other
ABRA	2.58E-03	2.516	actin-binding Rho activating protein	Cytoplasm	transcription regulator
ACE	5.38E-03	−1.871	angiotensin I converting enzyme (peptidyl-dipeptidase A) 1	Plasma Membrane	peptidase
ACSL6	3.45E-02	2.464	acyl-CoA synthetase long-chain family member 6	Cytoplasm	enzyme
ACSS1	4.12E-02	−1.918	acyl-CoA synthetase short-chain family member 1	Cytoplasm	enzyme
ACSS3	2.88E-02	2.363	acyl-CoA synthetase short-chain family member 3	Cytoplasm	enzyme
ACTA1	7.98E-05	2.885	actin, alpha 1, skeletal muscle	Cytoplasm	other
ACTL7B	8.49E-03	−1.747	actin-like 7B	Cytoplasm	other
ACTN2	1.19E-02	3.966	actinin, alpha 2	Nucleus	transcription regulator
ACVR1B	6.55E-03	1.642	activin A receptor, type IB	Plasma Membrane	kinase
ACVR1C	9.50E-06	−1.942	activin A receptor, type IC	Plasma Membrane	kinase
ADAMTS6	4.19E-05	3.254	ADAM metallopeptidase with thrombospondin type 1 motif, 6	Extracellular Space	peptidase
ADAMTSL1	2.29E-02	2.42	ADAMTS-like 1	Extracellular Space	other
ADCK1	1.68E-03	2.053	aarF domain containing kinase 1	Cytoplasm	kinase
ADRA1B	3.73E-02	−3.188	adrenergic, alpha-1B-, receptor	Plasma Membrane	G-protein coupled receptor
AGXT2	2.09E-02	2.773	alanine--glyoxylate	Cytoplasm	enzyme

			aminotransferase 2		
AHCTF1	2.10E-02	2.732	AT hook containing transcription factor 1	Nucleus	transcription regulator
AHSP	3.47E-02	−2.578	alpha hemoglobin stabilizing protein	Cytoplasm	other
AJAP1	1.31E-03	−3.634	adherens junctions associated protein 1	Plasma Membrane	other
AKAP8L	7.22E-03	1.563	A kinase (PRKA) anchor protein 8-like	Nucleus	other
ALDH1A3	8.86E-06	−2.52	aldehyde dehydrogenase 1 family, member A3	Cytoplasm	enzyme
ALLC	3.01E-02	4.056	allantoicase	unknown	enzyme
ALOXE3	8.04E-04	1.505	arachidonate lipoxygenase 3	unknown	enzyme
ALS2CL	2.22E-02	1.66	ALS2 C-terminal like	Cytoplasm	other
AMIGO2	3.74E-07	1.881	adhesion molecule with Ig-like domain 2	Plasma Membrane	other
ANKH	1.27E-02	1.846	ankylosis, progressive homolog (mouse)	Plasma Membrane	transporter
ANKRD18A	6.96E-07	4.75	ankyrin repeat domain 18A	unknown	other
ANKS6	2.86E-02	2.494	ankyrin repeat and sterile alpha motif domain containing 6	Cytoplasm	other
AP1AR	3.98E-04	2.137	adaptor-related protein complex 1 associated regulatory protein	Cytoplasm	other
AP4B1	2.37E-06	1.563	adaptor-related protein complex 4, beta 1 subunit	Cytoplasm	transporter
APOL2	1.19E-02	2.695	apolipoprotein L, 2	Cytoplasm	other
APOL3	4.05E-04	3.384	apolipoprotein L, 3	Cytoplasm	transporter
AQP3	1.02E-05	−1.579	aquaporin 3 (Gill blood group)	Plasma Membrane	transporter
AQP4	2.91E-02	−3.069	aquaporin 4	Plasma Membrane	transporter
ARC	6.04E-07	6.414	activity-regulated cytoskeleton-associated protein	Cytoplasm	other
ARHGAP22	1.15E-02	2.816	Rho GTPase activating protein 22	Cytoplasm	other
ARHGAP25	7.55E-03	1.55	Rho GTPase activating protein 25	Cytoplasm	other
ARIH2	3.88E-03	1.878	ariadne homolog 2 (Drosophila)	Nucleus	enzyme
ARL4A	1.07E-04	1.772	ADP-ribosylation factor-like 4A	Nucleus	enzyme
ARTN	1.84E-05	4.836	artemin	Extracellular Space	growth factor
ARVCF	2.58E-02	2.525	armadillo repeat gene deleted in velocardiofacial syndrome	Plasma Membrane	other
ARX	1.61E-02	−2.45	aristaless related homeobox	Nucleus	transcription regulator
ASB10	2.54E-02	−2.718	ankyrin repeat and SOCS box containing 10	unknown	other
ASB12	2.97E-02	1.862	ankyrin repeat and SOCS box	Nucleus	transcription

			containing 12		regulator
ASIP	3.81E-02	−1.976	agouti signaling protein	Extracellular Space	other
ATF3	6.24E-04	2.828	activating transcription factor 3	Nucleus	transcription regulator
ATP2C2	3.78E-02	1.565	ATPase, Ca++ transporting, type 2C, member 2	Cytoplasm	enzyme
ATP5J2	2.53E-02	−1.864	ATP synthase, H+ transporting, mitochondrial Fo complex, subunit F2	Cytoplasm	transporter
ATP6V0A2	7.32E-03	2.483	ATPase, H+ transporting, lysosomal V0 subunit a2	Cytoplasm	transporter
ATP6V1H	5.76E-03	1.605	ATPase, H+ transporting, lysosomal 50/57kDa, V1 subunit H	Cytoplasm	transporter
ATP8B3	5.44E-03	1.84	ATPase, aminophospholipid transporter, class I, type 8B, member 3	Cytoplasm	transporter
AVL9 (includes EG:23080)	1.56E-06	4.589	AVL9 homolog (S. cerevisiase)	unknown	other
AZI1	2.26E-02	1.998	5-azacytidine induced 1	Cytoplasm	other
B3GALT1	1.51E-02	−2.819	UDP-Gal:betaGlcNAc beta 1,3-galactosyltransferase, polypeptide 1	Cytoplasm	enzyme
B3GALT2	4.31E-02	3.864	UDP-Gal:betaGlcNAc beta 1,3-galactosyltransferase, polypeptide 2	Cytoplasm	enzyme
B3GALT5	1.70E-03	1.802	UDP-Gal:betaGlcNAc beta 1,3-galactosyltransferase, polypeptide 5	Cytoplasm	enzyme
B3GNT7	5.29E-03	−4.3	UDP-GlcNAc:betaGal beta-1,3-N-acetylglucosaminyltransferase 7	unknown	enzyme
BAHCC1	2.59E-04	−2.057	BAH domain and coiled-coil containing 1	unknown	other
BAI3	1.74E-02	−2.752	brain-specific angiogenesis inhibitor 3	Plasma Membrane	G-protein coupled receptor
BAIAP2L1	1.81E-02	−2.108	BAI1-associated protein 2-like 1	Cytoplasm	other
BARX2	3.05E-02	−2.687	BARX homeobox 2	Nucleus	transcription regulator
BASP1	7.06E-03	3.862	brain abundant, membrane attached signal protein 1	Nucleus	transcription regulator
BAZ1B	1.57E-02	−1.865	bromodomain adjacent to zinc finger domain, 1B	Nucleus	transcription regulator
BBS12	3.63E-03	−1.598	Bardet-Biedl syndrome 12	unknown	other
BCL3	1.09E-02	−2.037	B-cell CLL/lymphoma 3	Nucleus	transcription regulator

BCL2L15	3.68E-02	3.305	BCL2-like 15	unknown	other
BDNF	1.04E-02	1.525	brain-derived neurotrophic factor	Extracellular Space	growth factor
BICD1	3.02E-03	2.126	bicaudal D homolog 1 (Drosophila)	Cytoplasm	other
BMP6	3.53E-02	−1.564	bone morphogenetic protein 6	Extracellular Space	growth factor
BSN-AS2	6.08E-03	2.387	BSN antisense RNA 2 (non-protein coding)	unknown	other
BTG2	5.14E-03	−1.825	BTG family, member 2	Nucleus	transcription regulator
C2	8.77E-06	−1.645	complement component 2	Extracellular Space	peptidase
C7	2.83E-02	−1.893	complement component 7	Extracellular Space	other
C11orf57	3.25E-02	−2.567	chromosome 11 open reading frame 57	unknown	other
C11orf67	5.64E-03	3.826	chromosome 11 open reading frame 67	unknown	other
C11orf94	4.53E-02	−1.957	chromosome 11 open reading frame 94	unknown	other
C12orf51	1.06E-02	−2.119	chromosome 12 open reading frame 51	unknown	other
C13orf15	8.68E-07	1.752	chromosome 13 open reading frame 15	Cytoplasm	other
C14orf38	4.34E-05	1.563	chromosome 14 open reading frame 38	unknown	other
C14orf56	3.44E-02	2.204	chromosome 14 open reading frame 56	unknown	other
C14orf135	2.02E-02	2.873	chromosome 14 open reading frame 135	unknown	other
C15orf48	1.45E-05	−2.225	chromosome 15 open reading frame 48	Nucleus	other
C17orf70	3.66E-03	−2.12	chromosome 17 open reading frame 70	Nucleus	other
C17orf104	1.93E-03	4.104	chromosome 17 open reading frame 104	unknown	other
C18orf8	9.73E-03	−2.981	chromosome 18 open reading frame 8	unknown	other
C18orf21	3.83E-02	2.953	chromosome 18 open reading frame 21	unknown	other
C19orf12	3.60E-02	−2.173	chromosome 19 open reading frame 12	unknown	other
C19orf26	1.41E-02	−3.482	chromosome 19 open reading frame 26	unknown	other
C19orf46	1.81E-02	2.192	chromosome 19 open reading frame 46	Nucleus	other

C1orf38	1.12E-03	−1.823	chromosome 1 open reading frame 38	unknown	other
C1orf74	5.59E-09	−2.822	chromosome 1 open reading frame 74	unknown	other
C1orf106	2.13E-03	−1.687	chromosome 1 open reading frame 106	unknown	other
C1orf116	3.95E-05	−1.565	chromosome 1 open reading frame 116	unknown	other
C1orf126	7.71E-03	−3.935	chromosome 1 open reading frame 126	unknown	other
C1orf162	3.48E-02	2.677	chromosome 1 open reading frame 162	unknown	transporter
C1orf173	1.76E-02	4.44	chromosome 1 open reading frame 173	unknown	other
C1orf186	3.50E-02	−1.664	chromosome 1 open reading frame 186	unknown	other
C1orf226	1.95E-02	2.044	chromosome 1 open reading frame 226	unknown	other
C20orf181	1.99E-02	−1.645	chromosome 20 open reading frame 181	unknown	other
C21orf81	3.55E-02	2.556	ankyrin repeat domain 20 family, member A3 pseudogene	unknown	other
C22orf15	1.64E-02	−3.072	chromosome 22 open reading frame 15	unknown	other
C22orf34	2.44E-03	2.324	chromosome 22 open reading frame 34	unknown	other
C22orf43	3.62E-02	−1.506	chromosome 22 open reading frame 43	unknown	other
C2orf74	2.23E-05	1.668	chromosome 2 open reading frame 74	unknown	other
C3orf32	7.15E-03	−1.551	chromosome 3 open reading frame 32	unknown	other
C3orf33	2.62E-02	2.215	chromosome 3 open reading frame 33	unknown	other
C3orf52	6.31E-06	−1.888	chromosome 3 open reading frame 52	unknown	other
C3orf56	2.74E-02	−1.912	chromosome 3 open reading frame 56	unknown	other
C3P1	1.19E-02	2.849	complement component 3 precursor pseudogene	unknown	other
C4orf47	4.22E-02	3.271	chromosome 4 open reading frame 47	unknown	other
C5orf27	1.57E-03	4.209	chromosome 5 open reading frame 27	unknown	other
C5orf48	2.24E-02	4.266	chromosome 5 open reading frame 48	unknown	other

C5orf54	2.95E-05	4.734	chromosome 5 open reading frame 54	unknown	other
C6orf27	4.98E-02	2.034	chromosome 6 open reading frame 27	unknown	other
C6orf123	2.84E-02	−1.663	chromosome 6 open reading frame 123	unknown	other
C6orf132	7.28E-05	−1.965	chromosome 6 open reading frame 132	unknown	other
C6orf168	3.04E-03	2.846	chromosome 6 open reading frame 168	unknown	other
C7orf44	1.65E-05	2.107	chromosome 7 open reading frame 44	unknown	other
C7orf63	3.78E-02	−1.888	chromosome 7 open reading frame 63	unknown	other
C7orf71	4.25E-02	2.546	chromosome 7 open reading frame 71	unknown	other
C8orf31	1.71E-02	−2.308	chromosome 8 open reading frame 31	unknown	other
C9orf68	1.41E-02	−2.454	chromosome 9 open reading frame 68	unknown	other
C9orf79	3.24E-02	−2.401	chromosome 9 open reading frame 79	unknown	other
C9orf135	2.69E-02	−2.575	chromosome 9 open reading frame 135	unknown	other
C9orf152	4.44E-04	1.53	chromosome 9 open reading frame 152	unknown	other
CA6	3.39E-02	1.539	carbonic anhydrase VI	Extracellular Space	enzyme
CA10	4.04E-02	2.317	carbonic anhydrase X	unknown	enzyme
CACYBP	8.81E-07	1.502	calcyclin binding protein	Nucleus	other
CADM4	2.19E-02	−1.566	cell adhesion molecule 4	Plasma Membrane	other
CALB1	6.04E-03	2.687	calbindin 1, 28kDa	Cytoplasm	other
CALCOCO2	4.02E-02	−1.781	calcium binding and coiled-coil domain 2	Nucleus	other
CAMSAP1	4.62E-06	−1.64	calmodulin regulated spectrin-associated protein 1	unknown	other
CANT1	1.53E-02	−1.565	calcium activated nucleotidase 1	Extracellular Space	enzyme
CAPN3	7.82E-05	−1.783	calpain 3, (p94)	Cytoplasm	peptidase
CARD8	2.99E-02	−2.74	caspase recruitment domain family, member 8	Nucleus	other
CASD1	2.71E-02	2.408	CAS1 domain containing 1	Cytoplasm	enzyme
CASQ1	1.96E-05	4.365	calsequestrin 1 (fast-twitch, skeletal muscle)	Cytoplasm	other
CASZ1	2.96E-06	−2.448	castor zinc finger 1	Nucleus	enzyme

CBX4	1.04E-02	1.981	chromobox homolog 4	Nucleus	transcription regulator
CCBL1	2.93E-02	2.256	cysteine conjugate-beta lyase, cytoplasmic	Cytoplasm	enzyme
CCDC15	1.37E-04	1.503	coiled-coil domain containing 15	Cytoplasm	other
CCDC75	2.96E-02	2.927	coiled-coil domain containing 75	unknown	other
CCDC121	3.65E-05	3.172	coiled-coil domain containing 121	unknown	other
CCDC125	2.45E-03	2.273	coiled-coil domain containing 125	unknown	other
CCNB2	1.45E-02	2.154	cyclin B2	Cytoplasm	other
CCNL2	1.47E-02	−1.781	cyclin L2	Nucleus	other
CCZ1/CCZ1B	4.01E-02	−1.979	CCZ1 vacuolar protein trafficking and biogenesis associated homolog (S. cerevisiae)	unknown	other
CD109	2.48E-06	−1.568	CD109 molecule	Plasma Membrane	other
CD1E	9.13E-03	2.391	CD1e molecule	Cytoplasm	other
CDC45	1.61E-04	2.391	cell division cycle 45 homolog (S. cerevisiae)	Nucleus	other
CDC14A	1.60E-03	2.169	CDC14 cell division cycle 14 homolog A (S. cerevisiae)	Nucleus	phosphatase
CDC14B	5.15E-03	−3.141	CDC14 cell division cycle 14 homolog B (S. cerevisiae)	Nucleus	phosphatase
CDC25A	2.09E-02	1.774	cell division cycle 25 homolog A (S. pombe)	Nucleus	phosphatase
CDC42BPA	2.47E-02	1.935	CDC42 binding protein kinase alpha (DMPK-like)	Cytoplasm	kinase
CDC42EP1	6.80E-03	−1.616	CDC42 effector protein (Rho GTPase binding) 1	Extracellular Space	other
CDC42EP2	4.60E-03	−2.1	CDC42 effector protein (Rho GTPase binding) 2	Plasma Membrane	other
CDC42EP4	1.98E-03	−2.069	CDC42 effector protein (Rho GTPase binding) 4	Cytoplasm	other
CDK13	3.08E-03	−2.524	cyclin-dependent kinase 13	Nucleus	kinase
CDK5R1	1.55E-03	−2.053	cyclin-dependent kinase 5, regulatory subunit 1 (p35)	Nucleus	kinase
CDKN1C	2.14E-02	−1.649	cyclin-dependent kinase inhibitor 1C (p57, Kip2)	Nucleus	other
CDKN2C	1.59E-03	2.508	cyclin-dependent kinase inhibitor 2C (p18, inhibits CDK4)	Nucleus	transcription regulator
CEBPA	1.24E-03	−1.667	CCAAT/enhancer binding protein (C/EBP), alpha	Nucleus	transcription regulator
CEBPD	2.45E-03	−1.5	CCAAT/enhancer binding protein (C/EBP), delta	Nucleus	transcription regulator
CELF4	3.94E-02	3.819	CUGBP, Elav-like family member 4	Nucleus	translation regulator
CELP	2.58E-02	−1.539	carboxyl ester lipase pseudogene	unknown	other

CELSR2	3.99E-06	−1.573	cadherin, EGF LAG seven-pass G-type receptor 2 (flamingo homolog, Drosophila)	Plasma Membrane	G-protein coupled receptor
CENPJ	4.17E-02	1.799	centromere protein J	Nucleus	transcription regulator
CEP95	2.77E-02	−2.419	centrosomal protein 95kDa	unknown	other
CEP120	4.26E-03	1.713	centrosomal protein 120kDa	Cytoplasm	other
CEP170	4.13E-04	2.354	centrosomal protein 170kDa	Nucleus	other
CFLAR	2.24E-03	−1.837	CASP8 and FADD-like apoptosis regulator	Cytoplasm	other
CFTR	2.05E-02	−3.743	cystic fibrosis transmembrane conductance regulator (ATP-binding cassette sub-family C, member 7)	Plasma Membrane	ion channel
CHAC1	3.40E-03	1.729	ChaC, cation transport regulator homolog 1 (E. coli)	Cytoplasm	other
CHRDL2	2.84E-03	−1.584	chordin-like 2	Extracellular Space	other
CHRNA10	2.76E-02	3.047	cholinergic receptor, nicotinic, alpha 10	Plasma Membrane	transmembrane receptor
CHRNG	7.86E-03	2.283	cholinergic receptor, nicotinic, gamma	Plasma Membrane	transmembrane receptor
CLDN1	8.44E-06	−1.946	claudin 1	Plasma Membrane	other
CLDN3	3.30E-02	−2.522	claudin 3	Plasma Membrane	transmembrane receptor
CLDN4	3.61E-04	−3.88	claudin 4	Plasma Membrane	transmembrane receptor
CLDN7	2.38E-03	−1.523	claudin 7	Plasma Membrane	other
CLDN18	4.81E-03	−2.685	claudin 18	Plasma Membrane	other
CLDN23	3.39E-02	−2.655	claudin 23	Plasma Membrane	other
CLEC4M	1.37E-03	1.999	C-type lectin domain family 4, member M	Plasma Membrane	other
CLIC5	4.40E-02	−3.698	chloride intracellular channel 5	Cytoplasm	ion channel
CLNK	1.89E-02	2.741	cytokine-dependent hematopoietic cell linker	Cytoplasm	other
CLVS2	1.94E-02	−2.045	clavesin 2	Cytoplasm	transporter
CLYBL	4.90E-02	2.085	citrate lyase beta like	Cytoplasm	enzyme
CNBD1	1.86E-02	2.405	cyclic nucleotide binding domain containing 1	unknown	other
CNDP2	6.07E-03	2.807	CNDP dipeptidase 2 (metallopeptidase M20 family)	Cytoplasm	peptidase
CNKSR2	1.98E-02	−2.627	connector enhancer of kinase	Plasma	other

			suppressor of Ras 2	Membrane	
CNNM2	9.65E-03	2.025	cyclin M2	unknown	other
CNOT2	9.90E-04	2.575	CCR4-NOT transcription complex, subunit 2	Nucleus	transcription regulator
CNOT4	2.41E-04	2.991	CCR4-NOT transcription complex, subunit 4	Cytoplasm	enzyme
COL10A1	2.20E-02	4.511	collagen, type X, alpha 1	Extracellular Space	other
COL1A2	3.99E-02	2.375	collagen, type I, alpha 2	Extracellular Space	other
COL28A1	2.45E-02	2.939	collagen, type XXVIII, alpha 1	Extracellular Space	other
COL6A5	4.16E-02	−2.56	collagen, type VI, alpha 5	Extracellular Space	other
COLEC12	3.33E-02	−1.513	collectin sub-family member 12	Plasma Membrane	transmembrane receptor
COMP	2.71E-02	3.626	cartilage oligomeric matrix protein	Extracellular Space	other
COPG2	2.77E-02	2.181	coatomer protein complex, subunit gamma 2	Cytoplasm	transporter
COQ10B	8.36E-03	−1.829	coenzyme Q10 homolog B (S. cerevisiae)	Cytoplasm	other
COX5A (includes EG:12858)	4.75E-02	2.324	cytochrome c oxidase subunit Va	Cytoplasm	enzyme
CPEB2	7.16E-04	−2.074	cytoplasmic polyadenylation element binding protein 2	Cytoplasm	other
CPEB3	3.51E-03	1.712	cytoplasmic polyadenylation element binding protein 3	unknown	other
CRYAB	5.88E-06	2.903	crystallin, alpha B	Nucleus	other
CSGALNACT2	1.85E-02	1.653	chondroitin sulfate N-acetylgalactosaminyltransferase 2	Cytoplasm	enzyme
CSMD3	1.65E-03	2.129	CUB and Sushi multiple domains 3	unknown	enzyme
CSN1S1	4.16E-02	1.775	casein alpha s1	Extracellular Space	other
CSPG4P5	1.69E-02	−2.111	chondroitin sulfate proteoglycan 4 pseudogene 5	unknown	other
CST5	1.96E-03	3.864	cystatin D	Cytoplasm	other
CT47A1 (includes others)	1.85E-02	−1.788	cancer/testis antigen family 47, member A10	unknown	other
CTDP1	1.43E-02	−2.082	CTD (carboxy-terminal domain, RNA polymerase II, polypeptide A) phosphatase, subunit 1	Nucleus	phosphatase
CTGF	4.74E-07	3.128	connective tissue growth factor	Extracellular Space	growth factor

CTH	3.37E-05	1.515	cystathionase (cystathionine gamma-lyase)	Cytoplasm	enzyme
CTNNA3	3.08E-02	−2.233	catenin (cadherin-associated protein), alpha 3	Plasma Membrane	other
CTRB1	4.99E-02	−2.284	chymotrypsinogen B1	Extracellular Space	peptidase
CTSC	5.13E-05	−1.694	cathepsin C	Cytoplasm	peptidase
CTTNBP2NL	2.75E-04	−2.148	CTTNBP2 N-terminal like	Cytoplasm	other
CUL3 (includes EG:26554)	5.00E-03	2.926	cullin 3	Nucleus	enzyme
CXCR1	2.25E-02	1.552	chemokine (C-X-C motif) receptor 1	Plasma Membrane	G-protein coupled receptor
CXorf21	8.95E-03	2.986	chromosome X open reading frame 21	unknown	enzyme
CYB5B	1.20E-03	1.656	cytochrome b5 type B (outer mitochondrial membrane)	Cytoplasm	enzyme
CYB5RL	4.35E-02	−2.032	cytochrome b5 reductase-like	unknown	other
CYP1A1	5.82E-03	−2.317	cytochrome P450, family 1, subfamily A, polypeptide 1	Cytoplasm	enzyme
CYP2U1	1.23E-02	1.548	cytochrome P450, family 2, subfamily U, polypeptide 1	Cytoplasm	enzyme
CYR61	1.01E-03	1.504	cysteine-rich, angiogenic inducer, 61	Extracellular Space	other
CYS1	2.57E-02	2.1	cystin 1	Cytoplasm	other
CYSLTR1	1.14E-03	2.072	cysteinyl leukotriene receptor 1	Plasma Membrane	G-protein coupled receptor
CYTH2	7.80E-03	−2.542	cytohesin 2	Cytoplasm	other
D21S2088E	5.05E-03	3.224	D21S2088E	unknown	other
DACH1	1.92E-02	2.956	dachshund homolog 1 (Drosophila)	Nucleus	transcription regulator
DAPP1	3.80E-05	−1.763	dual adaptor of phosphotyrosine and 3-phosphoinositides	Cytoplasm	other
DAZL	1.62E-02	3.65	deleted in azoospermia-like	Cytoplasm	translation regulator
DCAF15	4.49E-02	−1.551	DDB1 and CUL4 associated factor 15	unknown	other
DCAF12L2	4.97E-02	2.365	DDB1 and CUL4 associated factor 12-like 2	unknown	other
DCLRE1B	2.47E-04	1.551	DNA cross-link repair 1B	Nucleus	enzyme
DCLRE1C	3.91E-03	−4.163	DNA cross-link repair 1C	Nucleus	enzyme
DCUN1D1	1.87E-04	−1.731	DCN1, defective in cullin neddylation 1, domain containing 1 (S. cerevisiae)	unknown	other
DCUN1D3	2.83E-06	−2.337	DCN1, defective in cullin	Cytoplasm	other

			neddylation 1, domain containing 3 (S. cerevisiae)		
DDAH2	2.50E-02	−1.836	dimethylarginine dimethylaminohydrolase 2	unknown	enzyme
DDX25	4.44E-02	2.116	DEAD (Asp-Glu-Ala-Asp) box polypeptide 25	Nucleus	enzyme
DDX50	1.76E-02	−1.615	DEAD (Asp-Glu-Ala-Asp) box polypeptide 50	Nucleus	enzyme
DDX59	1.98E-03	1.659	DEAD (Asp-Glu-Ala-Asp) box polypeptide 59	unknown	enzyme
DDX19A	1.04E-03	2.781	DEAD (Asp-Glu-Ala-As) box polypeptide 19A	Nucleus	enzyme
DECR2	3.96E-02	3.023	2,4-dienoyl CoA reductase 2, peroxisomal	Cytoplasm	enzyme
DEFB105A/DEFB105B	1.29E-02	3.206	defensin, beta 105A	Extracellular Space	other
DENND2C	1.46E-02	−1.678	DENN/MADD domain containing 2C	unknown	other
DEPDC7	9.20E-06	1.936	DEP domain containing 7	Cytoplasm	other
DERL1	7.02E-03	1.921	Der1-like domain family, member 1	Cytoplasm	other
DGKD	4.71E-02	1.941	diacylglycerol kinase, delta 130kDa	Cytoplasm	kinase
DHRS3	4.54E-05	−1.861	dehydrogenase/reductase (SDR family) member 3	Cytoplasm	enzyme
DHX57	3.66E-04	1.747	DEAH (Asp-Glu-Ala-Asp/His) box polypeptide 57	unknown	other
DIAPH2	1.10E-02	2.793	diaphanous homolog 2 (Drosophila)	Cytoplasm	other
DIEXF	8.35E-05	−1.986	digestive organ expansion factor homolog (zebrafish)	Nucleus	other
DIP2C	2.05E-03	2.393	DIP2 disco-interacting protein 2 homolog C (Drosophila)	unknown	other
DKFZP547J0410	1.75E-02	2.026	DKFZP547J0410 protein	unknown	other
DKK3	1.35E-03	2.781	dickkopf 3 homolog (Xenopus laevis)	Extracellular Space	cytokine
DLG3	8.53E-03	1.91	discs, large homolog 3 (Drosophila)	Plasma Membrane	kinase
DLGAP2	4.46E-03	2.399	discs, large (Drosophila) homolog-associated protein 2	Plasma Membrane	other
DLL1	7.51E-07	2.605	delta-like 1 (Drosophila)	Plasma Membrane	enzyme
DLX6-AS1	1.75E-02	1.808	DLX6 antisense RNA 1 (non-protein coding)	unknown	other
DNAH7	2.11E-03	4.217	dynein, axonemal, heavy chain 7	Cytoplasm	other

DNAJA1	9.48E-06	1.696	DnaJ (Hsp40) homolog, subfamily A, member 1	Nucleus	other
DNAJA4	3.29E-06	2.39	DnaJ (Hsp40) homolog, subfamily A, member 4	Nucleus	other
DNAJB1	3.91E-05	1.68	DnaJ (Hsp40) homolog, subfamily B, member 1	Nucleus	other
DNAJC2	5.33E-03	−3.205	DnaJ (Hsp40) homolog, subfamily C, member 2	Nucleus	other
DNASE1L3	3.28E-02	2.418	deoxyribonuclease I-like 3	Nucleus	enzyme
DNMT3B	4.83E-04	2.71	DNA (cytosine-5-)-methyltransferase 3 beta	Nucleus	enzyme
DPY19L1	2.26E-03	−2.155	dpy-19-like 1 (C. elegans)	unknown	other
DRD3	2.45E-02	1.633	dopamine receptor D3	Plasma Membrane	G-protein coupled receptor
DSC2	1.01E-05	−1.534	desmocollin 2	Plasma Membrane	other
DSG3	4.70E-06	−1.505	desmoglein 3	Plasma Membrane	other
DTX3	2.38E-02	−2.641	deltex homolog 3 (Drosophila)	Cytoplasm	other
DUSP2	4.42E-03	1.68	dual specificity phosphatase 2	Nucleus	phosphatase
DUSP4	1.40E-05	2.02	dual specificity phosphatase 4	Nucleus	phosphatase
DUSP18	3.47E-04	−1.737	dual specificity phosphatase 18	Cytoplasm	phosphatase
DYNC2H1	3.65E-02	3.609	dynein, cytoplasmic 2, heavy chain 1	Cytoplasm	other
DYNLRB1	3.36E-02	2.286	dynein, light chain, roadblock-type 1	Cytoplasm	other
E2F2	3.55E-02	−2.335	E2F transcription factor 2	Nucleus	transcription regulator
E2F8	2.78E-04	−2.491	E2F transcription factor 8	Nucleus	other
ECE1	1.67E-02	−1.562	endothelin converting enzyme 1	Plasma Membrane	peptidase
EDNRA	1.44E-02	3.377	endothelin receptor type A	Plasma Membrane	transmembrane receptor
EFNA1	1.72E-04	−1.517	ephrin-A1	Plasma Membrane	other
EFS	2.25E-03	1.632	embryonal Fyn-associated substrate	Cytoplasm	other
EGF (includes EG:13645)	1.84E-02	2.292	epidermal growth factor	Extracellular Space	growth factor
EGFL7	3.00E-02	2.497	EGF-like-domain, multiple 7	Extracellular Space	other
EGR1	1.50E-02	1.732	early growth response 1	Nucleus	transcription regulator
EID2B	2.21E-04	1.803	EP300 interacting inhibitor of differentiation 2B	unknown	other
EIF3M	3.59E-02	−3.93	eukaryotic translation initiation	unknown	other

			factor 3, subunit M		
ELAC1	6.77E-05	1.585	elaC homolog 1 (E. coli)	Nucleus	enzyme
ELF1	2.45E-04	−1.911	E74-like factor 1 (ets domain transcription factor)	Nucleus	transcription regulator
ELF3	1.13E-02	−2.398	E74-like factor 3 (ets domain transcription factor, epithelial-specific)	Nucleus	transcription regulator
ELF4	8.16E-04	−1.542	E74-like factor 4 (ets domain transcription factor)	Nucleus	transcription regulator
ELK3	2.73E-07	−1.904	ELK3, ETS-domain protein (SRF accessory protein 2)	Nucleus	transcription regulator
ELN	5.77E-03	2.788	elastin	Extracellular Space	other
ELOVL5	2.70E-04	1.984	ELOVL fatty acid elongase 5	Cytoplasm	enzyme
EMX2	8.72E-03	1.634	empty spiracles homeobox 2	Nucleus	transcription regulator
ENOSF1	5.49E-03	2.863	enolase superfamily member 1	unknown	other
ENOX2	9.93E-03	−2.549	ecto-NOX disulfide-thiol exchanger 2	Plasma Membrane	enzyme
ENPP5	4.26E-02	−2.697	ectonucleotide pyrophosphatase/phosphodiesterase 5 (putative)	Extracellular Space	enzyme
ENTPD3	1.87E-04	−2.366	ectonucleoside triphosphate diphosphohydrolase 3	Plasma Membrane	enzyme
EPAS1	4.63E-02	2.495	endothelial PAS domain protein 1	Nucleus	transcription regulator
EPB41L5	9.20E-05	−1.508	erythrocyte membrane protein band 4.1 like 5	Plasma Membrane	other
EPHB3	1.29E-04	−1.989	EPH receptor B3	Plasma Membrane	kinase
EPN3	4.66E-05	3.75	epsin 3	Cytoplasm	other
EPO	6.55E-04	2.45	erythropoietin	Extracellular Space	cytokine
EPSTI1	3.44E-02	1.703	epithelial stromal interaction 1 (breast)	unknown	other
ERBB2	5.04E-03	1.756	v-erb-b2 erythroblastic leukemia viral oncogene homolog 2, neuro/glioblastoma derived oncogene homolog (avian)	Plasma Membrane	kinase
ERBB3	1.48E-05	−2.432	v-erb-b2 erythroblastic leukemia viral oncogene homolog 3 (avian)	Plasma Membrane	kinase
ERC2	4.03E-04	3.315	ELKS/RAB6-interacting/CAST family member 2	Cytoplasm	other
ERN2	3.81E-02	2.087	endoplasmic reticulum to nucleus signaling 2	Cytoplasm	kinase
ETV7	1.06E-06	6.123	ets variant 7	Nucleus	transcription

					regulator
EVI2A	1.07E-02	2.019	ecotropic viral integration site 2A	Plasma Membrane	transmembrane receptor
F2RL1	7.46E-04	−2.57	coagulation factor II (thrombin) receptor-like 1	Plasma Membrane	G-protein coupled receptor
FABP7	1.90E-02	−3.083	fatty acid binding protein 7, brain	Cytoplasm	transporter
FAM100A	3.24E-02	−2.2	family with sequence similarity 100, member A	unknown	other
FAM101A	5.47E-03	1.937	family with sequence similarity 101, member A	unknown	transporter
FAM113B	9.84E-05	1.745	family with sequence similarity 113, member B	unknown	other
FAM120B	3.29E-05	2.409	family with sequence similarity 120B	unknown	other
FAM122C	1.38E-03	1.611	family with sequence similarity 122C	unknown	other
FAM123A	2.67E-02	3.514	family with sequence similarity 123A	unknown	other
FAM13A-AS1	1.40E-03	1.644	FAM13A antisense RNA 1 (non-protein coding)	unknown	other
FAM161A	2.66E-03	1.549	family with sequence similarity 161, member A	Cytoplasm	other
FAM166A	1.79E-03	1.624	family with sequence similarity 166, member A	unknown	other
FAM178A	4.56E-02	−1.652	family with sequence similarity 178, member A	unknown	other
FAM182A	1.21E-02	2.513	family with sequence similarity 182, member A	unknown	other
FAM201A	2.42E-02	1.754	family with sequence similarity 201, member A	unknown	other
FAM212B	5.66E-03	−2.828	family with sequence similarity 212, member B	unknown	other
FAM27A	3.32E-02	2.259	family with sequence similarity 27, member A	unknown	other
FAM3A	4.37E-04	2.697	family with sequence similarity 3, member A	Extracellular Space	other
FAM3B	3.52E-03	2.281	family with sequence similarity 3, member B	Extracellular Space	cytokine
FAM46C	2.94E-04	1.961	family with sequence similarity 46, member C	Extracellular Space	other
FAM49A	3.85E-02	2.815	family with sequence similarity 49, member A	unknown	other
FAM59A	1.14E-05	−2.057	family with sequence similarity 59, member A	unknown	other
FAM65C	2.09E-04	−3.222	family with sequence similarity 65,	unknown	other

			member C		
FAM66C	4.04E-02	−1.7	family with sequence similarity 66, member C	unknown	other
FAM81A	3.43E-03	3.377	family with sequence similarity 81, member A	unknown	other
FAM82A1	2.11E-03	2.762	family with sequence similarity 82, member A1	Cytoplasm	other
FAM86C1	4.84E-02	2.624	family with sequence similarity 86, member C1	unknown	other
FANCB	1.16E-04	3.218	Fanconi anemia, complementation group B	Nucleus	other
FANCC	4.62E-02	2.237	Fanconi anemia, complementation group C	Nucleus	other
FAR2	1.35E-02	3.601	fatty acyl CoA reductase 2	Cytoplasm	enzyme
FARP1	5.29E-03	−2.457	FERM, RhoGEF (ARHGEF) and pleckstrin domain protein 1 (chondrocyte-derived)	unknown	other
FAT3	9.56E-04	2.655	FAT tumor suppressor homolog 3 (Drosophila)	unknown	other
FBN1	3.63E-04	3.536	fibrillin 1	Extracellular Space	other
FBXL14	2.73E-06	1.526	F-box and leucine-rich repeat protein 14	Cytoplasm	enzyme
FBXL20	3.99E-02	−2.276	F-box and leucine-rich repeat protein 20	Cytoplasm	other
FBXO3	4.03E-04	1.946	F-box protein 3	unknown	enzyme
FBXO11	2.36E-03	4.588	F-box protein 11	Cytoplasm	enzyme
FBXO17	9.24E-03	−1.529	F-box protein 17	unknown	other
FBXO32	2.08E-02	−2.875	F-box protein 32	Cytoplasm	enzyme
FBXW10	1.37E-03	1.776	F-box and WD repeat domain containing 10	unknown	other
FCAR	3.81E-02	2.104	Fc fragment of IgA, receptor for	Plasma Membrane	other
FCER1G	3.02E-02	4.01	Fc fragment of IgE, high affinity I, receptor for; gamma polypeptide	Plasma Membrane	transmembrane receptor
FCHSD1	1.99E-02	−1.565	FCH and double SH3 domains 1	unknown	other
FGD2	3.14E-02	1.727	FYVE, RhoGEF and PH domain containing 2	Cytoplasm	other
FGF12	3.37E-02	2.879	fibroblast growth factor 12	Extracellular Space	growth factor
FGF19	9.23E-04	−3.574	fibroblast growth factor 19	Extracellular Space	growth factor
FIGN	4.54E-04	1.524	fidgetin	Nucleus	other
FILIP1L	7.90E-06	1.959	filamin A interacting protein 1-like	Nucleus	other
FKBP4	7.70E-03	1.831	FK506 binding protein 4, 59kDa	Nucleus	enzyme
FLJ31356	3.83E-02	3.162	uncharacterized protein FLJ31356	unknown	other

FLJ37453	1.88E-02	1.942	uncharacterized LOC729614	unknown	other
FLJ39051	8.87E-04	1.778	uncharacterized LOC399972	unknown	other
FLJ40852	6.31E-03	−2.266	uncharacterized LOC285962	unknown	other
FLJ46875	8.74E-03	1.54	uncharacterized LOC440918	unknown	other
FLT1	2.10E-04	−4.153	fms-related tyrosine kinase 1 (vascular endothelial growth factor/vascular permeability factor receptor)	Plasma Membrane	kinase
FMN1 (includes EG:296512)	6.96E-03	−2.218	formin 1	Plasma Membrane	other
FNIP1	1.82E-03	1.65	folliculin interacting protein 1	Cytoplasm	other
FOS	1.79E-08	2.833	FBJ murine osteosarcoma viral oncogene homolog	Nucleus	transcription regulator
FOSB	7.43E-06	2.127	FBJ murine osteosarcoma viral oncogene homolog B	Nucleus	transcription regulator
FRAS1	2.35E-02	−4.214	Fraser syndrome 1	Extracellular Space	other
FRMD8	1.76E-06	−2.081	FERM domain containing 8	unknown	other
FRY	7.34E-04	1.585	furry homolog (Drosophila)	unknown	other
FTCD	3.58E-02	1.819	formiminotransferase cyclodeaminase	Cytoplasm	enzyme
FXR2	4.55E-03	4.187	fragile X mental retardation, autosomal homolog 2	Cytoplasm	other
FYB	2.03E-03	−1.665	FYN binding protein	Nucleus	other
FZD7	4.02E-04	−1.641	frizzled family receptor 7	Plasma Membrane	G-protein coupled receptor
GABRB1	1.26E-03	2.425	gamma-aminobutyric acid (GABA) A receptor, beta 1	Plasma Membrane	ion channel
GADD45G	3.97E-03	3.802	growth arrest and DNA-damage-inducible, gamma	Nucleus	other
GALNT5	4.85E-03	−1.821	UDP-N-acetyl-alpha-D-galactosamine:polypeptide N-acetylgalactosaminyltransferase 5 (GalNAc-T5)	Cytoplasm	enzyme
GATA1	3.69E-02	2.488	GATA binding protein 1 (globin transcription factor 1)	Nucleus	transcription regulator
GATA6	1.54E-03	−2.36	GATA binding protein 6	Nucleus	transcription regulator
GCET2	3.34E-02	2.328	germinal center expressed transcript 2	Cytoplasm	other
GDF15	7.42E-06	2.072	growth differentiation factor 15	Extracellular Space	growth factor
GHRH	1.08E-02	2.518	growth hormone releasing hormone	Extracellular Space	other

GKAP1	3.41E-03	1.518	G kinase anchoring protein 1	Cytoplasm	other
GLYATL1	4.36E-02	2.359	glycine-N-acyltransferase-like 1	unknown	other
GMFG	1.03E-02	2.562	glia maturation factor, gamma	Cytoplasm	growth factor
GNB1L	1.94E-04	3.259	guanine nucleotide binding protein (G protein), beta polypeptide 1-like	Cytoplasm	other
GNRHR	4.70E-02	2.775	gonadotropin-releasing hormone receptor	Plasma Membrane	G-protein coupled receptor
GP1BB	2.67E-02	−1.516	glycoprotein Ib (platelet), beta polypeptide	Plasma Membrane	other
GPR39	2.75E-02	2.385	G protein-coupled receptor 39	Plasma Membrane	G-protein coupled receptor
GPR98	3.41E-03	−1.649	G protein-coupled receptor 98	Plasma Membrane	G-protein coupled receptor
GPR115	5.41E-04	−1.689	G protein-coupled receptor 115	Plasma Membrane	G-protein coupled receptor
GPR126	2.05E-02	1.69	G protein-coupled receptor 126	Plasma Membrane	G-protein coupled receptor
GPR182	4.73E-03	1.921	G protein-coupled receptor 182	Plasma Membrane	G-protein coupled receptor
GPRC5B	1.95E-02	−2.336	G protein-coupled receptor, family C, group 5, member B	Plasma Membrane	G-protein coupled receptor
GPX5	3.79E-02	−2.352	glutathione peroxidase 5 (epididymal androgen-related protein)	Extracellular Space	enzyme
GPX8	4.06E-04	1.532	glutathione peroxidase 8 (putative)	unknown	enzyme
GRAMD2	6.95E-04	1.586	GRAM domain containing 2	unknown	other
GRAMD3	2.02E-04	−1.679	GRAM domain containing 3	unknown	other
GRAP	4.97E-02	−1.951	GRB2-related adaptor protein	Cytoplasm	other
GRB7	8.67E-03	−1.519	growth factor receptor-bound protein 7	Plasma Membrane	other
GRHL3	1.08E-07	−3.315	grainyhead-like 3 (Drosophila)	Nucleus	other
GRIA3	2.99E-02	2.835	glutamate receptor, ionotrophic, AMPA 3	Plasma Membrane	ion channel
GRIN2A	2.52E-04	3.731	glutamate receptor, ionotropic, N-methyl D-aspartate 2A	Plasma Membrane	ion channel
GSC2	3.62E-02	−2.488	goosecoid homeobox 2	Nucleus	transcription regulator
GTF2I	9.17E-04	1.664	general transcription factor IIi	Nucleus	transcription regulator

GUCA2A	6.21E-03	−2.126	guanylate cyclase activator 2A (guanylin)	Extracellular Space	other
GYS2	1.84E-02	−3.065	glycogen synthase 2 (liver)	Cytoplasm	enzyme
GZMH	3.19E-02	−2.741	granzyme H (cathepsin G-like 2, protein h-CCPX)	Cytoplasm	peptidase
H2AFJ	1.45E-02	1.677	H2A histone family, member J	Nucleus	other
H2AFY	2.50E-03	3.077	H2A histone family, member Y	Nucleus	other
HAL	2.12E-02	−2.903	histidine ammonia-lyase	Cytoplasm	enzyme
HAP1 (includes EG:15114)	7.85E-03	−1.706	huntingtin-associated protein 1	Cytoplasm	other
HAS1	7.24E-03	−2.265	hyaluronan synthase 1	Plasma Membrane	enzyme
HBG1	1.42E-02	1.784	hemoglobin, gamma A	Cytoplasm	other
HCAR3	7.43E-06	−3.738	hydroxycarboxylic acid receptor 3	Plasma Membrane	G-protein coupled receptor
HCFC1R1	1.10E-02	2.394	host cell factor C1 regulator 1 (XPO1 dependent)	Nucleus	other
HCG9	2.36E-03	2.222	HLA complex group 9 (non-protein coding)	unknown	other
HCST	1.40E-02	1.819	hematopoietic cell signal transducer	Plasma Membrane	other
HDAC5	1.92E-02	−1.747	histone deacetylase 5	Nucleus	transcription regulator
HDAC9	2.39E-03	−1.723	histone deacetylase 9	Nucleus	transcription regulator
HEATR1	4.26E-02	−2.965	HEAT repeat containing 1	Nucleus	other
HELQ	2.17E-02	−2.043	helicase, POLQ-like	Nucleus	enzyme
HEMGN	8.18E-03	4.249	hemogen	Nucleus	other
HEPH	4.32E-03	2.512	hephaestin	Plasma Membrane	transporter
HES2	2.86E-06	−2	hairy and enhancer of split 2 (Drosophila)	Nucleus	other
HES6	3.95E-02	1.623	hairy and enhancer of split 6 (Drosophila)	Nucleus	transcription regulator
HES1 (includes EG:15205)	1.28E-04	2.162	hairy and enhancer of split 1, (Drosophila)	Nucleus	transcription regulator
HHEX	4.06E-03	1.522	hematopoietically expressed homeobox	Nucleus	transcription regulator
HHLA1	5.11E-06	−1.702	HERV-H LTR-associating 1	unknown	other
HINT1	1.97E-03	−1.521	histidine triad nucleotide binding protein 1	Nucleus	enzyme
HIRA	5.71E-03	2.722	HIR histone cell cycle regulation defective homolog A (S. cerevisiae)	Nucleus	transcription regulator
HLA-DQB1	3.06E-02	3.362	major histocompatibility complex,	Plasma	other

			class II, DQ beta 1	Membrane	
HMGN2P46	1.20E-02	−1.761	high mobility group nucleosomal binding domain 2 pseudogene 46	unknown	other
HNF4A	2.02E-02	1.573	hepatocyte nuclear factor 4, alpha	Nucleus	transcription regulator
HOXA1	2.34E-05	1.774	homeobox A1	Nucleus	transcription regulator
HOXA10	1.25E-05	1.541	homeobox A10	Nucleus	transcription regulator
HOXB3	3.25E-02	2.416	homeobox B3	Nucleus	transcription regulator
HOXB7	4.85E-02	3.201	homeobox B7	Nucleus	transcription regulator
HOXB8	3.93E-04	2.235	homeobox B8	Nucleus	transcription regulator
HPCA	1.60E-02	1.7	hippocalcin	Cytoplasm	other
HR	2.65E-04	−1.619	hairless homolog (mouse)	Nucleus	transcription regulator
HRK	5.13E-03	2.233	harakiri, BCL2 interacting protein (contains only BH3 domain)	Cytoplasm	other
HRSP12	7.85E-03	3.868	heat-responsive protein 12	Cytoplasm	other
HS3ST1	5.28E-04	−2.78	heparan sulfate (glucosamine) 3-O-sulfotransferase 1	Cytoplasm	enzyme
HSPB8	7.36E-04	3.077	heat shock 22kDa protein 8	Cytoplasm	kinase
HSPH1	1.33E-06	1.594	heat shock 105kDa/110kDa protein 1	Cytoplasm	other
HTT	2.17E-02	2.641	huntingtin	Cytoplasm	transcription regulator
HYAL4	2.13E-02	−2.41	hyaluronoglucosaminidase 4	unknown	enzyme
IBTK	2.81E-02	4.329	inhibitor of Bruton agammaglobulinemia tyrosine kinase	Cytoplasm	other
ICOSLG	3.29E-02	−1.55	inducible T-cell co-stimulator ligand	Plasma Membrane	other
ID1	3.16E-04	−1.853	inhibitor of DNA binding 1, dominant negative helix-loop-helix protein	Nucleus	transcription regulator
ID2	4.00E-04	3.114	inhibitor of DNA binding 2, dominant negative helix-loop-helix protein	Nucleus	transcription regulator
ID3 (includes EG:15903)	3.10E-04	2.141	inhibitor of DNA binding 3, dominant negative helix-loop-helix protein	Nucleus	transcription regulator
IDUA	1.26E-02	−1.502	iduronidase, alpha-L-	Cytoplasm	enzyme
IFIH1	5.65E-03	−1.69	interferon induced with helicase C domain 1	Nucleus	enzyme

IFIT5	1.39E-02	2.099	interferon-induced protein with tetratricopeptide repeats 5	unknown	other
IFNA17	4.56E-02	−1.827	interferon, alpha 17	Extracellular Space	cytokine
IGFBP3	2.43E-05	−2.928	insulin-like growth factor binding protein 3	Extracellular Space	other
IGSF21	4.69E-03	−2.682	immunoglobin superfamily, member 21	unknown	other
IL20	8.18E-06	1.943	interleukin 20	Extracellular Space	cytokine
IL25	3.17E-02	2.023	interleukin 25	Extracellular Space	cytokine
IL17RC	4.30E-02	−1.526	interleukin 17 receptor C	Plasma Membrane	other
IL18BP	4.11E-04	−1.762	interleukin 18 binding protein	Extracellular Space	other
IL18R1	1.40E-02	1.575	interleukin 18 receptor 1	Plasma Membrane	transmembrane receptor
IL1RN	2.08E-06	−2.24	interleukin 1 receptor antagonist	Extracellular Space	cytokine
IL28RA	1.72E-05	−2.912	interleukin 28 receptor, alpha (interferon, lambda receptor)	Plasma Membrane	transmembrane receptor
IL36G	1.36E-07	−3.108	interleukin 36, gamma	Extracellular Space	cytokine
IMMP2L	6.75E-03	3.555	IMP2 inner mitochondrial membrane peptidase-like (S. cerevisiae)	Cytoplasm	peptidase
ING2	4.86E-02	2.393	inhibitor of growth family, member 2	Nucleus	transcription regulator
IQCD	6.56E-03	1.563	IQ motif containing D	unknown	other
IQCF4	2.84E-02	−1.518	IQ motif containing F5 pseudogene	unknown	other
IQGAP3	7.22E-04	−2.165	IQ motif containing GTPase activating protein 3	Plasma Membrane	other
IQUB	1.26E-02	−1.886	IQ motif and ubiquitin domain containing	Cytoplasm	other
IRAK1BP1	1.18E-04	1.771	interleukin-1 receptor-associated kinase 1 binding protein 1	unknown	other
IRF2	3.45E-05	−1.931	interferon regulatory factor 2	Nucleus	transcription regulator
ISG20	5.62E-04	−4.098	interferon stimulated exonuclease gene 20kDa	Nucleus	enzyme
ISG20L2	8.06E-03	4.099	interferon stimulated exonuclease gene 20kDa-like 2	Nucleus	enzyme
ISL1	7.66E-05	1.869	ISL LIM homeobox 1	Nucleus	transcription regulator
ITGAL	3.21E-02	1.504	integrin, alpha L (antigen CD11A	Plasma	other

			(p180), lymphocyte function-associated antigen 1; alpha polypeptide)	Membrane	
ITGB2	4.46E-02	−2.114	integrin, beta 2 (complement component 3 receptor 3 and 4 subunit)	Plasma Membrane	other
ITGBL1	3.36E-02	−2.286	integrin, beta-like 1 (with EGF-like repeat domains)	unknown	other
ITIH6	4.57E-02	−1.846	inter-alpha-trypsin inhibitor heavy chain family, member 6	unknown	other
ITPKC	3.06E-05	−1.798	inositol-trisphosphate 3-kinase C	Cytoplasm	kinase
ITPR1	6.25E-03	−1.549	inositol 1,4,5-trisphosphate receptor, type 1	Cytoplasm	ion channel
JAK3	3.85E-02	3.33	Janus kinase 3	Cytoplasm	kinase
JDP2	1.47E-04	1.752	Jun dimerization protein 2	Nucleus	transcription regulator
JHDM1D	3.56E-03	−1.627	jumonji C domain containing histone demethylase 1 homolog D (S. cerevisiae)	Nucleus	enzyme
JUN	1.76E-06	2.262	jun proto-oncogene	Nucleus	transcription regulator
KAL1	2.19E-02	−3.106	Kallmann syndrome 1 sequence	Extracellular Space	other
KBTBD7	1.51E-05	1.595	kelch repeat and BTB (POZ) domain containing 7	unknown	other
KCNA10	4.46E-03	−1.917	potassium voltage-gated channel, shaker-related subfamily, member 10	Plasma Membrane	ion channel
KCNH4	1.46E-03	1.553	potassium voltage-gated channel, subfamily H (eag-related), member 4	Plasma Membrane	ion channel
KCNH7	7.53E-03	2	potassium voltage-gated channel, subfamily H (eag-related), member 7	Plasma Membrane	ion channel
KCNJ1	1.07E-02	−2.081	potassium inwardly-rectifying channel, subfamily J, member 1	Plasma Membrane	ion channel
KCNJ2	6.88E-05	4.008	potassium inwardly-rectifying channel, subfamily J, member 2	Plasma Membrane	ion channel
KCNMA1	1.93E-02	1.54	potassium large conductance calcium-activated channel, subfamily M, alpha member 1	Plasma Membrane	ion channel
KCNN3	4.27E-02	3.285	potassium intermediate/small conductance calcium-activated channel, subfamily N, member 3	Plasma Membrane	ion channel
KCNS2	3.69E-02	−1.752	potassium voltage-gated channel, delayed-rectifier, subfamily S, member 2	Plasma Membrane	ion channel

KCTD7	1.59E-02	3.205	potassium channel tetramerisation domain containing 7	unknown	ion channel
KDM6B	4.04E-02	−2.738	lysine (K)-specific demethylase 6B	unknown	other
KIAA0146	2.78E-04	−1.608	KIAA0146	unknown	other
KIAA1377	2.55E-04	3.815	KIAA1377	unknown	other
KIAA1644	4.24E-04	−1.758	KIAA1644	unknown	other
KIF1C	4.06E-02	−1.888	kinesin family member 1C	Cytoplasm	other
KIF26A	2.82E-02	−2.789	kinesin family member 26A	Cytoplasm	other
KIRREL	2.86E-02	2.343	kin of IRRE like (Drosophila)	Plasma Membrane	other
KLC4	3.08E-02	−1.561	kinesin light chain 4	unknown	other
KLF5	2.40E-03	−1.51	Kruppel-like factor 5 (intestinal)	Nucleus	transcription regulator
KLHDC7B	9.63E-06	−2.754	kelch domain containing 7B	unknown	other
KLHL1	9.81E-03	−2.982	kelch-like 1 (Drosophila)	Cytoplasm	other
KLHL8	2.39E-03	2.627	kelch-like 8 (Drosophila)	unknown	other
KLHL21	1.36E-05	−1.656	kelch-like 21 (Drosophila)	Cytoplasm	enzyme
KLHL24	1.27E-02	−1.649	kelch-like 24 (Drosophila)	unknown	other
KLK10	4.04E-04	−2.02	kallikrein-related peptidase 10	Extracellular Space	peptidase
KLK15	2.39E-02	1.983	kallikrein-related peptidase 15	Extracellular Space	peptidase
KRT76	3.23E-03	−2.043	keratin 76	Cytoplasm	other
KSR1	2.49E-02	−1.876	kinase suppressor of ras 1	Cytoplasm	kinase
L3MBTL4	4.79E-02	2.105	l(3)mbt-like 4 (Drosophila)	unknown	other
LARP6	2.11E-04	1.656	La ribonucleoprotein domain family, member 6	unknown	other
LCA5	1.18E-03	3.063	Leber congenital amaurosis 5	unknown	other
LFNG	1.26E-03	1.679	LFNG O-fucosylpeptide 3-beta-N-acetylglucosaminyltransferase	Cytoplasm	enzyme
LHFPL2	5.41E-06	−2.202	lipoma HMGIC fusion partner-like 2	unknown	enzyme
LHX9	7.81E-03	2.74	LIM homeobox 9	Nucleus	transcription regulator
LINC00260	9.41E-04	−1.693	long intergenic non-protein coding RNA 260	unknown	other
LINC00339	6.92E-03	2.133	long intergenic non-protein coding RNA 339	unknown	other
LINC00487	2.34E-02	−3.113	long intergenic non-protein coding RNA 487	unknown	other
LINS	9.75E-03	−1.681	lines homolog (Drosophila)	unknown	other
LMO4	7.04E-04	2.519	LIM domain only 4	Nucleus	transcription regulator
LMO7	2.17E-02	−2.638	LIM domain 7	Cytoplasm	enzyme
LOC158376	4.37E-02	3.292	uncharacterized LOC158376	unknown	other
LOC253573	1.54E-02	3.011	uncharacterized LOC253573	unknown	other

LOC257396	2.50E-02	3.033	uncharacterized LOC257396	unknown	other
LOC283485	4.69E-05	3.146	uncharacterized LOC283485	unknown	other
LOC283486	3.86E-02	2.936	uncharacterized LOC283486	unknown	other
LOC283887	2.50E-02	1.977	uncharacterized LOC283887	unknown	other
LOC284454	3.78E-03	−1.758	uncharacterized LOC284454	unknown	other
LOC286370	3.29E-03	2.773	uncharacterized LOC286370	unknown	other
LOC339666	2.87E-02	−1.838	uncharacterized LOC339666	unknown	other
LOC339803	1.59E-05	2.479	uncharacterized LOC339803	unknown	other
LOC339822	7.26E-03	2.424	uncharacterized LOC339822	unknown	other
LOC389634	2.11E-02	−1.969	uncharacterized LOC389634	unknown	other
LOC440894	1.40E-02	−3.849	uncharacterized LOC440894	unknown	other
LOC644450	4.51E-02	3.483	uncharacterized LOC644450	unknown	other
LOC645638	4.05E-05	−2.41	WDNM1-like pseudogene	unknown	other
LOC646329	3.45E-02	1.534	uncharacterized LOC646329	unknown	other
LOC727787	3.14E-02	2.04	killer cell immunoglobulin-like receptor, three domains, long cytoplasmic tail, 2-like	unknown	other
LOC100128288	4.08E-02	3.28	uncharacterized LOC100128288	unknown	other
LOC100128398	3.70E-04	−3.875	uncharacterized LOC100128398	unknown	other
LOC100129961	1.45E-03	−3.41	uncharacterized LOC100129961	unknown	other
LOC100130078	1.25E-02	2.225	uncharacterized LOC100130078	unknown	other
LOC100130097	5.94E-03	1.746	kinesin-like protein family member 6-like	unknown	other
LOC100130557	2.28E-03	2.709	uncharacterized LOC100130557	unknown	other
LOC100131096	1.76E-02	−1.796	uncharacterized LOC100131096	unknown	other
LOC100131541	3.41E-02	−1.592	uncharacterized LOC100131541	unknown	other
LOC100132891	2.24E-02	1.8	uncharacterized LOC100132891	unknown	other
LOC100287387	2.17E-03	2.92	uncharacterized LOC100287387	unknown	other
LOC100288152	5.67E-04	−1.745	uncharacterized LOC100288152	unknown	other
LOC100288162	4.17E-02	1.509	uncharacterized LOC100288162	unknown	other
LOC100289045	6.29E-04	−2.789	uncharacterized LOC100289045	unknown	other
LOC100499489	2.52E-02	2.31	uncharacterized LOC100499489	unknown	other
LOC100505687	4.09E-02	3.033	uncharacterized LOC100505687	unknown	other
LOC100505854	1.57E-04	1.576	uncharacterized LOC100505854	unknown	other
LOC100506025	2.71E-02	3.433	uncharacterized LOC100506025	unknown	other
LOC100506033	6.34E-04	2.295	uncharacterized LOC100506033	unknown	other
LOC100506258	1.34E-02	3.725	uncharacterized LOC100506258	unknown	other
LOC100506325	1.99E-02	1.798	uncharacterized LOC100506325	unknown	other
LOC100507064	5.08E-03	1.947	uncharacterized LOC100507064	unknown	other
LOC100507203	9.07E-03	1.729	uncharacterized LOC100507203	unknown	other
LOC100507435	6.48E-03	−1.92	uncharacterized LOC100507435	unknown	other
LOC100507584	5.32E-03	4.333	uncharacterized LOC100507584	unknown	other
LOC100652730	2.97E-02	−2.269	uncharacterized LOC100652730	unknown	other
LOX	3.23E-02	−3.888	lysyl oxidase	Extracellular Space	enzyme
LPCAT2	5.16E-03	3.375	lysophosphatidylcholine	Cytoplasm	enzyme

			acyltransferase 2		
LPCAT4	6.97E-03	−2.904	lysophosphatidylcholine acyltransferase 4	unknown	other
LPIN3	4.20E-03	2.06	lipin 3	Nucleus	phosphatase
LRRC4C	1.36E-02	2.034	leucine rich repeat containing 4C	Plasma Membrane	other
LRRC8A	8.33E-04	−1.731	leucine rich repeat containing 8 family, member A	unknown	other
LRRC8E	4.93E-04	−1.66	leucine rich repeat containing 8 family, member E	unknown	other
LRRN1	4.92E-02	−2.945	leucine rich repeat neuronal 1	unknown	other
LY6D	1.88E-05	−1.555	lymphocyte antigen 6 complex, locus D	Plasma Membrane	other
LY86-AS1	1.18E-03	4.155	LY86 antisense RNA 1 (non-protein coding)	unknown	other
LYPD3	5.55E-05	−2.162	LY6/PLAUR domain containing 3	Plasma Membrane	other
LYPD6	2.85E-02	2.054	LY6/PLAUR domain containing 6	Extracellular Space	other
MAB21L3	8.40E-03	1.568	mab-21-like 3 (C. elegans)	unknown	other
MACF1	4.08E-02	1.962	microtubule-actin crosslinking factor 1	Cytoplasm	enzyme
MAFB	3.61E-04	1.532	v-maf musculoaponeurotic fibrosarcoma oncogene homolog B (avian)	Nucleus	other
MAFIP/TEKT4P1	2.83E-02	−1.913	MAFF interacting protein	unknown	other
MAFK	2.11E-06	−1.555	v-maf musculoaponeurotic fibrosarcoma oncogene homolog K (avian)	Nucleus	transcription regulator
MAP4	1.07E-02	−2.136	microtubule-associated protein 4	Cytoplasm	other
MAP9	5.19E-04	1.535	microtubule-associated protein 9	unknown	other
MAP3K8	7.36E-04	−1.961	mitogen-activated protein kinase kinase kinase 8	Cytoplasm	kinase
MAPK7	4.19E-02	−1.643	mitogen-activated protein kinase 7	Cytoplasm	kinase
1-Mar	3.77E-03	2.347	mitochondrial amidoxime reducing component 1	Cytoplasm	enzyme
MARCKSL1	1.01E-07	−3.155	MARCKS-like 1	Cytoplasm	other
MAST4	1.39E-06	−1.706	microtubule associated serine/threonine kinase family member 4	unknown	kinase
MAU2	5.81E-04	−2.871	MAU2 chromatid cohesion factor homolog (C. elegans)	Nucleus	other
MAZ	2.31E-02	1.741	MYC-associated zinc finger protein (purine-binding transcription factor)	Nucleus	transcription regulator

MBNL2	4.88E-04	2.834	muscleblind-like 2 (Drosophila)	unknown	other
MBP	3.85E-02	−2.895	myelin basic protein	Extracellular Space	other
MCM5	2.54E-03	1.619	minichromosome maintenance complex component 5	Nucleus	enzyme
MCOLN3	3.38E-03	1.546	mucolipin 3	Plasma Membrane	ion channel
MDM4	3.62E-03	2.578	Mdm4 p53 binding protein homolog (mouse)	Nucleus	other
MDS2	2.81E-02	1.916	myelodysplastic syndrome 2 translocation associated	unknown	other
MECP2	8.01E-03	2.768	methyl CpG binding protein 2 (Rett syndrome)	Nucleus	transcription regulator
MED20	1.96E-03	2.022	mediator complex subunit 20	Nucleus	transcription regulator
MED27	1.29E-03	2.715	mediator complex subunit 27	Nucleus	transcription regulator
MEOX1	4.94E-03	−1.7	mesenchyme homeobox 1	Nucleus	transcription regulator
MEOX2	4.56E-02	−2.372	mesenchyme homeobox 2	Nucleus	transcription regulator
METRNL	3.50E-05	−2.064	meteorin, glial cell differentiation regulator-like	unknown	other
METTL21C	7.49E-03	2.907	methyltransferase like 21C	unknown	other
MFHAS1	8.94E-05	−1.733	malignant fibrous histiocytoma amplified sequence 1	Cytoplasm	other
MIB1	4.42E-02	−3.162	mindbomb homolog 1 (Drosophila)	Cytoplasm	other
MIPOL1	2.31E-02	2.606	mirror-image polydactyly 1	unknown	other
MIR210HG	3.00E-02	−2.359	MIR210 host gene (non-protein coding)	unknown	other
MKL2	5.04E-04	2.968	MKL/myocardin-like 2	Nucleus	transcription regulator
MLL	3.79E-02	−2.459	myeloid/lymphoid or mixed-lineage leukemia (trithorax homolog, Drosophila)	Nucleus	transcription regulator
MLLT3	1.59E-04	2.883	myeloid/lymphoid or mixed-lineage leukemia (trithorax homolog, Drosophila); translocated to, 3	Nucleus	other
MLLT6	4.73E-04	3.832	myeloid/lymphoid or mixed-lineage leukemia (trithorax homolog, Drosophila); translocated to, 6	Nucleus	transcription regulator
MMP15	3.52E-03	−1.931	matrix metallopeptidase 15 (membrane-inserted)	Extracellular Space	peptidase
MMP16	4.92E-02	2.255	matrix metallopeptidase 16	Extracellular	peptidase

			(membrane-inserted)	Space	
MMP24	4.69E-02	1.897	matrix metallopeptidase 24 (membrane-inserted)	Extracellular Space	peptidase
MNT	4.84E-05	−2.486	MAX binding protein	Nucleus	transcription regulator
MORN4	1.63E-04	1.818	MORN repeat containing 4	unknown	other
MPP4	7.54E-03	3.346	membrane protein, palmitoylated 4 (MAGUK p55 subfamily member 4)	Cytoplasm	kinase
MS4A4A	8.93E-04	3.907	membrane-spanning 4-domains, subfamily A, member 4	unknown	other
MSRB2	1.39E-03	1.642	methionine sulfoxide reductase B2	Nucleus	transcription regulator
MTMR1	3.62E-02	2.844	myotubularin related protein 1	Cytoplasm	phosphatase
MTRF1	5.17E-03	1.855	mitochondrial translational release factor 1	Cytoplasm	translation regulator
MXD1	4.82E-06	−1.708	MAX dimerization protein 1	Nucleus	transcription regulator
MYBPC3	2.18E-02	−1.653	myosin binding protein C, cardiac	Cytoplasm	other
MYCL1	1.51E-02	−1.945	v-myc myelocytomatosis viral oncogene homolog 1, lung carcinoma derived (avian)	Nucleus	transcription regulator
MYLIP	3.47E-03	1.7	myosin regulatory light chain interacting protein	Cytoplasm	enzyme
MYPN	4.41E-02	2.926	myopalladin	Cytoplasm	other
MYT1L	1.78E-02	−2.838	myelin transcription factor 1-like	Nucleus	transcription regulator
N4BP1	3.66E-05	−1.795	NEDD4 binding protein 1	Cytoplasm	other
NACAD	3.41E-02	−1.784	NAC alpha domain containing	unknown	other
NAP1L3	4.57E-02	4.099	nucleosome assembly protein 1-like 3	Nucleus	other
NBPF5	9.61E-03	3.468	neuroblastoma breakpoint family, member 5	unknown	other
NCALD	4.32E-02	1.683	neurocalcin delta	Cytoplasm	other
NCAM2	2.28E-03	−3.125	neural cell adhesion molecule 2	Plasma Membrane	other
NDEL1	2.92E-02	−2.1	nudE nuclear distribution gene E homolog (A. nidulans)-like 1	Nucleus	other
NDUFA7	2.77E-02	−2.159	NADH dehydrogenase (ubiquinone) 1 alpha subcomplex, 7, 14.5kDa	Cytoplasm	enzyme
NECAB1	2.97E-02	3.051	N-terminal EF-hand calcium binding protein 1	Cytoplasm	other
NEDD9	2.57E-05	2.174	neural precursor cell expressed, developmentally down-regulated 9	Nucleus	other
NEU2	1.64E-02	−2.16	sialidase 2 (cytosolic sialidase)	Cytoplasm	enzyme

NEXN	1.76E-04	1.596	nexilin (F actin binding protein)	Plasma Membrane	other
NFIX	1.35E-05	−2.521	nuclear factor I/X (CCAAT-binding transcription factor)	Nucleus	transcription regulator
NFKBIA	2.31E-04	−1.572	nuclear factor of kappa light polypeptide gene enhancer in B-cells inhibitor, alpha	Cytoplasm	other
NFKBIZ	5.60E-07	−1.86	nuclear factor of kappa light polypeptide gene enhancer in B-cells inhibitor, zeta	Nucleus	transcription regulator
NGEF	1.62E-02	2.711	neuronal guanine nucleotide exchange factor	Cytoplasm	other
NGF	2.95E-03	3.18	nerve growth factor (beta polypeptide)	Extracellular Space	growth factor
NHLH1	1.97E-02	1.702	nescient helix loop helix 1	Nucleus	other
NID1	4.57E-02	2.03	nidogen 1	Extracellular Space	other
NKAP	2.33E-02	3.363	NFKB activating protein	Nucleus	transcription regulator
NKX6-2	2.63E-03	1.596	NK6 homeobox 2	Nucleus	transcription regulator
NLRP14	2.31E-02	−2.119	NLR family, pyrin domain containing 14	unknown	other
NOL3	9.26E-03	1.523	nucleolar protein 3 (apoptosis repressor with CARD domain)	Nucleus	other
NPAS3	1.09E-02	2.392	neuronal PAS domain protein 3	Nucleus	other
NPHP3	2.83E-02	2.071	nephronophthisis 3 (adolescent)	Extracellular Space	other
NPR2 (includes EG:116564)	4.45E-04	1.563	natriuretic peptide receptor B/guanylate cyclase B (atrionatriuretic peptide receptor B)	Plasma Membrane	G-protein coupled receptor
NPR3 (includes EG:18162)	5.04E-03	−3.043	natriuretic peptide receptor C/guanylate cyclase C (atrionatriuretic peptide receptor C)	Plasma Membrane	G-protein coupled receptor
NR3C1	4.40E-03	1.573	nuclear receptor subfamily 3, group C, member 1 (glucocorticoid receptor)	Nucleus	ligand-dependent nuclear receptor
NR6A1	7.56E-03	−2.17	nuclear receptor subfamily 6, group A, member 1	Nucleus	ligand-dependent nuclear receptor
NSAP11	3.97E-02	2.716	nervous system abundant protein 11	unknown	other
NSMCE4A	4.08E-02	2.074	non-SMC element 4 homolog A (S. cerevisiae)	Nucleus	other

NTHL1	5.35E-04	1.635	nth endonuclease III-like 1 (E. coli)	Nucleus	enzyme
NTN5	5.05E-03	2.895	netrin 5	unknown	other
NUAK1	2.69E-06	1.857	NUAK family, SNF1-like kinase, 1	unknown	kinase
NVL	2.48E-04	3.522	nuclear VCP-like	Nucleus	other
OAF	5.89E-03	−2.075	OAF homolog (Drosophila)	unknown	other
OBFC1	1.70E-02	1.661	oligonucleotide/oligosaccharide-binding fold containing 1	Nucleus	other
OBSL1	3.74E-04	3.777	obscurin-like 1	Plasma Membrane	other
OCLN	8.34E-05	−1.647	occludin	Plasma Membrane	other
OCM2	2.19E-02	2.137	oncomodulin 2	unknown	other
OLFM3	2.64E-02	−3.496	olfactomedin 3	Cytoplasm	other
OLIG2	4.30E-02	2.135	oligodendrocyte lineage transcription factor 2	Nucleus	transcription regulator
ORC1 (includes EG:18392)	4.67E-02	3.113	origin recognition complex, subunit 1	Nucleus	other
OTOA	1.79E-02	−3.468	otoancorin	Extracellular Space	other
OVOL1	2.59E-07	−2.495	ovo-like 1(Drosophila)	Nucleus	transcription regulator
P2RX1	3.99E-02	−2.376	purinergic receptor P2X, ligand-gated ion channel, 1	Plasma Membrane	ion channel
PACSIN1	4.81E-02	−1.724	protein kinase C and casein kinase substrate in neurons 1	Cytoplasm	kinase
PAK1IP1	1.97E-04	1.538	PAK1 interacting protein 1	Nucleus	other
PANX2	4.19E-02	−1.854	pannexin 2	Plasma Membrane	transporter
PARP8	4.27E-03	1.78	poly (ADP-ribose) polymerase family, member 8	unknown	other
PART1	4.75E-02	1.743	prostate androgen-regulated transcript 1 (non-protein coding)	unknown	other
PAX1	3.08E-02	2.038	paired box 1	Nucleus	transcription regulator
PCDH18	3.32E-03	1.542	protocadherin 18	Extracellular Space	other
PCNP	3.92E-02	2.071	PEST proteolytic signal containing nuclear protein	Nucleus	other
PDCD5	2.71E-03	−1.732	programmed cell death 5	Nucleus	other
PDCD1LG2 (includes EG:309304)	4.21E-02	2.075	programmed cell death 1 ligand 2	Plasma Membrane	enzyme
PDLIM3	8.95E-03	3.136	PDZ and LIM domain 3	Cytoplasm	other
PDS5B	7.10E-03	1.506	PDS5, regulator of cohesion maintenance, homolog B (S. cerevisiae)	Nucleus	other

PDXDC1	4.34E-02	−2.207	pyridoxal-dependent decarboxylase domain containing 1	unknown	other
PER3	2.33E-04	4.095	period homolog 3 (Drosophila)	Nucleus	other
PET117 (human)	4.15E-04	1.592	cytochrome c oxidase assembly factor-like	unknown	enzyme
PEX2 (includes EG:19302)	1.51E-02	2.485	peroxisomal biogenesis factor 2	Cytoplasm	other
PF4V1	1.57E-02	4.696	platelet factor 4 variant 1	Extracellular Space	cytokine
PGBD1	2.78E-03	1.872	piggyBac transposable element derived 1	unknown	enzyme
PHF11	7.23E-03	−1.836	PHD finger protein 11	unknown	other
PHF17	1.11E-04	1.719	PHD finger protein 17	Nucleus	other
PIGM	3.06E-02	1.507	phosphatidylinositol glycan anchor biosynthesis, class M	Cytoplasm	enzyme
PIK3C2A	5.04E-03	3.967	phosphoinositide-3-kinase, class 2, alpha polypeptide	Cytoplasm	kinase
PIK3IP1	1.06E-04	−1.601	phosphoinositide-3-kinase interacting protein 1	unknown	other
PIM1	8.23E-06	−2.061	pim-1 oncogene	Cytoplasm	kinase
PIM3	9.08E-04	−1.628	pim-3 oncogene	unknown	kinase
PKP2 (includes EG:287925)	6.74E-03	2.483	plakophilin 2	Plasma Membrane	other
PLAC2	1.18E-04	−1.686	placenta-specific 2 (non-protein coding)	unknown	other
PLAC4	3.35E-02	2.247	placenta-specific 4	unknown	other
PLAC9	4.11E-02	1.905	placenta-specific 9	unknown	other
PLAT	2.19E-04	−1.589	plasminogen activator, tissue	Extracellular Space	peptidase
PLAUR	5.78E-06	−2.138	plasminogen activator, urokinase receptor	Plasma Membrane	transmembrane receptor
PLCB1	3.77E-02	2.269	phospholipase C, beta 1 (phosphoinositide-specific)	Cytoplasm	enzyme
PLEKHA7	3.58E-03	−3.989	pleckstrin homology domain containing, family A member 7	Cytoplasm	other
PLEKHA8	9.22E-03	3.752	pleckstrin homology domain containing, family A (phosphoinositide binding specific) member 8	unknown	other
PLEKHM1	5.69E-04	−1.51	pleckstrin homology domain containing, family M (with RUN domain) member 1	Cytoplasm	other
PLG	1.17E-02	3.062	plasminogen	Extracellular Space	peptidase
PLGLB1/PLGLB2	4.25E-03	1.551	plasminogen-like B2	Extracellular Space	peptidase

PLK5	1.05E-02	2.093	polo-like kinase 5	Cytoplasm	other
PML	1.41E-04	−1.578	promyelocytic leukemia	Nucleus	transcription regulator
PNRC1	2.56E-03	−1.543	proline-rich nuclear receptor coactivator 1	Nucleus	other
POLR3B	5.44E-03	3.563	polymerase (RNA) III (DNA directed) polypeptide B	Nucleus	enzyme
PP2672	3.92E-03	1.695	uncharacterized LOC100130249	unknown	other
PPARD	6.59E-06	−1.721	peroxisome proliferator-activated receptor delta	Nucleus	ligand-dependent nuclear receptor
PPARGC1B	1.45E-03	−1.558	peroxisome proliferator-activated receptor gamma, coactivator 1 beta	Nucleus	transcription regulator
PPCDC	1.81E-03	2.073	phosphopantothenoylcysteine decarboxylase	Cytoplasm	enzyme
PPP1R36	4.61E-04	3.178	protein phosphatase 1, regulatory subunit 36	unknown	other
PPP2R3B	1.37E-02	2.008	protein phosphatase 2, regulatory subunit B", beta	Nucleus	phosphatase
PPP4R1L	3.15E-02	−2.357	protein phosphatase 4, regulatory subunit 1-like	unknown	other
PPP6R2	4.07E-02	2.587	protein phosphatase 6, regulatory subunit 2	Cytoplasm	other
PQLC3	4.61E-02	−2.09	PQ loop repeat containing 3	unknown	other
PRAC	5.71E-03	2.568	prostate cancer susceptibility candidate	unknown	other
PRDM1	3.92E-07	−2.331	PR domain containing 1, with ZNF domain	Nucleus	transcription regulator
PRIC285	1.41E-02	−1.925	peroxisomal proliferator-activated receptor A interacting complex 285	Nucleus	transcription regulator
PRIM1	2.21E-03	1.633	primase, DNA, polypeptide 1 (49kDa)	Nucleus	enzyme
PRKCE	1.13E-02	2.847	protein kinase C, epsilon	Cytoplasm	kinase
PRKRA	8.77E-03	1.921	protein kinase, interferon-inducible double stranded RNA dependent activator	Cytoplasm	other
PROC	3.03E-02	1.675	protein C (inactivator of coagulation factors Va and VIIIa)	Extracellular Space	peptidase
PRPF40B	1.33E-04	1.538	PRP40 pre-mRNA processing factor 40 homolog B (S. cerevisiae)	Nucleus	other
PSAPL1	4.83E-03	1.933	prosaposin-like 1 (gene/pseudogene)	unknown	other
PSG7	4.00E-02	2.977	pregnancy specific beta-1-glycoprotein 7 (gene/pseudogene)	Extracellular Space	other
PSMG1	8.93E-04	−1.684	proteasome (prosome, macropain)	Plasma	other

			assembly chaperone 1	Membrane	
PTBP1	1.72E-02	−1.812	polypyrimidine tract binding protein 1	Nucleus	enzyme
PTGER3	6.46E-03	1.928	prostaglandin E receptor 3 (subtype EP3)	Plasma Membrane	G-protein coupled receptor
PTGER4	1.47E-06	3.238	prostaglandin E receptor 4 (subtype EP4)	Plasma Membrane	G-protein coupled receptor
PTPDC1	7.13E-04	1.705	protein tyrosine phosphatase domain containing 1	unknown	phosphatase
PTPN1	3.07E-02	2.024	protein tyrosine phosphatase, non-receptor type 1	Cytoplasm	phosphatase
PTPRA	4.95E-02	2.206	protein tyrosine phosphatase, receptor type, A	Plasma Membrane	phosphatase
PUM2	2.66E-02	−1.923	pumilio homolog 2 (Drosophila)	Cytoplasm	other
PVRL1	8.91E-04	−1.674	poliovirus receptor-related 1 (herpesvirus entry mediator C)	Plasma Membrane	other
PVRL4	9.17E-05	−1.798	poliovirus receptor-related 4	Plasma Membrane	other
PXMP4	1.05E-02	2.384	peroxisomal membrane protein 4, 24kDa	Cytoplasm	other
RAB35	3.35E-03	−2.046	RAB35, member RAS oncogene family	Cytoplasm	enzyme
RAD9B	4.08E-03	3.188	RAD9 homolog B (S. pombe)	Nucleus	other
RALGDS	5.09E-04	−3.016	ral guanine nucleotide dissociation stimulator	Cytoplasm	other
RALGPS2	6.49E-03	−1.57	Ral GEF with PH domain and SH3 binding motif 2	unknown	other
RAPGEF2	6.96E-03	−2.53	Rap guanine nucleotide exchange factor (GEF) 2	Cytoplasm	other
RARB	1.56E-02	1.731	retinoic acid receptor, beta	Nucleus	ligand-dependent nuclear receptor
RASAL2	2.79E-03	2.895	RAS protein activator like 2	unknown	other
RASD1	8.86E-07	3.3	RAS, dexamethasone-induced 1	Cytoplasm	enzyme
RASGRF2	4.92E-02	2.635	Ras protein-specific guanine nucleotide-releasing factor 2	Cytoplasm	other
RASSF10	1.76E-03	−1.743	Ras association (RalGDS/AF-6) domain family (N-terminal) member 10	unknown	other
RBBP8	1.66E-06	1.505	retinoblastoma binding protein 8	Nucleus	enzyme
RBM25	3.39E-03	2.933	RNA binding motif protein 25	Nucleus	other
RBM33	2.56E-03	2.064	RNA binding motif protein 33	unknown	other
RC3H2	1.05E-03	1.626	ring finger and CCCH-type	Plasma	other

			domains 2	Membrane	
RCN3	5.64E-03	2.65	reticulocalbin 3, EF-hand calcium binding domain	Cytoplasm	other
RDH13	3.38E-08	−1.972	retinol dehydrogenase 13 (all-trans/9-cis)	Cytoplasm	enzyme
RECQL4	4.46E-02	2.071	RecQ protein-like 4	Nucleus	enzyme
REV3L	5.11E-04	1.53	REV3-like, catalytic subunit of DNA polymerase zeta (yeast)	Nucleus	enzyme
RFXAP	4.29E-06	3.392	regulatory factor X-associated protein	Nucleus	transcription regulator
RG9MTD3	6.00E-05	2.743	RNA (guanine-9-) methyltransferase domain containing 3	unknown	other
RGMA	3.18E-03	−2.64	RGM domain family, member A	Plasma Membrane	other
RGS5	4.11E-04	2.338	regulator of G-protein signaling 5	Plasma Membrane	other
RGS8	4.80E-04	2.742	regulator of G-protein signaling 8	Cytoplasm	other
RGS9	3.69E-02	2.151	regulator of G-protein signaling 9	Cytoplasm	enzyme
RGS12	1.83E-02	−2.32	regulator of G-protein signaling 12	Nucleus	other
RHBDL3	4.12E-02	−1.575	rhomboid, veinlet-like 3 (Drosophila)	Plasma Membrane	peptidase
RHOH	4.65E-03	−2.451	ras homolog gene family, member H	Plasma Membrane	enzyme
RHOV	6.95E-05	−2.466	ras homolog gene family, member V	Plasma Membrane	enzyme
RIPPLY1	1.95E-02	2.369	ripply1 homolog (zebrafish)	Nucleus	other
RNASE2	2.63E-02	2.203	ribonuclease, RNase A family, 2 (liver, eosinophil-derived neurotoxin)	Cytoplasm	enzyme
RNF152	3.80E-02	−1.85	ring finger protein 152	Cytoplasm	enzyme
RNF165	3.96E-02	2.561	ring finger protein 165	unknown	other
RNF19B	6.16E-05	−1.791	ring finger protein 19B	unknown	other
RNPC3	4.02E-02	−2.051	RNA-binding region (RNP1, RRM) containing 3	Nucleus	other
RORA	3.60E-02	−2.833	RAR-related orphan receptor A	Nucleus	ligand-dependent nuclear receptor
RPH3AL	9.16E-04	1.759	rabphilin 3A-like (without C2 domains)	Plasma Membrane	other
RPL35A	2.03E-02	1.652	ribosomal protein L35a	Cytoplasm	other
RRP1B	6.82E-03	2.543	ribosomal RNA processing 1 homolog B (S. cerevisiae)	Nucleus	other
RSAD2	3.20E-02	−2.072	radical S-adenosyl methionine domain containing 2	Cytoplasm	enzyme

RTKN2	1.13E-03	−2.896	rhotekin 2	Plasma Membrane	other
RTN4RL2	4.13E-02	−2.645	reticulon 4 receptor-like 2	Plasma Membrane	other
RUFY1	8.60E-03	−2.069	RUN and FYVE domain containing 1	Cytoplasm	transporter
RUNDC1	3.73E-02	−3.041	RUN domain containing 1	unknown	other
RUNDC3B	2.89E-03	2.069	RUN domain containing 3B	unknown	other
RUSC2	2.66E-03	2.331	RUN and SH3 domain containing 2	unknown	other
S100A7A	2.38E-02	1.572	S100 calcium binding protein A7A	Cytoplasm	other
S1PR1	4.30E-02	−2.819	sphingosine-1-phosphate receptor 1	Plasma Membrane	G-protein coupled receptor
SARS	2.58E-02	3.024	seryl-tRNA synthetase	Cytoplasm	enzyme
SAT1	3.06E-02	2.15	spermidine/spermine N1-acetyltransferase 1	Cytoplasm	enzyme
SCGB2A2	5.18E-05	3.54	secretoglobin, family 2A, member 2	Extracellular Space	other
SCGB3A1	6.55E-03	−2.541	secretoglobin, family 3A, member 1	Extracellular Space	cytokine
SCN3B	1.34E-02	−2.738	sodium channel, voltage-gated, type III, beta	Plasma Membrane	ion channel
SCUBE2	3.07E-02	1.61	signal peptide, CUB domain, EGF-like 2	unknown	other
SDCBP2	4.25E-04	−1.883	syndecan binding protein (syntenin) 2	Cytoplasm	other
SDHAP1	2.83E-05	−1.519	succinate dehydrogenase complex, subunit A, flavoprotein pseudogene 1	unknown	other
SDHB	1.73E-02	−3.239	succinate dehydrogenase complex, subunit B, iron sulfur (Ip)	Cytoplasm	enzyme
SDPR	6.52E-03	1.787	serum deprivation response	Plasma Membrane	other
SEC63 (includes EG:11231)	2.39E-02	2.207	SEC63 homolog (S. cerevisiae)	Cytoplasm	transporter
SEMA3A	2.71E-03	−2.374	sema domain, immunoglobulin domain (Ig), short basic domain, secreted, (semaphorin) 3A	Extracellular Space	other
SEMA4C	1.72E-02	1.977	sema domain, immunoglobulin domain (Ig), transmembrane domain (TM) and short cytoplasmic domain, (semaphorin) 4C	Plasma Membrane	other
SERPINB1	5.31E-05	−1.764	serpin peptidase inhibitor, clade B (ovalbumin), member 1	Cytoplasm	other

SERPINB9	2.65E-05	−2.97	serpin peptidase inhibitor, clade B (ovalbumin), member 9	Cytoplasm	other
SERPINE1	3.62E-03	2.164	serpin peptidase inhibitor, clade E (nexin, plasminogen activator inhibitor type 1), member 1	Extracellular Space	other
SETDB2	3.26E-02	1.516	SET domain, bifurcated 2	Nucleus	enzyme
SFTPC	3.24E-02	−1.553	surfactant protein C	Extracellular Space	other
SH2D1A	1.20E-02	−2.162	SH2 domain containing 1A	Cytoplasm	other
SH2D3C	6.36E-03	2.1	SH2 domain containing 3C	Cytoplasm	other
SH3BP5L	1.46E-02	−1.66	SH3-binding domain protein 5-like	unknown	other
SHISA2	2.04E-05	−2.582	shisa homolog 2 (Xenopus laevis)	unknown	other
SHOX2	3.17E-02	3.364	short stature homeobox 2	Nucleus	transcription regulator
SHQ1	2.61E-03	1.672	SHQ1 homolog (S. cerevisiae)	Nucleus	other
SIAE	3.20E-02	−4.354	sialic acid acetylesterase	Cytoplasm	enzyme
SIKE1	1.76E-02	1.795	suppressor of IKBKE 1	Cytoplasm	other
SIN3A	3.36E-02	−2.618	SIN3 transcription regulator homolog A (yeast)	Nucleus	transcription regulator
SIRPB1	4.89E-04	4.533	signal-regulatory protein beta 1	Plasma Membrane	other
SKP2 (includes EG:27401)	5.13E-05	2.533	S-phase kinase-associated protein 2 (p45)	Nucleus	other
SLC12A4	1.14E-02	2.514	solute carrier family 12 (potassium/chloride transporters), member 4	Plasma Membrane	transporter
SLC12A6	1.89E-04	−1.765	solute carrier family 12 (potassium/chloride transporters), member 6	Plasma Membrane	transporter
SLC15A1	8.41E-05	3.494	solute carrier family 15 (oligopeptide transporter), member 1	Plasma Membrane	transporter
SLC15A3	2.19E-02	−2.357	solute carrier family 15, member 3	Cytoplasm	transporter
SLC1A4	2.84E-04	1.503	solute carrier family 1 (glutamate/neutral amino acid transporter), member 4	Plasma Membrane	transporter
SLC20A1	3.49E-04	1.979	solute carrier family 20 (phosphate transporter), member 1	Plasma Membrane	transporter
SLC22A17	2.33E-02	3.548	solute carrier family 22, member 17	Plasma Membrane	transporter
SLC24A3	2.70E-02	−2.217	solute carrier family 24 (sodium/potassium/calcium exchanger), member 3	Plasma Membrane	transporter
SLC25A30	2.59E-02	1.899	solute carrier family 25, member 30	Cytoplasm	other
SLC25A45	1.12E-03	−3.263	solute carrier family 25, member	Cytoplasm	transporter

			45		
SLC2A3	7.94E-06	−1.893	solute carrier family 2 (facilitated glucose transporter), member 3	Plasma Membrane	transporter
SLC38A10	4.97E-02	3.088	solute carrier family 38, member 10	unknown	other
SLC3A1	1.30E-02	2.34	solute carrier family 3 (cystine, dibasic and neutral amino acid transporters, activator of cystine, dibasic and neutral amino acid transport), member 1	Plasma Membrane	transporter
SLC44A4	5.25E-03	−2.853	solute carrier family 44, member 4	Plasma Membrane	transporter
SLC4A9	3.76E-02	−2.54	solute carrier family 4, sodium bicarbonate cotransporter, member 9	Plasma Membrane	transporter
SLC5A9	1.07E-02	−1.754	solute carrier family 5 (sodium/glucose cotransporter), member 9	Plasma Membrane	transporter
SLC5A10	3.13E-02	−1.716	solute carrier family 5 (sodium/glucose cotransporter), member 10	unknown	transporter
SLC5A12	1.61E-02	3.13	solute carrier family 5 (sodium/glucose cotransporter), member 12	unknown	transporter
SLC6A20	1.47E-03	2.279	solute carrier family 6 (proline IMINO transporter), member 20	Plasma Membrane	transporter
SLC7A7	2.85E-02	1.537	solute carrier family 7 (amino acid transporter light chain, y+L system), member 7	Plasma Membrane	transporter
SLC7A8	1.28E-02	−3.355	solute carrier family 7 (amino acid transporter light chain, L system), member 8	Plasma Membrane	transporter
SLC9A2	4.74E-02	−2.328	solute carrier family 9 (sodium/hydrogen exchanger), member 2	Plasma Membrane	transporter
SLCO3A1	1.16E-04	−1.582	solute carrier organic anion transporter family, member 3A1	Plasma Membrane	transporter
SLFN13	4.35E-02	3.487	schlafen family member 13	Nucleus	enzyme
SMARCAL1	1.33E-06	1.511	SWI/SNF related, matrix associated, actin dependent regulator of chromatin, subfamily a-like 1	Nucleus	enzyme
SMCR8	2.78E-02	2.153	Smith-Magenis syndrome chromosome region, candidate 8	unknown	other
SMTNL1	4.19E-02	2.281	smoothelin-like 1	Cytoplasm	other
SNAI1	1.17E-05	3.796	snail homolog 1 (Drosophila)	Nucleus	transcription regulator

SNRPB2	4.66E-02	−2.426	small nuclear ribonucleoprotein polypeptide B	Nucleus	other
SNRPN	3.37E-04	2.611	small nuclear ribonucleoprotein polypeptide N	Nucleus	other
SNTB1	1.87E-02	1.535	syntrophin, beta 1 (dystrophin-associated protein A1, 59kDa, basic component 1)	Plasma Membrane	other
SNX18	5.32E-03	1.874	sorting nexin 18	Cytoplasm	transporter
SNX21	1.31E-02	1.877	sorting nexin family member 21	unknown	transporter
SNX25	1.30E-02	2.538	sorting nexin 25	unknown	other
SNX29	1.77E-02	1.733	sorting nexin 29	unknown	other
SOD2	4.12E-05	−1.598	superoxide dismutase 2, mitochondrial	Cytoplasm	enzyme
SORBS1	3.93E-03	3.623	sorbin and SH3 domain containing 1	Plasma Membrane	other
SOX2	2.50E-03	2.976	SRY (sex determining region Y)-box 2	Nucleus	transcription regulator
SOX9	2.16E-04	−1.628	SRY (sex determining region Y)-box 9	Nucleus	transcription regulator
SOX13	4.66E-03	−1.704	SRY (sex determining region Y)-box 13	Nucleus	transcription regulator
SPAG11A	1.40E-02	2.957	sperm associated antigen 11A	unknown	other
SPANXA2-OT1	3.02E-02	1.883	SPANXA2 overlapping transcript 1 (non-protein coding)	unknown	other
SPATA22	4.28E-02	2.869	spermatogenesis associated 22	unknown	other
SPG20OS	3.65E-02	−2.94	SPG20 opposite strand	unknown	other
SPPL3	6.35E-05	−1.578	signal peptide peptidase-like 3	Plasma Membrane	peptidase
SPRED1	1.85E-02	3.78	sprouty-related, EVH1 domain containing 1	Plasma Membrane	other
SRD5A2	2.46E-02	2.14	steroid-5-alpha-reductase, alpha polypeptide 2 (3-oxo-5 alpha-steroid delta 4-dehydrogenase alpha 2)	Cytoplasm	enzyme
SREK1	4.72E-02	−1.798	splicing regulatory glutamine/lysine-rich protein 1	Nucleus	other
SRG7	5.82E-03	1.745	spermatogenesis-related protein 7	unknown	other
SRGAP1	2.42E-05	4.501	SLIT-ROBO Rho GTPase activating protein 1	Cytoplasm	other
SRSF11	4.56E-02	2.946	serine/arginine-rich splicing factor 11	Nucleus	other
SRSF12	1.90E-02	−1.749	serine/arginine-rich splicing factor 12	Nucleus	other
SSX2IP	2.38E-03	2.599	synovial sarcoma, X breakpoint 2 interacting protein	Plasma Membrane	other
ST14	2.85E-05	−1.87	suppression of tumorigenicity 14	Plasma	peptidase

			(colon carcinoma)	Membrane	
ST3GAL3	1.05E-03	−2.098	ST3 beta-galactoside alpha-2,3-sialyltransferase 3	unknown	enzyme
ST6GAL2	3.21E-02	−2.157	ST6 beta-galactosamide alpha-2,6-sialyltranferase 2	Cytoplasm	enzyme
ST6GALNAC1	3.36E-02	2.396	ST6 (alpha-N-acetyl-neuraminyl-2,3-beta-galactosyl-1,3)-N-acetylgalactosaminide alpha-2,6-sialyltransferase 1	Cytoplasm	enzyme
ST8SIA5	8.15E-03	−2.682	ST8 alpha-N-acetyl-neuraminide alpha-2,8-sialyltransferase 5	Cytoplasm	enzyme
STAM2	5.38E-06	2.864	signal transducing adaptor molecule (SH3 domain and ITAM motif) 2	Cytoplasm	other
STAMBPL1	1.88E-04	1.527	STAM binding protein-like 1	unknown	other
STAR	9.14E-03	2.185	steroidogenic acute regulatory protein	Cytoplasm	transporter
STBD1	4.19E-05	−1.679	starch binding domain 1	Cytoplasm	other
STC2	7.76E-04	1.733	stanniocalcin 2	Extracellular Space	other
STOX2	1.56E-03	1.679	storkhead box 2	unknown	other
STRC	2.86E-02	1.708	stereocilin	Extracellular Space	other
STS	5.30E-03	3.513	steroid sulfatase (microsomal), isozyme S	Cytoplasm	enzyme
STX4	8.89E-03	1.892	syntaxin 4	Plasma Membrane	transporter
STX17	3.99E-05	3.195	syntaxin 17	Plasma Membrane	other
STXBP3	4.42E-02	−1.854	syntaxin binding protein 3	Plasma Membrane	transporter
SUFU	1.39E-02	−1.67	suppressor of fused homolog (Drosophila)	Nucleus	transcription regulator
SYBU	8.84E-06	1.896	syntabulin (syntaxin-interacting)	unknown	other
SYN1	1.20E-02	−1.558	synapsin I	Plasma Membrane	transporter
SYNGR3	1.25E-02	2.262	synaptogyrin 3	Plasma Membrane	other
SYT9	3.24E-03	1.677	synaptotagmin IX	Plasma Membrane	transporter
SYT11	5.33E-04	1.975	synaptotagmin XI	Cytoplasm	transporter
SYT14	4.21E-02	−3.007	synaptotagmin XIV	unknown	transporter
SZT2	2.66E-04	2.709	seizure threshold 2 homolog (mouse)	unknown	other
TAB1	1.50E-02	1.728	TGF-beta activated kinase 1/MAP3K7 binding protein 1	Cytoplasm	enzyme

TAGLN3	3.70E-04	1.752	transgelin 3	Extracellular Space	other
TBC1D10C	2.32E-02	−1.563	TBC1 domain family, member 10C	unknown	other
TCF3	3.24E-02	−1.752	transcription factor 3 (E2A immunoglobulin enhancer binding factors E12/E47)	Nucleus	transcription regulator
TCF21	4.84E-02	−1.948	transcription factor 21	Nucleus	transcription regulator
TDO2	4.14E-02	−2.754	tryptophan 2,3-dioxygenase	Cytoplasm	enzyme
TEAD4	1.62E-02	1.516	TEA domain family member 4	Nucleus	transcription regulator
TGFBRAP1	2.38E-04	3.757	transforming growth factor, beta receptor associated protein 1	Cytoplasm	other
TGM2	2.66E-02	2.756	transglutaminase 2 (C polypeptide, protein-glutamine-gamma-glutamyltransferase)	Cytoplasm	enzyme
TH	1.57E-02	4.272	tyrosine hydroxylase	Cytoplasm	enzyme
THADA	3.56E-02	−3.503	thyroid adenoma associated	unknown	other
THAP6	9.86E-05	1.805	THAP domain containing 6	unknown	other
THSD4	1.48E-02	2.852	thrombospondin, type I, domain containing 4	unknown	other
TIGD2	1.39E-04	2.008	tigger transposable element derived 2	unknown	other
TIMD4	6.35E-03	2.675	T-cell immunoglobulin and mucin domain containing 4	Plasma Membrane	other
TIPARP-AS1	4.94E-02	2.113	TIPARP antisense RNA 1 (non-protein coding)	unknown	other
TJP2	1.36E-03	−1.716	tight junction protein 2 (zona occludens 2)	Plasma Membrane	kinase
TLL2	4.99E-05	−2.33	tolloid-like 2	Extracellular Space	peptidase
TM4SF1	1.00E-05	−1.578	transmembrane 4 L six family member 1	Plasma Membrane	other
TMC5	1.30E-02	−1.826	transmembrane channel-like 5	unknown	other
TMCC1	4.74E-03	−2.344	transmembrane and coiled-coil domain family 1	unknown	other
TMCC3	1.73E-06	−2.908	transmembrane and coiled-coil domain family 3	unknown	other
TMED5	2.36E-02	−1.756	transmembrane emp24 protein transport domain containing 5	Cytoplasm	other
TMED9	1.45E-02	1.828	transmembrane emp24 protein transport domain containing 9	Cytoplasm	transporter
TMEM31	1.63E-04	3.38	transmembrane protein 31	unknown	other
TMEM51	1.00E-04	−1.565	transmembrane protein 51	unknown	other
TMEM100	2.45E-02	1.541	transmembrane protein 100	unknown	other
TMEM139	6.18E-07	1.718	transmembrane protein 139	unknown	other

TMEM169	1.10E-04	−2.1	transmembrane protein 169	unknown	other
TMEM174	6.78E-03	1.644	transmembrane protein 174	unknown	other
TMEM217	3.29E-04	−1.938	transmembrane protein 217	unknown	other
TMOD1	2.97E-03	2.885	tropomodulin 1	Cytoplasm	enzyme
TMSB10/TMSB4X	4.70E-03	−2.399	thymosin beta 4, X-linked	Cytoplasm	other
TNFAIP2	3.23E-03	−2.303	tumor necrosis factor, alpha-induced protein 2	Extracellular Space	other
TNFAIP6	1.07E-03	−1.557	tumor necrosis factor, alpha-induced protein 6	Extracellular Space	other
TNFAIP8	3.59E-06	−2.371	tumor necrosis factor, alpha-induced protein 8	Cytoplasm	other
TNFRSF10C	1.96E-03	3.188	tumor necrosis factor receptor superfamily, member 10c, decoy without an intracellular domain	Plasma Membrane	transmembrane receptor
TNFSF10	2.38E-05	−2.06	tumor necrosis factor (ligand) superfamily, member 10	Extracellular Space	cytokine
TNP2	4.79E-05	−2.855	transition protein 2 (during histone to protamine replacement)	Nucleus	other
TP63	1.09E-02	1.845	tumor protein p63	Nucleus	transcription regulator
TP53I11	2.12E-03	1.554	tumor protein p53 inducible protein 11	unknown	other
TPGS1	5.06E-04	2.7	tubulin polyglutamylase complex subunit 1	Cytoplasm	other
TPPP	3.34E-02	-2.952	tubulin polymerization promoting protein	Cytoplasm	other
TPR	1.23E-03	4.574	translocated promoter region (to activated MET oncogene)	Nucleus	other
TPRXL	1.70E-05	−1.773	tetra-peptide repeat homeobox-like	unknown	other
TRADD	2.29E-02	−2.13	TNFRSF1A-associated via death domain	Cytoplasm	other
TRAF3IP1	1.46E-04	1.567	TNF receptor-associated factor 3 interacting protein 1	Cytoplasm	other
TRAF3IP3	4.65E-05	−2.71	TRAF3 interacting protein 3	unknown	other
TRAIP	2.66E-02	2.321	TRAF interacting protein	Cytoplasm	other
TRAPPC2P1	1.14E-02	−2.278	trafficking protein particle complex 2 pseudogene 1	Extracellular Space	other
TRDV2	3.40E-02	−3.473	T cell receptor delta variable 2	Plasma Membrane	other
TRIM10	4.40E-02	1.865	tripartite motif containing 10	Cytoplasm	other
TRIM31	4.64E-02	2.066	tripartite motif containing 31	unknown	other
TRIM62	1.34E-03	2.027	tripartite motif containing 62	unknown	other
TRIM72	3.31E-02	2.259	tripartite motif containing 72	Cytoplasm	other
TRPC4	9.69E-04	−5.226	transient receptor potential cation channel, subfamily C, member 4	Plasma Membrane	ion channel

TRPM7	2.75E-02	1.979	transient receptor potential cation channel, subfamily M, member 7	Plasma Membrane	kinase
TSC22D4	4.88E-04	−1.658	TSC22 domain family, member 4	Nucleus	transcription regulator
TTBK2	2.92E-03	3.659	tau tubulin kinase 2	unknown	kinase
TTC5	1.19E-04	1.694	tetratricopeptide repeat domain 5	unknown	other
TTC6	4.05E-02	2.174	tetratricopeptide repeat domain 6	unknown	other
TTC23L	2.04E-02	−2.377	tetratricopeptide repeat domain 23-like	unknown	other
TTC9C	1.69E-03	−3.411	tetratricopeptide repeat domain 9C	unknown	other
TTLL5	4.39E-02	−2.356	tubulin tyrosine ligase-like family, member 5	Cytoplasm	enzyme
TTLL13	1.32E-02	2.451	tubulin tyrosine ligase-like family, member 13	unknown	enzyme
TUBB2B	7.51E-04	2.762	tubulin, beta 2B class IIb	Cytoplasm	other
TUBE1	4.74E-06	1.902	tubulin, epsilon 1	Cytoplasm	other
TWIST1	2.12E-02	2.97	twist homolog 1 (Drosophila)	Nucleus	transcription regulator
TWISTNB	3.83E-04	1.671	TWIST neighbor	Nucleus	other
TXNL4A	5.61E-03	−2.534	thioredoxin-like 4A	Nucleus	enzyme
UBE2Z	9.22E-03	−2.135	ubiquitin-conjugating enzyme E2Z	unknown	other
UBE3A	1.56E-02	3.102	ubiquitin protein ligase E3A	Nucleus	enzyme
UMOD	2.19E-02	2.888	uromodulin	Extracellular Space	other
UNC119	7.36E-03	2.337	unc-119 homolog (C. elegans)	Cytoplasm	other
USP2	2.75E-02	−1.947	ubiquitin specific peptidase 2	Cytoplasm	peptidase
USP36	2.80E-04	−2.699	ubiquitin specific peptidase 36	Nucleus	peptidase
USP44	8.59E-04	3.565	ubiquitin specific peptidase 44	Nucleus	peptidase
USPL1	2.81E-05	2.486	ubiquitin specific peptidase like 1	unknown	other
VDR	8.60E-06	1.519	vitamin D (1,25- dihydroxyvitamin D3) receptor	Nucleus	ligand-dependent nuclear receptor
VEPH1	7.06E-03	−1.957	ventricular zone expressed PH domain homolog 1 (zebrafish)	Nucleus	other
VIL1	1.20E-02	2.816	villin 1	Cytoplasm	other
VIPR2	6.88E-03	2.235	vasoactive intestinal peptide receptor 2	Plasma Membrane	G-protein coupled receptor
VNN3	4.15E-03	−2.263	vanin 3	Extracellular Space	enzyme
VPS13C	1.65E-04	1.772	vacuolar protein sorting 13 homolog C (S. cerevisiae)	unknown	other
VPS33B	3.70E-04	1.657	vacuolar protein sorting 33 homolog B (yeast)	Cytoplasm	transporter
VRTN	3.54E-02	2.798	vertebrae development homolog	unknown	other

			(pig)		
VSX1	3.45E-02	2.999	visual system homeobox 1	Nucleus	transcription regulator
WBP2NL	3.60E-02	−3.163	WBP2 N-terminal like	Cytoplasm	other
WDR24	2.57E-02	1.9	WD repeat domain 24	unknown	other
WDR44	2.08E-02	2.065	WD repeat domain 44	Cytoplasm	other
WDR72	7.34E-05	2.589	WD repeat domain 72	unknown	other
WFS1	1.52E-02	2.418	Wolfram syndrome 1 (wolframin)	Cytoplasm	enzyme
WIPF1	4.76E-03	2.52	WAS/WASL interacting protein family, member 1	Cytoplasm	other
WNT2B	4.80E-02	−1.808	wingless-type MMTV integration site family, member 2B	Extracellular Space	other
WNT9A	7.96E-03	−1.629	wingless-type MMTV integration site family, member 9A	Extracellular Space	other
WSB1	6.80E-03	−1.958	WD repeat and SOCS box containing 1	unknown	other
WWC1	6.63E-04	−2.045	WW and C2 domain containing 1	Cytoplasm	transcription regulator
XRCC6BP1	5.05E-06	1.53	XRCC6 binding protein 1	unknown	kinase
XRRA1	2.16E-02	2.151	X-ray radiation resistance associated 1	Cytoplasm	other
XYLB	4.03E-02	2.111	xylulokinase homolog (H. influenzae)	unknown	kinase
YES1	1.81E-02	3.756	v-yes-1 Yamaguchi sarcoma viral oncogene homolog 1	Cytoplasm	kinase
YPEL2	6.99E-05	−1.603	yippee-like 2 (Drosophila)	Nucleus	other
YWHAH	8.21E-03	2.037	tyrosine 3-monooxygenase/tryptophan 5-monooxygenase activation protein, eta polypeptide	Cytoplasm	transcription regulator
ZBTB6	2.91E-03	−1.558	zinc finger and BTB domain containing 6	Nucleus	other
ZBTB44	1.49E-02	−2.482	zinc finger and BTB domain containing 44	unknown	other
ZBTB7A	2.08E-02	−1.709	zinc finger and BTB domain containing 7A	Nucleus	transcription regulator
ZBTB7C	3.33E-03	1.816	zinc finger and BTB domain containing 7C	unknown	other
ZC3H12B	2.64E-03	2.78	zinc finger CCCH-type containing 12B	unknown	other
ZC3H12C	4.32E-04	−1.922	zinc finger CCCH-type containing 12C	unknown	other
ZDHHC11	8.81E-03	−1.542	zinc finger, DHHC-type containing 11	unknown	other
ZER1	4.24E-02	−2.088	zer-1 homolog (C. elegans)	unknown	enzyme
ZFP82	7.87E-04	3.543	zinc finger protein 82 homolog	Nucleus	other

			(mouse)		
ZHX3	1.99E-03	1.581	zinc fingers and homeoboxes 3	Nucleus	transcription regulator
ZMYM3	3.13E-03	2.237	zinc finger, MYM-type 3	Nucleus	other
ZMYM6	4.87E-02	−2.996	zinc finger, MYM-type 6	Nucleus	other
ZMYND15	9.26E-03	−3.26	zinc finger, MYND-type containing 15	unknown	other
ZNF19	3.01E-02	2.102	zinc finger protein 19	Nucleus	transcription regulator
ZNF20	3.47E-03	1.803	zinc finger protein 20	Nucleus	other
ZNF23	3.72E-02	1.745	zinc finger protein 23 (KOX 16)	Nucleus	other
ZNF90	2.55E-02	2.754	zinc finger protein 90	Cytoplasm	transcription regulator
ZNF167	1.10E-02	2.151	zinc finger protein 167	Nucleus	transcription regulator
ZNF230	1.51E-02	−3.275	zinc finger protein 230	Nucleus	other
ZNF235	3.76E-02	2.351	zinc finger protein 235	Nucleus	other
ZNF248	3.01E-02	1.626	zinc finger protein 248	Nucleus	other
ZNF273	1.89E-02	1.625	zinc finger protein 273	Nucleus	other
ZNF346	3.66E-03	2.594	zinc finger protein 346	Nucleus	other
ZNF365	2.21E-05	−1.938	zinc finger protein 365	Cytoplasm	other
ZNF395	7.22E-05	4.122	zinc finger protein 395	Cytoplasm	other
ZNF396	4.71E-02	2.666	zinc finger protein 396	Nucleus	transcription regulator
ZNF420	2.48E-02	2.194	zinc finger protein 420	unknown	other
ZNF461	2.73E-02	−1.58	zinc finger protein 461	Nucleus	other
ZNF462	5.48E-03	1.575	zinc finger protein 462	Nucleus	other
ZNF500	8.18E-03	1.849	zinc finger protein 500	Nucleus	other
ZNF555	5.58E-04	2.214	zinc finger protein 555	unknown	other
ZNF557	3.43E-02	−3.544	zinc finger protein 557	Nucleus	other
ZNF564	4.37E-03	−1.814	zinc finger protein 564	Nucleus	other
ZNF570	5.23E-03	1.545	zinc finger protein 570	Nucleus	other
ZNF643	2.75E-05	2.91	zinc finger protein 643	Nucleus	other
ZNF683	3.71E-02	2.081	zinc finger protein 683	unknown	other
ZNF707	3.59E-02	2.156	zinc finger protein 707	unknown	other
ZNF710	7.70E-03	−2.243	zinc finger protein 710	Nucleus	other
ZNF749	1.13E-02	3.256	zinc finger protein 749	unknown	other
ZNF750	8.01E-07	−2.415	zinc finger protein 750	unknown	other
ZNF763	2.87E-03	1.944	zinc finger protein 763	unknown	other
ZNF788	5.38E-04	3.694	zinc finger family member 788	unknown	other
ZNF839	3.02E-04	3.319	zinc finger protein 839	unknown	other
ZNF324/ZNF324B	2.10E-03	−4.197	zinc finger protein 324	Nucleus	other
ZNF585B	3.60E-02	2.456	zinc finger protein 585B	Nucleus	other
ZNF75A	2.68E-02	1.643	zinc finger protein 75a	Nucleus	other
ZNF780A/ZNF	3.42E-02	−3.351	zinc finger protein 780A	unknown	other

780B					
ZNFX1	6.92E-03	−1.859	zinc finger, NFX1-type containing 1	Nucleus	transcription regulator
ZNHIT2	4.23E-04	2.261	zinc finger, HIT-type containing 2	unknown	other
ZP1	3.30E-02	2.178	zona pellucida glycoprotein 1 (sperm receptor)	Extracellular Space	other
ZSCAN20	2.47E-02	−2.494	zinc finger and SCAN domain containing 20	Nucleus	other

Supplementary Table 3. 1142 genes are unique to 24 h post-heat timepoint. The name, p-value, fold change, location and family of each gene are indicated. Genes were filtered for an absolute value log2 ration ≥1.5 and a significance value of $p \leq 0.05$.

**Symbol**	**p-value**	**Log Ratio**	**Gene Name**	**Location**	**Family**
AADACL2	1.24E-04	−2.103	arylacetamide deacetylase-like 2	unknown	other
ABCB11	3.91E-02	3.288	ATP-binding cassette, sub-family B (MDR/TAP), member 11	Extracellular Space	transporter
ABCC9	5.10E-03	3.62	ATP-binding cassette, sub-family C (CFTR/MRP), member 9	Plasma Membrane	ion channel
ABCC4 (includes EG:10257)	5.08E-05	1.752	ATP-binding cassette, sub-family C (CFTR/MRP), member 4	Plasma Membrane	transporter
ABCE1	9.53E-07	1.514	ATP-binding cassette, sub-family E (OABP), member 1	Cytoplasm	transporter
ABCF2	3.10E-04	1.612	ATP-binding cassette, sub-family F (GCN20), member 2	Cytoplasm	transporter
ACCN4	1.29E-05	2.669	amiloride-sensitive cation channel 4, pituitary	Plasma Membrane	ion channel
ACE2 (includes EG:302668)	2.73E-02	1.614	angiotensin I converting enzyme (peptidyl-dipeptidase A) 2	Plasma Membrane	peptidase
ACER1	1.01E-02	-3.207	alkaline ceramidase 1	Cytoplasm	enzyme
ACER3	1.25E-04	2.926	alkaline ceramidase 3	Cytoplasm	enzyme
ACN9 (includes EG:362323)	6.33E-04	1.754	ACN9 homolog (S. cerevisiae)	Cytoplasm	other
ACOT6	1.66E-02	−2.759	acyl-CoA thioesterase 6	Cytoplasm	enzyme
ACPP	4.43E-04	−2.211	acid phosphatase, prostate	Extracellular Space	phosphatase
ACTB	1.06E-02	1.58	actin, beta	Cytoplasm	other
ACTG2	1.41E-02	−2.214	actin, gamma 2, smooth muscle, enteric	Cytoplasm	other
ACY1	1.04E-06	1.693	aminoacylase 1	Cytoplasm	peptidase
ACYP1	2.82E-04	1.515	acylphosphatase 1, erythrocyte (common) type	unknown	enzyme
ADAM8	2.42E-03	1.525	ADAM metallopeptidase domain 8	Plasma Membrane	peptidase
ADAM30	3.23E-03	3.687	ADAM metallopeptidase domain 30	Plasma Membrane	peptidase
ADAP2	1.90E-04	−1.592	ArfGAP with dual PH domains 2	Cytoplasm	other

ADC	3.72E-03	2.321	arginine decarboxylase	Cytoplasm	enzyme
ADCY7	1.01E-03	−1.814	adenylate cyclase 7	Plasma Membrane	enzyme
ADM2	1.18E-03	1.873	adrenomedullin 2	Extracellular Space	other
ADORA2B	9.20E-06	1.681	adenosine A2b receptor	Plasma Membrane	G-protein coupled receptor
ADSL	2.34E-04	1.737	adenylosuccinate lyase	Cytoplasm	enzyme
AFP	1.69E-05	3.315	alpha-fetoprotein	Extracellular Space	transporter
AIF1 (includes EG:11629)	3.21E-02	1.636	allograft inflammatory factor 1	Nucleus	other
AKT1	7.62E-03	1.56	v-akt murine thymoma viral oncogene homolog 1	Cytoplasm	kinase
ALDH1B1	4.30E-05	1.695	aldehyde dehydrogenase 1 family, member B1	Cytoplasm	enzyme
ALDH1L2	3.02E-03	1.682	aldehyde dehydrogenase 1 family, member L2	Cytoplasm	enzyme
ALDH3A1	2.39E-02	1.611	aldehyde dehydrogenase 3 family, member A1	Cytoplasm	enzyme
ALG10B	4.70E-05	1.943	asparagine-linked glycosylation 10, alpha-1,2-glucosyltransferase homolog B (yeast)	Plasma Membrane	transporter
ALOX5	2.84E-02	−2.455	arachidonate 5-lipoxygenase	Cytoplasm	enzyme
ALOX12B	6.05E-03	−1.557	arachidonate 12-lipoxygenase, 12R type	unknown	enzyme
ALOX15B	2.03E-03	2.039	arachidonate 15-lipoxygenase, type B	Cytoplasm	enzyme
AMOT	1.54E-02	2.491	angiomotin	Plasma Membrane	other
ANAPC10	1.45E-04	1.62	anaphase promoting complex subunit 10	Nucleus	enzyme
ANGPT1	4.31E-04	3.7	angiopoietin 1	Extracellular Space	growth factor
ANK3	3.09E-05	−1.653	ankyrin 3, node of Ranvier (ankyrin G)	Plasma Membrane	other
ANKMY1	2.78E-02	2.892	ankyrin repeat and MYND domain containing 1	unknown	other
ANKRD5	1.72E-04	2.027	ankyrin repeat domain 5	Nucleus	transcription regulator
ANKRD6	2.54E-04	1.844	ankyrin repeat domain 6	Nucleus	transcription regulator
ANKRD35	3.58E-04	−1.991	ankyrin repeat domain 35	unknown	other
ANKRD40	4.74E-03	1.603	ankyrin repeat domain 40	unknown	other
ANKRD50	3.44E-02	1.527	ankyrin repeat domain 50	unknown	other

ANKRD53	3.16E-02	1.811	ankyrin repeat domain 53	Nucleus	transcription regulator
ANKRD55	1.60E-02	1.717	ankyrin repeat domain 55	Nucleus	transcription regulator
ANLN	9.13E-06	1.705	anillin, actin binding protein	Cytoplasm	other
ANO1	1.00E-02	1.727	anoctamin 1, calcium activated chloride channel	Plasma Membrane	ion channel
ANO4	4.73E-02	1.848	anoctamin 4	unknown	other
ANTXR2	2.12E-02	2.07	anthrax toxin receptor 2	Plasma Membrane	other
ANXA6	2.09E-06	2.19	annexin A6	Plasma Membrane	other
AP1M1	1.21E-03	2.803	adaptor-related protein complex 1, mu 1 subunit	Cytoplasm	transporter
AP1S1	2.00E-02	1.569	adaptor-related protein complex 1, sigma 1 subunit	Cytoplasm	transporter
APEH	1.91E-05	1.512	N-acylaminoacyl-peptide hydrolase	Cytoplasm	peptidase
APH1A	3.48E-02	3.662	anterior pharynx defective 1 homolog A (C. elegans)	Cytoplasm	peptidase
APLNR	3.11E-02	1.737	apelin receptor	Plasma Membrane	G-protein coupled receptor
APLP1	3.31E-02	−1.657	amyloid beta (A4) precursor-like protein 1	Extracellular Space	other
APOLD1	6.03E-06	1.651	apolipoprotein L domain containing 1	unknown	other
AQP5	5.20E-04	−3.079	aquaporin 5	Plasma Membrane	transporter
ARFRP1	2.75E-05	3.729	ADP-ribosylation factor related protein 1	Cytoplasm	enzyme
ARG1	4.24E-03	−2.223	arginase, liver	Cytoplasm	enzyme
ARGLU1	1.40E-02	1.97	arginine and glutamate rich 1	unknown	other
ARHGAP18	7.31E-03	1.783	Rho GTPase activating protein 18	Cytoplasm	other
ARHGAP19	7.83E-04	2.349	Rho GTPase activating protein 19	Cytoplasm	other
ARHGAP28	2.19E-02	−2.47	Rho GTPase activating protein 28	Cytoplasm	other
ARHGDIA	5.75E-04	1.617	Rho GDP dissociation inhibitor (GDI) alpha	Cytoplasm	other
ARHGDIB	3.10E-02	1.754	Rho GDP dissociation inhibitor (GDI) beta	Cytoplasm	other
ARHGEF12	3.91E-03	2.353	Rho guanine nucleotide exchange factor (GEF) 12	Cytoplasm	other
ARID1A	2.83E-03	3.724	AT rich interactive domain 1A (SWI-like)	Nucleus	transcription regulator
ARL9	1.06E-02	2.103	ADP-ribosylation factor-like 9	unknown	other
ARL5A	1.08E-04	−1.667	ADP-ribosylation factor-like 5A	unknown	enzyme

ARMC9	3.58E-04	1.645	armadillo repeat containing 9	unknown	other
ARNT2	2.16E-03	3.681	aryl-hydrocarbon receptor nuclear translocator 2	Nucleus	transcription regulator
ASB4	3.22E-02	3.567	ankyrin repeat and SOCS box containing 4	Nucleus	transcription regulator
ASB9	1.44E-03	3.352	ankyrin repeat and SOCS box containing 9	Nucleus	transcription regulator
ASPM	5.25E-06	1.831	asp (abnormal spindle) homolog, microcephaly associated (Drosophila)	Nucleus	other
ASPN	5.30E-05	2.299	asporin	Extracellular Space	other
ATCAY	3.99E-02	1.561	ataxia, cerebellar, Cayman type	unknown	other
ATG10 (includes EG:641330)	1.37E-03	1.942	ATG10 autophagy related 10 homolog (S. cerevisiae)	Plasma Membrane	enzyme
ATP4A	3.90E-02	3.212	ATPase, H+/K+ exchanging, alpha polypeptide	Plasma Membrane	transporter
ATP6V1C2	5.45E-03	−1.732	ATPase, H+ transporting, lysosomal 42kDa, V1 subunit C2	Cytoplasm	transporter
ATP8A2	2.69E-03	1.903	ATPase, aminophospholipid transporter, class I, type 8A, member 2	Plasma Membrane	transporter
ATP8B2	2.73E-03	2.171	ATPase, class I, type 8B, member 2	Plasma Membrane	transporter
ATXN2L	1.06E-02	1.751	ataxin 2-like	unknown	other
AURKA	2.82E-07	1.847	aurora kinase A	Nucleus	kinase
AURKB	2.77E-05	3.239	aurora kinase B	Nucleus	kinase
AVPR1B	3.75E-02	1.773	arginine vasopressin receptor 1B	Plasma Membrane	G-protein coupled receptor
B4GALT7	5.40E-05	3.598	xylosylprotein beta 1,4-galactosyltransferase, polypeptide 7 (galactosyltransferase I)	Cytoplasm	enzyme
BACE1	1.32E-03	1.86	beta-site APP-cleaving enzyme 1	Cytoplasm	peptidase
BCL6	2.28E-04	−1.558	B-cell CLL/lymphoma 6	Nucleus	transcription regulator
BCL10	5.79E-04	1.663	B-cell CLL/lymphoma 10	Cytoplasm	transcription regulator
BCL11B	8.19E-04	3.182	B-cell CLL/lymphoma 11B (zinc finger protein)	Nucleus	other
BEX1	2.23E-06	1.567	brain expressed, X-linked 1	Cytoplasm	other
BIRC5	1.37E-04	2.113	baculoviral IAP repeat containing 5	Cytoplasm	other
BIVM	8.86E-03	2.123	basic, immunoglobulin-like variable motif containing	unknown	other
BLK	3.01E-02	−2.169	B lymphoid tyrosine kinase	Cytoplasm	kinase

BLVRA	9.71E-04	2.701	biliverdin reductase A	Cytoplasm	enzyme
BMPR1B	6.37E-04	2.881	bone morphogenetic protein receptor, type IB	Plasma Membrane	kinase
BOLA3	3.20E-06	2.27	bolA homolog 3 (E. coli)	unknown	other
BPIFC	2.79E-04	−1.912	BPI fold containing family C	Extracellular Space	transporter
BRCA2	5.03E-03	1.51	breast cancer 2, early onset	Nucleus	transcription regulator
BTK	1.68E-02	2.36	Bruton agammaglobulinemia tyrosine kinase	Cytoplasm	kinase
BUB1 (includes EG:100307076)	4.29E-03	1.829	budding uninhibited by benzimidazoles 1 homolog (yeast)	Nucleus	kinase
BVES	8.50E-03	2.317	blood vessel epicardial substance	Plasma Membrane	other
C5	3.34E-03	1.921	complement component 5	Extracellular Space	cytokine
C10orf99	1.38E-06	−2.276	chromosome 10 open reading frame 99	unknown	other
C10orf136	2.54E-02	3.058	chromosome 10 open reading frame 136	unknown	other
C11orf24	2.03E-04	1.619	chromosome 11 open reading frame 24	Extracellular Space	other
C12orf24	1.08E-05	1.597	chromosome 12 open reading frame 24	unknown	other
C12orf37	2.48E-02	−2.318	chromosome 12 open reading frame 37	unknown	other
C12orf66	7.89E-03	1.714	chromosome 12 open reading frame 66	unknown	other
C15orf63	7.34E-05	1.528	chromosome 15 open reading frame 63	unknown	other
C16orf74	5.73E-05	2.811	chromosome 16 open reading frame 74	unknown	other
C19orf57	3.67E-02	−2.099	chromosome 19 open reading frame 57	unknown	other
C1orf52	1.31E-02	1.849	chromosome 1 open reading frame 52	unknown	other
C1orf94	1.03E-02	1.517	chromosome 1 open reading frame 94	unknown	other
C1orf112	2.04E-04	4.34	chromosome 1 open reading frame 112	unknown	other
C1orf144	4.71E-03	1.987	chromosome 1 open reading frame 144	unknown	other
C20orf27	2.68E-02	2.395	chromosome 20 open reading frame 27	unknown	other
C20orf30	7.03E-05	1.53	chromosome 20 open reading frame 30	unknown	other

C21orf15	1.09E-02	2.551	chromosome 21 open reading frame 15	unknown	other
C22orf39	1.06E-04	1.54	chromosome 22 open reading frame 39	unknown	other
C2orf88	2.22E-02	−1.928	chromosome 2 open reading frame 88	unknown	other
C3orf26	1.60E-04	2.204	chromosome 3 open reading frame 26	unknown	other
C3orf65	9.09E-03	2.739	chromosome 3 open reading frame 65	unknown	other
C3orf78	1.94E-08	4.138	chromosome 3 open reading frame 78	Cytoplasm	other
C3orf80	3.66E-02	3.13	chromosome 3 open reading frame 80	unknown	other
C5orf4	7.65E-04	−1.679	chromosome 5 open reading frame 4	unknown	other
C5orf39	2.41E-02	2.113	chromosome 5 open reading frame 39	unknown	other
C5orf56	1.18E-04	−2.041	chromosome 5 open reading frame 56	unknown	other
C6orf64	2.83E-02	−2.328	chromosome 6 open reading frame 64	unknown	other
C6orf108	7.65E-03	2.852	chromosome 6 open reading frame 108	Nucleus	other
C6orf118	1.55E-02	−2.015	chromosome 6 open reading frame 118	unknown	other
C7orf45	3.16E-03	3.15	chromosome 7 open reading frame 45	unknown	other
C8orf4	3.83E-02	4.968	chromosome 8 open reading frame 4	unknown	other
C8orf84	1.64E-04	−1.968	chromosome 8 open reading frame 84	Extracellular Space	other
C9orf9	2.59E-02	3.096	chromosome 9 open reading frame 9	unknown	other
C9orf46	1.75E-06	1.51	chromosome 9 open reading frame 46	Cytoplasm	other
C9orf131	2.09E-02	−2.582	chromosome 9 open reading frame 131	unknown	other
C9orf142	4.72E-02	1.877	chromosome 9 open reading frame 142	unknown	other
CA5BP1	5.53E-06	3.902	carbonic anhydrase VB pseudogene 1	unknown	other
CABP5	1.47E-02	−2.156	calcium binding protein 5	Cytoplasm	other
CACNA1E	3.10E-02	2.771	calcium channel, voltage-dependent, R type, alpha 1E subunit	Plasma Membrane	ion channel

CACNA1I	1.07E-03	2.835	calcium channel, voltage-dependent, T type, alpha 1I subunit	Plasma Membrane	ion channel
CADM3	2.89E-02	−1.809	cell adhesion molecule 3	Plasma Membrane	other
CADPS	4.45E-02	−2.46	Ca++-dependent secretion activator	Plasma Membrane	other
CASC4	1.26E-02	2.227	cancer susceptibility candidate 4	unknown	other
CASK	4.38E-02	3.02	calcium/calmodulin-dependent serine protein kinase (MAGUK family)	Plasma Membrane	kinase
CASKIN1	1.11E-02	−1.972	CASK interacting protein 1	Nucleus	transcription regulator
CASP1	3.89E-05	1.607	caspase 1, apoptosis-related cysteine peptidase (interleukin 1, beta, convertase)	Cytoplasm	peptidase
CASP3	1.90E-03	2.854	caspase 3, apoptosis-related cysteine peptidase	Cytoplasm	peptidase
CASP5	4.46E-05	2.647	caspase 5, apoptosis-related cysteine peptidase	Cytoplasm	peptidase
CASP10	1.80E-03	1.895	caspase 10, apoptosis-related cysteine peptidase	Cytoplasm	peptidase
CBLC	4.88E-03	−2.054	Cas-Br-M (murine) ecotropic retroviral transforming sequence c	Nucleus	enzyme
CBR1	8.50E-04	1.548	carbonyl reductase 1	Cytoplasm	enzyme
CBX6	2.91E-03	1.546	chromobox homolog 6	Nucleus	other
CCDC134	2.85E-02	−2.062	coiled-coil domain containing 134	unknown	other
CCNB1	8.28E-04	1.527	cyclin B1	Cytoplasm	other
CCND2	1.66E-05	1.817	cyclin D2	Nucleus	other
CCND3	3.64E-03	3.008	cyclin D3	Nucleus	other
CCNF	5.57E-03	−2.049	cyclin F	Nucleus	other
CCNT1	1.26E-02	2.83	cyclin T1	Nucleus	transcription regulator
CCR2	2.09E-02	−2.858	chemokine (C-C motif) receptor 2	Plasma Membrane	G-protein coupled receptor
CCT2	2.02E-03	2.21	chaperonin containing TCP1, subunit 2 (beta)	Cytoplasm	kinase
CD2	3.49E-02	1.524	CD2 molecule	Plasma Membrane	transmembrane receptor
CD14	3.13E-02	−2.933	CD14 molecule	Plasma Membrane	transmembrane receptor
CD33	2.28E-02	2.114	CD33 molecule	Plasma Membrane	other
CD244	4.37E-02	2.129	CD244 molecule, natural killer cell receptor 2B4	Plasma Membrane	other

CD247	2.53E-03	−2.152	CD247 molecule	Plasma Membrane	transmembrane receptor
CD2AP	1.14E-03	1.629	CD2-associated protein	Cytoplasm	other
CD44 (includes EG:100330801)	1.62E-03	1.71	CD44 molecule (Indian blood group)	Plasma Membrane	other
CDC25C	9.58E-04	2.316	cell division cycle 25 homolog C (S. pombe)	Nucleus	phosphatase
CDC42BPG	1.63E-03	2.328	CDC42 binding protein kinase gamma (DMPK-like)	Cytoplasm	kinase
CDCA8	1.66E-04	2.305	cell division cycle associated 8	Nucleus	other
CDCP1	4.21E-04	1.648	CUB domain containing protein 1	Plasma Membrane	other
CDHR5	6.36E-04	−2.7	cadherin-related family member 5	Plasma Membrane	other
CDK1	3.68E-06	2.015	cyclin-dependent kinase 1	Nucleus	kinase
CDK4	1.64E-07	1.638	cyclin-dependent kinase 4	Nucleus	kinase
CDK10	2.42E-04	1.899	cyclin-dependent kinase 10	Nucleus	kinase
CDKN3	6.89E-06	1.873	cyclin-dependent kinase inhibitor 3	Nucleus	phosphatase
CDKN1A	3.14E-02	2.388	cyclin-dependent kinase inhibitor 1A (p21, Cip1)	Nucleus	kinase
CECR9	2.99E-02	2.268	cat eye syndrome chromosome region, candidate 9 (non-protein coding)	unknown	other
CENPE	6.81E-06	1.568	centromere protein E, 312kDa	Nucleus	other
CENPO	2.91E-02	2.029	centromere protein O	unknown	other
CENPV	7.46E-03	1.645	centromere protein V	Nucleus	other
CENPW	3.85E-06	1.613	centromere protein W	unknown	other
CEP55	2.68E-05	1.56	centrosomal protein 55kDa	Cytoplasm	other
CEP63	1.51E-03	3.309	centrosomal protein 63kDa	Cytoplasm	other
CEP85	1.05E-03	2.724	centrosomal protein 85kDa	Nucleus	other
CEP89	4.99E-03	4.821	centrosomal protein 89kDa	unknown	other
CERS3	1.01E-05	−1.755	ceramide synthase 3	Nucleus	transcription regulator
CFHR5	2.01E-02	−2.949	complement factor H-related 5	Extracellular Space	other
CHCHD8	1.93E-07	2.457	coiled-coil-helix-coiled-coil-helix domain containing 8	unknown	other
CHGA	4.13E-02	2.025	chromogranin A (parathyroid secretory protein 1)	Extracellular Space	other
CHI3L1	4.78E-02	−1.628	chitinase 3-like 1 (cartilage glycoprotein-39)	Extracellular Space	enzyme
CHIA	3.84E-02	−1.715	chitinase, acidic	Extracellular Space	enzyme
CHIT1	2.96E-02	−1.972	chitinase 1 (chitotriosidase)	Extracellular Space	enzyme

CHML	8.88E-03	1.528	choroideremia-like (Rab escort protein 2)	Cytoplasm	enzyme
CHN2	5.62E-06	2.055	chimerin (chimaerin) 2	Cytoplasm	other
CHODL	2.06E-04	1.915	chondrolectin	Plasma Membrane	other
CHRM1	4.48E-02	1.856	cholinergic receptor, muscarinic 1	Plasma Membrane	G-protein coupled receptor
CHRNA4	4.83E-02	1.628	cholinergic receptor, nicotinic, alpha 4	Plasma Membrane	transmembrane receptor
CHST9-AS1	6.56E-03	1.538	CHST9 antisense RNA 1 (non-protein coding)	unknown	other
CIB4	2.14E-02	−2.255	calcium and integrin binding family member 4	unknown	other
CIRH1A	3.80E-06	2.469	cirrhosis, autosomal recessive 1A (cirhin)	Nucleus	other
CKAP2L	1.05E-04	−1.952	cytoskeleton associated protein 2-like	Cytoplasm	other
CLCN1	3.14E-03	2.731	chloride channel 1, skeletal muscle	Plasma Membrane	ion channel
CLDN16	2.14E-03	2.37	claudin 16	Plasma Membrane	transporter
CLEC2B	9.95E-05	3.616	C-type lectin domain family 2, member B	Plasma Membrane	other
CLN6	1.11E-05	3.373	ceroid-lipofuscinosis, neuronal 6, late infantile, variant	Cytoplasm	other
CLPTM1L	1.61E-02	2.208	CLPTM1-like	unknown	other
CLRN1	5.03E-04	−1.864	clarin 1	unknown	other
CLSPN	2.97E-03	2.234	claspin	Nucleus	other
CLVS1	9.57E-03	4.043	clavesin 1	Cytoplasm	other
CMTM3	1.74E-03	1.97	CKLF-like MARVEL transmembrane domain containing 3	Extracellular Space	cytokine
CMTM4	1.22E-02	2.581	CKLF-like MARVEL transmembrane domain containing 4	Extracellular Space	cytokine
CNTD2	1.31E-02	−1.581	cyclin N-terminal domain containing 2	unknown	other
CNTF	2.03E-03	2.24	ciliary neurotrophic factor	Extracellular Space	cytokine
COL21A1	8.44E-03	4.597	collagen, type XXI, alpha 1	Extracellular Space	other
COL24A1	3.70E-02	2.919	collagen, type XXIV, alpha 1	Extracellular Space	other
COL8A1	3.13E-07	4.941	collagen, type VIII, alpha 1	Extracellular Space	other

COPA	1.23E-03	2.482	coatomer protein complex, subunit alpha	Cytoplasm	transporter
COQ7	3.58E-02	2.003	coenzyme Q7 homolog, ubiquinone (yeast)	Cytoplasm	other
CORIN	7.30E-03	−3.714	corin, serine peptidase	Plasma Membrane	peptidase
CPLX1	2.26E-02	−2.16	complexin 1	Plasma Membrane	transporter
CPSF6	1.21E-03	1.651	cleavage and polyadenylation specific factor 6, 68kDa	Nucleus	other
CPVL	2.06E-03	2.69	carboxypeptidase, vitellogenic-like	unknown	peptidase
CREM	1.79E-02	2.224	cAMP responsive element modulator	Nucleus	other
CRISPLD2	8.63E-04	−1.6	cysteine-rich secretory protein LCCL domain containing 2	Cytoplasm	other
CSF2	3.07E-04	2.599	colony stimulating factor 2 (granulocyte-macrophage)	Extracellular Space	cytokine
CSMD1	3.15E-02	3.988	CUB and Sushi multiple domains 1	Plasma Membrane	other
CSNK1G1	3.40E-02	2.396	casein kinase 1, gamma 1	Cytoplasm	kinase
CSRNP3	3.91E-02	4.971	cysteine-serine-rich nuclear protein 3	Nucleus	transcription regulator
CTIF	2.82E-03	2.467	CBP80/20-dependent translation initiation factor	Cytoplasm	other
CTSH	4.71E-05	−2.19	cathepsin H	Cytoplasm	peptidase
CTSZ	5.80E-04	1.661	cathepsin Z	Cytoplasm	peptidase
CUEDC1	5.74E-03	3.133	CUE domain containing 1	unknown	other
CUL4A	1.15E-02	1.634	cullin 4A	Nucleus	other
CWH43	4.76E-06	−1.759	cell wall biogenesis 43 C-terminal homolog (S. cerevisiae)	unknown	other
CXCL1	9.34E-06	1.832	chemokine (C-X-C motif) ligand 1 (melanoma growth stimulating activity, alpha)	Extracellular Space	cytokine
CXCL3	5.61E-06	2.124	chemokine (C-X-C motif) ligand 3	Extracellular Space	cytokine
CXCL5	6.80E-06	1.819	chemokine (C-X-C motif) ligand 5	Extracellular Space	cytokine
CXCR3	1.34E-03	3.518	chemokine (C-X-C motif) receptor 3	Plasma Membrane	G-protein coupled receptor
CXCR4	2.72E-02	2.019	chemokine (C-X-C motif) receptor 4	Plasma Membrane	G-protein coupled receptor
CXorf58	2.89E-02	4.078	chromosome X open reading frame 58	unknown	other

CXorf65	1.30E-02	−1.973	chromosome X open reading frame 65	unknown	other
CYP11B2	7.35E-03	−2.243	cytochrome P450, family 11, subfamily B, polypeptide 2	Cytoplasm	enzyme
CYP1A2	3.35E-02	−2.299	cytochrome P450, family 1, subfamily A, polypeptide 2	Cytoplasm	enzyme
CYP2A6 (includes others)	4.98E-03	−1.853	cytochrome P450, family 2, subfamily A, polypeptide 6	Cytoplasm	enzyme
CYP2W1	9.00E-03	−1.523	cytochrome P450, family 2, subfamily W, polypeptide 1	Cytoplasm	enzyme
CYP3A4	3.37E-02	1.6	cytochrome P450, family 3, subfamily A, polypeptide 4	Cytoplasm	enzyme
CYP4B1	3.11E-04	−1.799	cytochrome P450, family 4, subfamily B, polypeptide 1	Cytoplasm	enzyme
CYTH4	2.55E-02	2.386	cytohesin 4	Cytoplasm	other
DAAM1	1.27E-02	−1.503	dishevelled associated activator of morphogenesis 1	Cytoplasm	other
DCBLD1	6.16E-05	−1.61	discoidin, CUB and LCCL domain containing 1	Extracellular Space	other
DCPS	1.95E-04	1.697	decapping enzyme, scavenger	Nucleus	enzyme
DCX	2.73E-02	3.218	doublecortin	Cytoplasm	other
DDX17	1.44E-04	1.868	DEAD (Asp-Glu-Ala-Asp) box polypeptide 17	Nucleus	enzyme
DDX42	7.53E-03	1.542	DEAD (Asp-Glu-Ala-Asp) box polypeptide 42	Cytoplasm	enzyme
DDX43	4.87E-03	1.796	DEAD (Asp-Glu-Ala-Asp) box polypeptide 43	unknown	enzyme
DDX46	2.58E-02	2.469	DEAD (Asp-Glu-Ala-Asp) box polypeptide 46	Nucleus	enzyme
DEF6	6.18E-03	1.974	differentially expressed in FDCP 6 homolog (mouse)	Extracellular Space	other
DEFA1 (includes others)	1.65E-02	3.013	defensin, alpha 1	Extracellular Space	other
DEPDC1	1.17E-04	2.028	DEP domain containing 1	Nucleus	transcription regulator
DGCR10	1.90E-02	−1.54	DiGeorge syndrome critical region gene 10	unknown	other
DHCR24	1.56E-06	−1.557	24-dehydrocholesterol reductase	Cytoplasm	enzyme
DHDDS	2.85E-02	1.843	dehydrodolichyl diphosphate synthase	unknown	enzyme
DHPS	9.72E-04	1.83	deoxyhypusine synthase	Cytoplasm	enzyme
DHRS2	3.01E-02	1.87	dehydrogenase/reductase (SDR family) member 2	Nucleus	enzyme
DHRS9	6.78E-04	1.618	dehydrogenase/reductase (SDR family) member 9	Cytoplasm	enzyme
DHX9	2.65E-04	3.341	DEAH (Asp-Glu-Ala-His) box	Nucleus	enzyme

			polypeptide 9		
DHX30	1.69E-02	2.763	DEAH (Asp-Glu-Ala-His) box polypeptide 30	Nucleus	enzyme
DIMT1	1.27E-06	1.517	DIM1 dimethyladenosine transferase 1 homolog (S. cerevisiae)	Cytoplasm	enzyme
DKC1	2.29E-05	3.928	dyskeratosis congenita 1, dyskerin	Nucleus	enzyme
DLC1	1.32E-03	2.774	deleted in liver cancer 1	Cytoplasm	other
DLGAP5	1.41E-04	2.44	discs, large (Drosophila) homolog-associated protein 5	Nucleus	phosphatase
DLL3	4.73E-02	2.008	delta-like 3 (Drosophila)	Extracellular Space	other
DLX5	1.14E-03	−1.88	distal-less homeobox 5	Nucleus	transcription regulator
DMC1	3.27E-02	1.691	DMC1 dosage suppressor of mck1 homolog, meiosis-specific homologous recombination (yeast)	Nucleus	enzyme
DNAH2	4.00E-03	4.006	dynein, axonemal, heavy chain 2	unknown	other
DNAH10	3.15E-02	2.216	dynein, axonemal, heavy chain 10	unknown	other
DNAI1	2.39E-02	2.328	dynein, axonemal, intermediate chain 1	Extracellular Space	other
DNER	6.81E-04	−1.666	delta/notch-like EGF repeat containing	Plasma Membrane	transmembrane receptor
DNPEP	2.31E-07	2.41	aspartyl aminopeptidase	Cytoplasm	peptidase
DOCK1	7.91E-04	3.778	dedicator of cytokinesis 1	Cytoplasm	other
DOCK3	1.05E-02	−2.403	dedicator of cytokinesis 3	Cytoplasm	other
DOPEY1	1.19E-02	−2.632	dopey family member 1	Cytoplasm	other
DPP4	1.62E-02	−1.895	dipeptidyl-peptidase 4	Plasma Membrane	peptidase
DPY19L2	5.52E-03	3.151	dpy-19-like 2 (C. elegans)	unknown	other
DRAP1	3.94E-03	1.506	DR1-associated protein 1 (negative cofactor 2 alpha)	Nucleus	transcription regulator
DSC1	3.79E-06	−2.639	desmocollin 1	Plasma Membrane	other
DSCC1	7.23E-05	3.157	defective in sister chromatid cohesion 1 homolog (S. cerevisiae)	Nucleus	other
DSE	1.74E-02	−1.559	dermatan sulfate epimerase	Cytoplasm	enzyme
DTYMK	3.50E-04	2.102	deoxythymidylate kinase (thymidylate kinase)	Cytoplasm	kinase
DUS3L	5.30E-03	2.071	dihydrouridine synthase 3-like (S. cerevisiae)	unknown	other
DYRK3	8.76E-03	1.76	dual-specificity tyrosine-(Y)-phosphorylation regulated kinase 3	Nucleus	kinase

E2F1	4.58E-02	−1.776	E2F transcription factor 1	Nucleus	transcription regulator
EARS2	2.93E-03	2.818	glutamyl-tRNA synthetase 2, mitochondrial (putative)	Cytoplasm	enzyme
EBF3	4.90E-02	1.595	early B-cell factor 3	Nucleus	other
EBNA1BP2	8.51E-06	1.509	EBNA1 binding protein 2	Nucleus	other
ECE2	2.60E-02	1.747	endothelin converting enzyme 2	Plasma Membrane	peptidase
ECM2 (includes EG:1842)	1.93E-03	1.681	extracellular matrix protein 2, female organ and adipocyte specific	Extracellular Space	other
ECT2	1.19E-02	1.979	epithelial cell transforming sequence 2 oncogene	Nucleus	other
EEF1E1	1.49E-04	1.879	eukaryotic translation elongation factor 1 epsilon 1	Cytoplasm	translation regulator
EFCAB2	2.24E-04	1.76	EF-hand calcium binding domain 2	unknown	other
EFHB	6.75E-03	3.231	EF-hand domain family, member B	unknown	other
EGLN3	2.79E-04	−1.897	egl nine homolog 3 (C. elegans)	Cytoplasm	enzyme
EGR4	4.29E-02	1.958	early growth response 4	Nucleus	transcription regulator
EHD4	3.29E-04	1.568	EH-domain containing 4	Plasma Membrane	enzyme
EHMT1	2.87E-04	3.026	euchromatic histone-lysine N-methyltransferase 1	Nucleus	transcription regulator
EID3	4.18E-05	1.719	EP300 interacting inhibitor of differentiation 3	Cytoplasm	other
EIF2AK4	9.12E-05	3.34	eukaryotic translation initiation factor 2 alpha kinase 4	Cytoplasm	kinase
EIF2C4	4.91E-02	2.647	eukaryotic translation initiation factor 2C, 4	Cytoplasm	translation regulator
EIF5A	4.76E-03	1.74	eukaryotic translation initiation factor 5A	Cytoplasm	translation regulator
ELK1	3.73E-02	2.272	ELK1, member of ETS oncogene family	Nucleus	transcription regulator
ELOVL7	7.06E-07	−1.622	ELOVL fatty acid elongase 7	Cytoplasm	enzyme
EML1	6.05E-05	−1.578	echinoderm microtubule associated protein like 1	Cytoplasm	other
EML4	4.45E-06	2.554	echinoderm microtubule associated protein like 4	Cytoplasm	other
EMP3	5.99E-03	1.66	epithelial membrane protein 3	Plasma Membrane	other
EMX1	2.38E-02	−2.371	empty spiracles homeobox 1	Nucleus	transcription regulator
ENO1	3.18E-06	1.55	enolase 1, (alpha)	Cytoplasm	transcription

					regulator
ENPP1	4.31E-02	−1.72	ectonucleotide pyrophosphatase/phosphodiesterase 1	Plasma Membrane	enzyme
EP400	4.21E-02	3.3	E1A binding protein p400	Nucleus	other
EPAG	3.64E-02	1.6	early lymphoid activation protein	Nucleus	other
EPCAM	2.40E-06	1.862	epithelial cell adhesion molecule	Plasma Membrane	other
EPHA4	9.01E-04	−3.793	EPH receptor A4	Plasma Membrane	kinase
EPHX4	2.25E-03	1.831	epoxide hydrolase 4	unknown	enzyme
ERCC6L	7.01E-07	4.243	excision repair cross-complementing rodent repair deficiency, complementation group 6-like	Nucleus	other
ERI3	1.61E-02	1.8	ERI1 exoribonuclease family member 3	Plasma Membrane	other
ERICH1	1.79E-03	1.752	glutamate-rich 1	unknown	other
ESCO2	1.39E-02	2.121	establishment of cohesion 1 homolog 2 (S. cerevisiae)	Nucleus	other
ESYT2	2.15E-03	3.775	extended synaptotagmin-like protein 2	unknown	other
ETV6	1.92E-02	1.788	ets variant 6	Nucleus	transcription regulator
EXO1 (includes EG:26909)	3.90E-05	2.384	exonuclease 1	Nucleus	enzyme
EXOG	2.02E-02	2.586	endo/exonuclease (5'-3'), endonuclease G-like	Cytoplasm	enzyme
F2RL3	1.60E-02	2.57	coagulation factor II (thrombin) receptor-like 3	Plasma Membrane	G-protein coupled receptor
FAAH2	4.53E-02	−1.83	fatty acid amide hydrolase 2	unknown	enzyme
FABP4	1.18E-04	1.785	fatty acid binding protein 4, adipocyte	Cytoplasm	transporter
FADS1	7.54E-06	1.539	fatty acid desaturase 1	Plasma Membrane	enzyme
FAM104B	3.10E-03	1.976	family with sequence similarity 104, member B	unknown	other
FAM111B	1.79E-02	2.823	family with sequence similarity 111, member B	unknown	other
FAM120C	7.53E-05	1.841	family with sequence similarity 120C	unknown	other
FAM126B	4.45E-05	−1.512	family with sequence similarity 126, member B	unknown	other
FAM129A	8.25E-04	1.68	family with sequence similarity 129, member A	Cytoplasm	other

FAM149A	1.28E-02	3.089	family with sequence similarity 149, member A	unknown	other
FAM158A	8.86E-03	1.777	family with sequence similarity 158, member A	Plasma Membrane	other
FAM190A	7.05E-03	−2.873	family with sequence similarity 190, member A	unknown	other
FAM198B	1.40E-02	3.236	family with sequence similarity 198, member B	Cytoplasm	other
FAM205A	1.40E-02	4.235	family with sequence similarity 205, member A	unknown	other
FAM43B	2.69E-02	−2.564	family with sequence similarity 43, member B	unknown	other
FAM45B	4.27E-02	2.067	family with sequence similarity 45, member A pseudogene	unknown	other
FAM63A	5.74E-03	−2.542	family with sequence similarity 63, member A	unknown	other
FAM71E1	2.23E-02	−2.099	family with sequence similarity 71, member E1	unknown	other
FAM82B	4.77E-02	2.604	family with sequence similarity 82, member B	Cytoplasm	other
FANCF	1.77E-08	2.868	Fanconi anemia, complementation group F	Nucleus	other
FANCI	1.21E-06	3.031	Fanconi anemia, complementation group I	Nucleus	other
FANCM	1.53E-02	2.009	Fanconi anemia, complementation group M	Nucleus	enzyme
FARSA	6.34E-06	3.804	phenylalanyl-tRNA synthetase, alpha subunit	Cytoplasm	enzyme
FARSB	1.98E-06	2.442	phenylalanyl-tRNA synthetase, beta subunit	Cytoplasm	enzyme
FAS	1.92E-05	1.57	Fas (TNF receptor superfamily, member 6)	Plasma Membrane	transmembrane receptor
FBLIM1	3.25E-03	1.706	filamin binding LIM protein 1	Plasma Membrane	other
FBXO43	3.51E-02	2.022	F-box protein 43	unknown	other
FCGRT	1.10E-03	−2.156	Fc fragment of IgG, receptor, transporter, alpha	Plasma Membrane	transmembrane receptor
FCRL5	1.67E-02	−1.609	Fc receptor-like 5	unknown	other
FCRLA	3.12E-03	1.653	Fc receptor-like A	Plasma Membrane	other
FETUB	1.06E-02	−2.797	fetuin B	Extracellular Space	other
FGD6	1.25E-04	1.563	FYVE, RhoGEF and PH domain containing 6	Cytoplasm	other
FGF9	2.45E-02	5.02	fibroblast growth factor 9 (glia-activating factor)	Extracellular Space	growth factor

FH	2.13E-03	2.023	fumarate hydratase	Cytoplasm	enzyme
FIGLA	3.19E-03	3.631	folliculogenesis specific basic helix-loop-helix	Nucleus	other
FLG2	6.31E-05	−2.036	filaggrin family member 2	unknown	other
FLG	3.83E-04	−2.111	filaggrin	Cytoplasm	other
FLI1	5.19E-03	2.313	Friend leukemia virus integration 1	Nucleus	transcription regulator
FLJ12120	2.57E-03	1.937	uncharacterized LOC388439	unknown	other
FLJ20464	3.20E-02	−1.984	uncharacterized protein FLJ20464	unknown	other
FLJ22763	2.72E-03	4.475	uncharacterized LOC401081	unknown	other
FLJ33630	7.07E-08	3.884	uncharacterized LOC644873	unknown	other
FLJ34208	2.19E-02	2.217	uncharacterized LOC401106	unknown	other
FLJ35390	3.01E-03	−2.655	uncharacterized LOC255031	unknown	other
FLJ40288	1.20E-03	2.456	uncharacterized FLJ40288	unknown	other
FLJ42627	1.88E-03	3.26	uncharacterized LOC645644	unknown	other
FMR1NB	1.54E-02	3.657	fragile X mental retardation 1 neighbor	unknown	other
FNDC3B	1.59E-03	1.857	fibronectin type III domain containing 3B	Cytoplasm	other
FOXC1	5.25E-06	−1.653	forkhead box C1	Nucleus	transcription regulator
FOXE1	6.40E-05	−1.617	forkhead box E1 (thyroid transcription factor 2)	Nucleus	transcription regulator
FOXJ2	4.15E-03	1.965	forkhead box J2	Nucleus	transcription regulator
FOXP3	4.98E-02	1.528	forkhead box P3	Nucleus	transcription regulator
FOXRED2	4.77E-02	−1.521	FAD-dependent oxidoreductase domain containing 2	Cytoplasm	other
FP588	4.57E-02	−1.812	uncharacterized LOC92973	unknown	other
FXYD6	3.86E-03	−2.16	FXYD domain containing ion transport regulator 6	Plasma Membrane	ion channel
GABRE	6.05E-03	−1.637	gamma-aminobutyric acid (GABA) A receptor, epsilon	Plasma Membrane	ion channel
GAGE1 (includes others)	3.84E-02	−2.251	G antigen 1	unknown	other
GALM	8.60E-03	1.857	galactose mutarotase (aldose 1-epimerase)	Cytoplasm	enzyme
GALNT14	1.65E-05	1.765	UDP-N-acetyl-alpha-D-galactosamine:polypeptide N-acetylgalactosaminyltransferase 14 (GalNAc-T14)	Cytoplasm	enzyme
GAN	1.51E-02	−2.064	gigaxonin	Cytoplasm	other
GART	1.30E-02	1.602	phosphoribosylglycinamide formyltransferase, phosphoribosylglycinamide	Cytoplasm	enzyme

			synthetase, phosphoribosylaminoimidazole synthetase		
GATA3	8.60E-05	−1.863	GATA binding protein 3	Nucleus	transcription regulator
GBP1	9.06E-05	1.703	guanylate binding protein 1, interferon-inducible	Cytoplasm	enzyme
GCFC2	6.04E-04	1.994	GC-rich sequence DNA-binding factor 2	Nucleus	transcription regulator
GCNT1	4.52E-04	2.052	glucosaminyl (N-acetyl) transferase 1, core 2	Cytoplasm	enzyme
GCNT2	4.48E-03	3.144	glucosaminyl (N-acetyl) transferase 2, I-branching enzyme (I blood group)	Cytoplasm	enzyme
GCOM1	2.22E-04	−1.632	GRINL1A complex locus 1	Nucleus	other
GEMIN8	3.99E-03	1.534	gem (nuclear organelle) associated protein 8	Cytoplasm	other
GFPT2	2.56E-03	−1.511	glutamine-fructose-6-phosphate transaminase 2	Cytoplasm	enzyme
GIMAP5	2.93E-02	−2.847	GTPase, IMAP family member 5	Cytoplasm	other
GINS2	3.38E-05	3.098	GINS complex subunit 2 (Psf2 homolog)	Nucleus	other
GLIPR1	1.86E-04	3.209	GLI pathogenesis-related 1	Extracellular Space	other
GLP1R	3.40E-02	1.553	glucagon-like peptide 1 receptor	Plasma Membrane	G-protein coupled receptor
GLRA3	4.77E-02	2.801	glycine receptor, alpha 3	Plasma Membrane	ion channel
GLRX	5.06E-04	−1.568	glutaredoxin (thioltransferase)	Cytoplasm	enzyme
GLT1D1	1.60E-02	−2.112	glycosyltransferase 1 domain containing 1	Extracellular Space	enzyme
GLT25D1	6.92E-05	3.251	glycosyltransferase 25 domain containing 1	Cytoplasm	other
GLYCTK	9.60E-03	−2.248	glycerate kinase	Cytoplasm	kinase
GLYR1	6.58E-03	2.252	glyoxylate reductase 1 homolog (Arabidopsis)	unknown	other
GNAS	2.49E-02	1.612	GNAS complex locus	Plasma Membrane	enzyme
GNB4	2.87E-03	2.207	guanine nucleotide binding protein (G protein), beta polypeptide 4	Plasma Membrane	enzyme
GNE	5.91E-05	−1.879	glucosamine (UDP-N-acetyl)-2-epimerase/N-acetylmannosamine kinase	Cytoplasm	kinase
GNRH1	4.61E-03	1.716	gonadotropin-releasing hormone	Extracellular	other

			1 (luteinizing-releasing hormone)	Space	
GNRHR2	5.50E-04	2.584	gonadotropin-releasing hormone (type 2) receptor 2	Plasma Membrane	G-protein coupled receptor
GPATCH4	1.66E-04	1.684	G patch domain containing 4	unknown	other
GPIHBP1	1.71E-02	−1.824	glycosylphosphatidylinositol anchored high density lipoprotein binding protein 1	Plasma Membrane	transporter
GPM6B	2.66E-02	1.717	glycoprotein M6B	Plasma Membrane	other
GPR3	4.46E-02	−3.035	G protein-coupled receptor 3	Plasma Membrane	G-protein coupled receptor
GPR4	4.16E-02	2.034	G protein-coupled receptor 4	Plasma Membrane	G-protein coupled receptor
GPR26	3.52E-02	1.8	G protein-coupled receptor 26	Plasma Membrane	G-protein coupled receptor
GPR68	2.48E-02	2.162	G protein-coupled receptor 68	Plasma Membrane	G-protein coupled receptor
GPR85	2.50E-02	3.554	G protein-coupled receptor 85	Plasma Membrane	G-protein coupled receptor
GPR143	2.92E-02	−2.245	G protein-coupled receptor 143	Plasma Membrane	G-protein coupled receptor
GPR155	7.02E-04	1.907	G protein-coupled receptor 155	Plasma Membrane	G-protein coupled receptor
GPR180	2.24E-02	1.904	G protein-coupled receptor 180	unknown	other
GRAMD1C	2.88E-03	−1.647	GRAM domain containing 1C	unknown	other
GRB10	4.99E-02	2.415	growth factor receptor-bound protein 10	Cytoplasm	other
GRK4	2.03E-02	−1.914	G protein-coupled receptor kinase 4	Plasma Membrane	kinase
GRPEL2	4.32E-05	−1.511	GrpE-like 2, mitochondrial (E. coli)	Cytoplasm	other
GSR	7.47E-05	2.473	glutathione reductase	Cytoplasm	enzyme
GSTA4	1.64E-02	−1.517	glutathione S-transferase alpha 4	Cytoplasm	enzyme
GSTT2/GSTT2B	2.81E-03	1.874	glutathione S-transferase theta 2	Cytoplasm	enzyme
GTF2H3	1.11E-03	1.594	general transcription factor IIH, polypeptide 3, 34kDa	Nucleus	transcription regulator
GTPBP2	2.91E-03	2.006	GTP binding protein 2	unknown	enzyme
GTSE1	3.74E-02	−1.532	G-2 and S-phase expressed 1	Cytoplasm	other

H2AFY2	4.73E-07	2.15	H2A histone family, member Y2	Nucleus	other
HBD	3.55E-02	−2.587	hemoglobin, delta	Cytoplasm	transporter
HCG27	3.41E-02	2.058	HLA complex group 27 (non-protein coding)	unknown	other
HDAC8	9.75E-04	1.658	histone deacetylase 8	Nucleus	transcription regulator
HEATR2	4.91E-04	2.232	HEAT repeat containing 2	unknown	other
HELLS	1.56E-02	2.361	helicase, lymphoid-specific	Nucleus	enzyme
HEMK1	6.98E-05	2.249	HemK methyltransferase family member 1	Nucleus	enzyme
HERC6	2.66E-02	1.878	hect domain and RLD 6	Cytoplasm	enzyme
HIST3H3	1.95E-02	−2.761	histone cluster 3, H3	Nucleus	other
HLA-DOB	8.20E-04	2.702	major histocompatibility complex, class II, DO beta	Plasma Membrane	transmembrane receptor
HM13	2.03E-03	3.96	histocompatibility (minor) 13	Cytoplasm	peptidase
HMHB1	5.39E-03	2.758	histocompatibility (minor) HB-1	unknown	other
HN1L	1.01E-03	2.836	hematological and neurological expressed 1-like	Cytoplasm	other
HNMT	2.19E-02	2.28	histamine N-methyltransferase	Cytoplasm	enzyme
HOXA7	1.92E-03	2.407	homeobox A7	Nucleus	transcription regulator
HOXB1	1.57E-02	1.762	homeobox B1	Nucleus	transcription regulator
HOXD4	1.10E-03	2.148	homeobox D4	Nucleus	transcription regulator
HPSE	3.47E-06	−1.984	heparanase	Plasma Membrane	enzyme
HS6ST2	2.98E-02	3.34	heparan sulfate 6-O-sulfotransferase 2	Plasma Membrane	enzyme
HSP90AB1	1.35E-04	1.666	heat shock protein 90kDa alpha (cytosolic), class B member 1	Cytoplasm	enzyme
HSPB2	4.26E-03	−2	heat shock 27kDa protein 2	Cytoplasm	other
HSPD1	2.21E-06	1.868	heat shock 60kDa protein 1 (chaperonin)	Cytoplasm	enzyme
HSPE1	2.67E-08	1.662	heat shock 10kDa protein 1 (chaperonin 10)	Cytoplasm	enzyme
HTATSF1	4.78E-03	1.706	HIV-1 Tat specific factor 1	Nucleus	transcription regulator
HTR6	2.32E-03	−1.974	5-hydroxytryptamine (serotonin) receptor 6	Plasma Membrane	G-protein coupled receptor
ICAM2	3.93E-02	1.522	intercellular adhesion molecule 2	Plasma Membrane	other
IFI6	1.78E-02	3.053	interferon, alpha-inducible protein 6	Cytoplasm	other
IFI16	2.17E-03	1.617	interferon, gamma-inducible	Nucleus	transcription

			protein 16		regulator
IFIT1	4.72E-05	3.393	interferon-induced protein with tetratricopeptide repeats 1	Cytoplasm	other
IFIT2	2.64E-06	3.214	interferon-induced protein with tetratricopeptide repeats 2	Cytoplasm	other
IFIT3	8.07E-04	1.662	interferon-induced protein with tetratricopeptide repeats 3	Cytoplasm	other
IFNAR1	4.45E-03	2.418	interferon (alpha, beta and omega) receptor 1	Plasma Membrane	transmembrane receptor
IGFBP4	1.06E-03	2.258	insulin-like growth factor binding protein 4	Extracellular Space	other
IGFBP5	8.15E-05	3.111	insulin-like growth factor binding protein 5	Extracellular Space	other
IKBKB	1.34E-02	2.078	inhibitor of kappa light polypeptide gene enhancer in B-cells, kinase beta	Cytoplasm	kinase
IL6	1.99E-04	2.481	interleukin 6 (interferon, beta 2)	Extracellular Space	cytokine
IL24	3.54E-05	2.893	interleukin 24	Extracellular Space	cytokine
IL33	4.65E-07	3.261	interleukin 33	Extracellular Space	cytokine
IL12RB1	3.25E-02	−2.132	interleukin 12 receptor, beta 1	Plasma Membrane	transmembrane receptor
IL13RA2	1.94E-02	2.195	interleukin 13 receptor, alpha 2	Plasma Membrane	transmembrane receptor
IL1R2	1.15E-04	1.523	interleukin 1 receptor, type II	Plasma Membrane	transmembrane receptor
IL1RL1	6.54E-04	2.782	interleukin 1 receptor-like 1	Plasma Membrane	transmembrane receptor
IL20RA	1.29E-02	−3.873	interleukin 20 receptor, alpha	Plasma Membrane	transmembrane receptor
ILF3	1.79E-04	2.182	interleukin enhancer binding factor 3, 90kDa	Nucleus	transcription regulator
INO80 (includes EG:296084)	9.75E-04	3.233	INO80 homolog (S. cerevisiae)	Nucleus	enzyme
INPP5J	3.65E-02	−1.564	inositol polyphosphate-5-phosphatase J	Cytoplasm	phosphatase
INSIG1	2.74E-04	−1.615	insulin induced gene 1	Cytoplasm	other
IRX3	1.30E-07	−1.673	iroquois homeobox 3	Nucleus	transcription regulator
ITGA4	9.38E-03	3.252	integrin, alpha 4 (antigen CD49D, alpha 4 subunit of VLA-4 receptor)	Plasma Membrane	other
ITGB7	4.76E-03	−1.976	integrin, beta 7	Plasma Membrane	transmembrane receptor

ITM2A	8.80E-04	2.438	integral membrane protein 2A	Plasma Membrane	other
IWS1	2.27E-04	3.047	IWS1 homolog (S. cerevisiae)	Nucleus	enzyme
KALRN	3.20E-02	2.016	kalirin, RhoGEF kinase	Cytoplasm	kinase
KAZALD1	2.47E-02	1.547	Kazal-type serine peptidase inhibitor domain 1	Extracellular Space	other
KCNAB1	3.32E-02	1.92	potassium voltage-gated channel, shaker-related subfamily, beta member 1	Plasma Membrane	ion channel
KCNG3	4.77E-02	1.556	potassium voltage-gated channel, subfamily G, member 3	Cytoplasm	ion channel
KCNK4	3.51E-02	2.562	potassium channel, subfamily K, member 4	Plasma Membrane	ion channel
KCNK7	1.28E-02	−1.567	potassium channel, subfamily K, member 7	Plasma Membrane	ion channel
KCNK9	1.97E-02	−2.189	potassium channel, subfamily K, member 9	Plasma Membrane	ion channel
KDM4D	4.25E-02	2.747	lysine (K)-specific demethylase 4D	unknown	other
KIAA1383	6.99E-03	−1.913	KIAA1383	Extracellular Space	other
KIAA1456	1.38E-02	2.071	KIAA1456	unknown	other
KIAA1755	1.07E-02	2.895	KIAA1755	unknown	other
KIF23	1.89E-04	2.678	kinesin family member 23	Cytoplasm	other
KIF18B	3.31E-05	2.203	kinesin family member 18B	Nucleus	other
KIF5A	5.81E-03	3.465	kinesin family member 5A	Cytoplasm	transporter
KITLG	8.93E-04	1.577	KIT ligand	Extracellular Space	growth factor
KLC3	3.37E-02	1.604	kinesin light chain 3	Cytoplasm	enzyme
KLHDC4	2.73E-03	1.687	kelch domain containing 4	unknown	other
KLHL6	4.01E-02	2.389	kelch-like 6 (Drosophila)	unknown	other
KLHL9	1.64E-03	4.346	kelch-like 9 (Drosophila)	Cytoplasm	other
KLHL14	2.18E-02	1.969	kelch-like 14 (Drosophila)	unknown	other
KLHL35	1.85E-02	2.029	kelch-like 35 (Drosophila)	unknown	other
KLRG2	1.23E-03	1.758	killer cell lectin-like receptor subfamily G, member 2	unknown	other
KLRK1	1.77E-02	2.214	killer cell lectin-like receptor subfamily K, member 1	Plasma Membrane	transmembrane receptor
KPNA5	1.50E-03	1.896	karyopherin alpha 5 (importin alpha 6)	Nucleus	other
KRT1	8.57E-06	−1.662	keratin 1	Cytoplasm	other
KRT2	3.15E-05	−2.076	keratin 2	Cytoplasm	other
KRT4	6.68E-03	−2.203	keratin 4	Cytoplasm	other
KRT8	2.53E-04	1.987	keratin 8	Cytoplasm	other
KRT27	9.71E-04	1.896	keratin 27	unknown	other
KRT77	1.20E-05	−2.751	keratin 77	unknown	other

KRT79	3.74E-02	−1.895	keratin 79	unknown	other
KRT81	2.76E-02	−1.622	keratin 81	Cytoplasm	other
KRTAP2-4 (includes others)	3.27E-06	3.477	keratin associated protein 2-4	unknown	other
L2HGDH	3.86E-03	1.989	L-2-hydroxyglutarate dehydrogenase	Cytoplasm	enzyme
LAMA3	3.15E-03	1.739	laminin, alpha 3	Extracellular Space	other
LAMC3	5.03E-05	2.509	laminin, gamma 3	Extracellular Space	other
LAPTM4B	9.82E-03	1.561	lysosomal protein transmembrane 4 beta	unknown	other
LARP4	1.96E-02	2.264	La ribonucleoprotein domain family, member 4	unknown	other
LARP1B	6.54E-05	3.04	La ribonucleoprotein domain family, member 1B	unknown	other
LAS1L	1.83E-04	2.992	LAS1-like (S. cerevisiae)	Nucleus	other
LCORL	4.66E-03	−1.563	ligand dependent nuclear receptor corepressor-like	Nucleus	transcription regulator
LCP2	1.44E-02	3.021	lymphocyte cytosolic protein 2 (SH2 domain containing leukocyte protein of 76kDa)	Cytoplasm	other
LENG9	1.92E-02	1.745	leukocyte receptor cluster (LRC) member 9	unknown	other
LIG1	3.27E-02	1.872	ligase I, DNA, ATP-dependent	Nucleus	enzyme
LIG3	9.12E-04	2.222	ligase III, DNA, ATP-dependent	Nucleus	enzyme
LILRA1	2.57E-02	−1.62	leukocyte immunoglobulin-like receptor, subfamily A (with TM domain), member 1	Plasma Membrane	transmembrane receptor
LILRA2	2.82E-03	2.408	leukocyte immunoglobulin-like receptor, subfamily A (with TM domain), member 2	Plasma Membrane	other
LILRB5	2.12E-02	3.032	leukocyte immunoglobulin-like receptor, subfamily B (with TM and ITIM domains), member 5	Plasma Membrane	transmembrane receptor
LILRP2	1.47E-02	1.8	leukocyte immunoglobulin-like receptor pseudogene 2	unknown	other
LIMD2	3.41E-02	−2.945	LIM domain containing 2	unknown	other
LIMS1	3.30E-02	3.12	LIM and senescent cell antigen-like domains 1	Cytoplasm	other
LINC00106	1.93E-02	2.278	long intergenic non-protein coding RNA 106	unknown	other
LINC00326	6.75E-03	1.866	long intergenic non-protein coding RNA 326	unknown	other
LINC00518	8.97E-05	2.071	long intergenic non-protein coding RNA 518	unknown	other

LINC00528	4.53E-03	−2.273	long intergenic non-protein coding RNA 528	unknown	other
LMNB1	3.66E-05	2.794	lamin B1	Nucleus	other
LMX1A	4.41E-02	−2.027	LIM homeobox transcription factor 1, alpha	Nucleus	transcription regulator
LOC150005	3.35E-02	−2.875	uncharacterized LOC150005	unknown	other
LOC151300	4.20E-03	−2.88	uncharacterized LOC151300	unknown	other
LOC153811	4.52E-03	1.709	uncharacterized LOC153811	unknown	other
LOC283070	1.16E-02	−1.705	uncharacterized LOC283070	unknown	other
LOC283392	3.69E-02	−2.995	uncharacterized LOC283392	unknown	other
LOC284475	4.54E-04	−3.082	uncharacterized LOC284475	unknown	other
LOC284561	2.67E-03	2.316	uncharacterized LOC284561	unknown	other
LOC284788	3.96E-02	−2.515	uncharacterized LOC284788	unknown	other
LOC285954	4.74E-02	1.962	uncharacterized LOC285954	unknown	other
LOC286359	2.38E-02	−1.819	uncharacterized LOC286359	unknown	other
LOC339760	4.23E-02	2.474	uncharacterized LOC339760	unknown	other
LOC340090	1.32E-02	−3.316	uncharacterized LOC340090	unknown	other
LOC340107	4.70E-02	2.824	uncharacterized LOC340107	unknown	other
LOC386758	1.27E-02	2.499	uncharacterized LOC386758	unknown	other
LOC389831	3.72E-03	1.616	uncharacterized LOC389831	unknown	other
LOC400236	3.78E-02	2.467	uncharacterized LOC400236	unknown	other
LOC401220	2.91E-02	3.089	uncharacterized LOC401220	unknown	other
LOC441528	9.71E-05	1.912	uncharacterized LOC441528	unknown	other
LOC494558	1.58E-03	3.628	uncharacterized LOC494558	unknown	other
LOC574538	4.46E-03	1.887	uncharacterized LOC574538	unknown	other
LOC643623	4.63E-02	2.749	uncharacterized LOC643623	unknown	other
LOC643650	4.83E-02	1.561	uncharacterized LOC643650	unknown	other
LOC643837	4.18E-02	−1.996	uncharacterized LOC643837	unknown	other
LOC643923	7.09E-04	−2.13	uncharacterized LOC643923	unknown	other
LOC646588	1.22E-02	1.629	uncharacterized LOC646588	unknown	other
LOC646626	1.47E-02	1.64	uncharacterized LOC646626	unknown	other
LOC646627	1.62E-03	2.393	phospholipase inhibitor	unknown	other
LOC647979	2.18E-03	1.504	uncharacterized LOC647979	unknown	other
LOC728061	1.74E-02	2.247	hCG2003663	unknown	other
LOC728175	1.41E-05	4.383	uncharacterized LOC728175	unknown	other
LOC730091	1.33E-03	1.633	uncharacterized LOC730091	unknown	other
LOC100128176	5.50E-03	−2.064	uncharacterized LOC100128176	unknown	other
LOC100128501	1.59E-03	1.741	uncharacterized LOC100128501	unknown	other
LOC100128946	8.94E-03	−2.301	uncharacterized LOC100128946	unknown	other
LOC100129858	2.27E-02	−2.337	uncharacterized LOC100129858	unknown	other
LOC100130275	1.57E-02	−1.708	uncharacterized LOC100130275	unknown	other
LOC100130428	1.52E-02	1.6	IGYY565	unknown	other
LOC100131551	4.97E-02	−3.723	uncharacterized LOC100131551	unknown	other
LOC100132987	4.77E-02	2.087	uncharacterized LOC100132987	unknown	other
LOC100271832	3.50E-02	3.057	uncharacterized LOC100271832	unknown	other
LOC100286793	3.66E-04	2.323	uncharacterized LOC100286793	unknown	other

LOC100289269	2.64E-03	−1.571	uncharacterized LOC100289269	unknown	other
LOC100505518	4.01E-02	−2.497	uncharacterized LOC100505518	unknown	other
LOC100505812	1.14E-02	1.924	uncharacterized LOC100505812	unknown	other
LOC100506207	1.55E-03	2.364	uncharacterized LOC100506207	unknown	other
LOC100506328	3.79E-03	1.891	uncharacterized LOC100506328	unknown	other
LOC100506606	6.97E-04	2.835	uncharacterized LOC100506606	unknown	other
LOC100506795	4.02E-02	3.096	uncharacterized LOC100506795	unknown	other
LOC100507462	4.44E-04	2.291	uncharacterized LOC100507462	unknown	other
LOC100507501	2.51E-02	−2.383	uncharacterized LOC100507501	unknown	other
LOC100508120	1.77E-03	2.356	uncharacterized LOC100508120	unknown	other
LOC100527964	1.09E-03	1.847	uncharacterized LOC100527964	unknown	other
LOC100505793/SRSF10	6.06E-03	1.517	serine/arginine-rich splicing factor 10	Nucleus	other
LOC389906/LOC729162	1.86E-03	1.668	zinc finger protein 839 pseudogene	unknown	other
LONRF3	4.08E-04	2.481	LON peptidase N-terminal domain and ring finger 3	unknown	other
LPXN	4.32E-02	2.215	leupaxin	Cytoplasm	other
LRRC34	2.87E-02	2.99	leucine rich repeat containing 34	unknown	other
LRRC56	3.23E-02	−2.255	leucine rich repeat containing 56	unknown	other
LRRIQ1	8.10E-03	4.162	leucine-rich repeats and IQ motif containing 1	unknown	other
LRRN3	2.52E-02	2.561	leucine rich repeat neuronal 3	Extracellular Space	other
LRWD1	2.16E-05	2.193	leucine-rich repeats and WD repeat domain containing 1	Nucleus	other
LSM2	7.91E-05	1.647	LSM2 homolog, U6 small nuclear RNA associated (S. cerevisiae)	Nucleus	other
LSM7	3.43E-05	1.809	LSM7 homolog, U6 small nuclear RNA associated (S. cerevisiae)	Nucleus	other
LY6G6E	3.66E-02	−2.609	lymphocyte antigen 6 complex, locus G6E (pseudogene)	unknown	other
LYAR	3.27E-04	1.747	Ly1 antibody reactive homolog (mouse)	Plasma Membrane	other
LYG2	7.87E-04	−3.666	lysozyme G-like 2	Extracellular Space	enzyme
MAD2L1	8.02E-09	2.575	MAD2 mitotic arrest deficient-like 1 (yeast)	Nucleus	other
MAGEA1	4.62E-02	2.343	melanoma antigen family A, 1 (directs expression of antigen MZ2-E)	Plasma Membrane	other
MAGIX	4.58E-02	2.219	MAGI family member, X-linked	unknown	other
MANSC1	1.23E-05	−1.631	MANSC domain containing 1	unknown	other
MAP1A	2.96E-03	2.735	microtubule-associated protein 1A	Cytoplasm	other
MAP1B	1.12E-03	1.866	microtubule-associated protein 1B	Cytoplasm	other

MAP3K2	9.36E-03	3.004	mitogen-activated protein kinase kinase kinase 2	Cytoplasm	kinase
MAPK1	4.93E-02	2.536	mitogen-activated protein kinase 1	Cytoplasm	kinase
MAPRE3	2.22E-02	−1.904	microtubule-associated protein, RP/EB family, member 3	Cytoplasm	enzyme
3-Mar	1.93E-03	−1.671	membrane-associated ring finger (C3HC4) 3	Cytoplasm	other
MARCKS	3.46E-03	2.417	myristoylated alanine-rich protein kinase C substrate	Plasma Membrane	other
MAS1L	9.31E-03	3.296	MAS1 oncogene-like	Plasma Membrane	G-protein coupled receptor
MATR3	2.00E-02	1.779	matrin 3	Nucleus	other
MBNL3	9.86E-05	2.325	muscleblind-like 3 (Drosophila)	Nucleus	other
MC2R	8.58E-03	2.424	melanocortin 2 receptor (adrenocorticotropic hormone)	Plasma Membrane	G-protein coupled receptor
MCAM	2.93E-05	1.531	melanoma cell adhesion molecule	Plasma Membrane	other
MCM4	4.25E-04	1.871	minichromosome maintenance complex component 4	Nucleus	enzyme
MCM10 (includes EG:307126)	1.27E-07	1.988	minichromosome maintenance complex component 10	Nucleus	other
MCMBP	3.43E-02	−2.797	minichromosome maintenance complex binding protein	Nucleus	other
MDM2	1.40E-04	3.611	Mdm2 p53 binding protein homolog (mouse)	Nucleus	transcription regulator
MDM1 (includes EG:17245)	1.08E-03	2.672	Mdm1 nuclear protein homolog (mouse)	unknown	other
MED25 (includes EG:292889)	1.51E-03	2.461	mediator complex subunit 25	Nucleus	other
MEG3	3.77E-03	1.976	maternally expressed 3 (non-protein coding)	unknown	other
MEPE	2.52E-04	−2.602	matrix extracellular phosphoglycoprotein	Extracellular Space	other
MERTK	4.36E-03	1.86	c-mer proto-oncogene tyrosine kinase	Plasma Membrane	kinase
METAP2	5.28E-04	2.275	methionyl aminopeptidase 2	Cytoplasm	peptidase
METTL7A	1.81E-03	−2.32	methyltransferase like 7A	unknown	other
MFAP3L	2.35E-05	−2.045	microfibrillar-associated protein 3-like	unknown	other
MFI2-AS1	3.01E-02	2.48	MFI2 antisense RNA 1 (non-protein coding)	unknown	other
MFSD3	2.51E-04	2.709	major facilitator superfamily domain containing 3	unknown	other

MIS18BP1	2.82E-02	−3.259	MIS18 binding protein 1	Nucleus	other
MLLT10	8.69E-04	−3.265	myeloid/lymphoid or mixed-lineage leukemia (trithorax homolog, Drosophila); translocated to, 10	Nucleus	transcription regulator
MMP8	1.31E-02	5.798	matrix metallopeptidase 8 (neutrophil collagenase)	Extracellular Space	peptidase
MMP11	2.86E-02	3.001	matrix metallopeptidase 11 (stromelysin 3)	Extracellular Space	peptidase
MMP12	2.07E-04	1.903	matrix metallopeptidase 12 (macrophage elastase)	Extracellular Space	peptidase
MNAT1	9.94E-06	1.827	menage a trois homolog 1, cyclin H assembly factor (Xenopus laevis)	Nucleus	other
MPHOSPH9	2.47E-04	2.119	M-phase phosphoprotein 9	Cytoplasm	other
MPP2	6.58E-03	1.815	membrane protein, palmitoylated 2 (MAGUK p55 subfamily member 2)	Plasma Membrane	kinase
MPZL3	6.42E-05	−1.648	myelin protein zero-like 3	unknown	other
MRC1 (includes EG:100286774)	4.92E-03	2.362	mannose receptor, C type 1	Plasma Membrane	transmembrane receptor
MRPL12	8.70E-04	1.703	mitochondrial ribosomal protein L12	Cytoplasm	other
MRPL55	2.55E-03	1.552	mitochondrial ribosomal protein L55	Cytoplasm	other
MRPL24 (includes EG:295224)	3.89E-05	2.088	mitochondrial ribosomal protein L24	Cytoplasm	other
MRPL3 (includes EG:11222)	2.11E-07	1.598	mitochondrial ribosomal protein L3	Cytoplasm	other
MRPS11	1.27E-02	1.563	mitochondrial ribosomal protein S11	Cytoplasm	other
MRTO4	1.47E-05	2.156	mRNA turnover 4 homolog (S. cerevisiae)	Cytoplasm	other
MSI2	4.01E-02	−2.163	musashi homolog 2 (Drosophila)	Cytoplasm	other
MT1E	2.42E-05	1.544	metallothionein 1E	Cytoplasm	other
MT1G	3.21E-05	1.696	metallothionein 1G	Cytoplasm	other
MT1H	9.32E-06	1.546	metallothionein 1H	Cytoplasm	other
MT1X	1.45E-06	1.856	metallothionein 1X	unknown	other
MT2A	2.99E-05	1.58	metallothionein 2A	Cytoplasm	other
MTA2	1.01E-03	−2.98	metastasis associated 1 family, member 2	Nucleus	transcription regulator
MTAP	1.30E-04	2.159	methylthioadenosine phosphorylase	Nucleus	enzyme
MTFR1	3.82E-02	3.443	mitochondrial fission regulator 1	Cytoplasm	other
MTG1	1.89E-04	2.74	mitochondrial GTPase 1 homolog	Cytoplasm	other

			(S. cerevisiae)		
MUC15	1.92E-05	−2.541	mucin 15, cell surface associated	Extracellular Space	other
MUC16	1.13E-04	−3.21	mucin 16, cell surface associated	Extracellular Space	other
MYO3A	1.62E-02	2.967	myosin IIIA	Cytoplasm	kinase
MYOM2	2.73E-02	2.707	myomesin (M-protein) 2, 165kDa	Cytoplasm	other
MYOZ2	4.27E-02	1.866	myozenin 2	Cytoplasm	other
NAA15	1.92E-04	1.765	N(alpha)-acetyltransferase 15, NatA auxiliary subunit	Nucleus	transcription regulator
NAA38	2.71E-03	2.555	N(alpha)-acetyltransferase 38, NatC auxiliary subunit	Nucleus	other
NADK	2.93E-03	−1.944	NAD kinase	Cytoplasm	kinase
NASP	2.55E-04	1.909	nuclear autoantigenic sperm protein (histone-binding)	Nucleus	other
NAV2	7.12E-03	2.329	neuron navigator 2	Nucleus	other
NBAS	7.22E-03	2.515	neuroblastoma amplified sequence	unknown	other
NBPF3	4.10E-02	−2.156	neuroblastoma breakpoint family, member 3	unknown	other
NCAPG	6.66E-04	1.669	non-SMC condensin I complex, subunit G	Nucleus	other
NCLN	4.43E-03	2.361	nicalin	Cytoplasm	peptidase
NCOR2	2.33E-02	2.81	nuclear receptor corepressor 2	Nucleus	transcription regulator
NDC80	7.25E-06	2.414	NDC80 kinetochore complex component homolog (S. cerevisiae)	Nucleus	other
NDOR1	2.47E-02	−1.508	NADPH dependent diflavin oxidoreductase 1	Cytoplasm	enzyme
NDUFAF2	1.36E-05	1.517	NADH dehydrogenase (ubiquinone) 1 alpha subcomplex, assembly factor 2	Cytoplasm	other
NDUFAF4	4.15E-04	1.911	NADH dehydrogenase (ubiquinone) 1 alpha subcomplex, assembly factor 4	Cytoplasm	other
NEB	6.56E-04	−2.007	nebulin	Cytoplasm	other
NECAB3	1.39E-05	2.252	N-terminal EF-hand calcium binding protein 3	Cytoplasm	other
NEDD4L	4.70E-04	1.511	neural precursor cell expressed, developmentally down-regulated 4-like	Cytoplasm	enzyme
NEK2	9.21E-06	3.682	NIMA (never in mitosis gene a)-related kinase 2	Cytoplasm	kinase
NEK9	3.68E-03	−2.067	NIMA (never in mitosis gene a)-related kinase 9	Nucleus	kinase

NETO1	3.40E-05	2.899	neuropilin (NRP) and tolloid (TLL)-like 1	Extracellular Space	other
NF2	1.35E-03	1.626	neurofibromin 2 (merlin)	Plasma Membrane	other
NFASC	7.60E-03	1.769	neurofascin	Plasma Membrane	other
NFAT5	1.74E-03	−1.548	nuclear factor of activated T-cells 5, tonicity-responsive	Nucleus	transcription regulator
NFE2L2	2.56E-03	1.785	nuclear factor (erythroid-derived 2)-like 2	Nucleus	transcription regulator
NFX1	2.94E-03	1.959	nuclear transcription factor, X-box binding 1	Nucleus	transcription regulator
NKX2-1	4.98E-02	−2.156	NK2 homeobox 1	Nucleus	transcription regulator
NLE1	2.19E-02	−1.745	notchless homolog 1 (Drosophila)	Nucleus	enzyme
NME1 (includes EG:18102)	4.82E-06	1.958	non-metastatic cells 1, protein (NM23A) expressed in	Nucleus	kinase
NOL6	3.32E-02	2.805	nucleolar protein family 6 (RNA-associated)	Nucleus	other
NOM1	1.22E-03	2.574	nucleolar protein with MIF4G domain 1	Nucleus	other
NOP16 (includes EG:28126)	2.30E-02	1.916	NOP16 nucleolar protein homolog (yeast)	Nucleus	other
NPHP4	3.69E-02	1.666	nephronophthisis 4	Cytoplasm	other
NPIP (includes others)	4.23E-04	1.619	nuclear pore complex interacting protein	Nucleus	other
NPL	1.06E-04	2.5	N-acetylneuraminate pyruvate lyase (dihydrodipicolinate synthase)	unknown	enzyme
NPM3	2.26E-05	1.991	nucleophosmin/nucleoplasmin 3	Nucleus	other
NPTX2	2.54E-02	2.831	neuronal pentraxin II	Extracellular Space	other
NQO1	7.86E-04	1.931	NAD(P)H dehydrogenase, quinone 1	Cytoplasm	enzyme
NR1H3	2.23E-02	1.781	nuclear receptor subfamily 1, group H, member 3	Nucleus	ligand-dependent nuclear receptor
NR1H4	4.35E-02	3.526	nuclear receptor subfamily 1, group H, member 4	Nucleus	ligand-dependent nuclear receptor
NR2C2AP	1.20E-02	1.786	nuclear receptor 2C2-associated protein	Nucleus	other
NRG1 (includes EG:112400)	6.32E-06	1.989	neuregulin 1	Extracellular Space	growth factor

NRGN	1.59E-03	1.715	neurogranin (protein kinase C substrate, RC3)	Cytoplasm	other
NRK	5.89E-03	2.416	Nik related kinase	unknown	kinase
NSMAF	1.44E-03	1.508	neutral sphingomyelinase (N-SMase) activation associated factor	Cytoplasm	other
NT5C2	5.48E-04	3.467	5'-nucleotidase, cytosolic II	Cytoplasm	phosphatase
NTNG1	1.82E-02	1.996	netrin G1	Extracellular Space	other
NUB1	4.46E-02	−2.877	negative regulator of ubiquitin-like proteins 1	Nucleus	other
NUCB2	3.05E-04	−1.861	nucleobindin 2	Nucleus	other
NUDCD2	9.42E-03	2.125	NudC domain containing 2	unknown	other
NUP43	9.36E-04	1.908	nucleoporin 43kDa	Nucleus	transporter
NUP54	1.95E-02	2.389	nucleoporin 54kDa	Nucleus	transporter
NUP62	1.32E-03	1.89	nucleoporin 62kDa	Nucleus	transporter
NUP93	8.61E-05	2.695	nucleoporin 93kDa	Nucleus	other
OAZ3	2.12E-02	2.16	ornithine decarboxylase antizyme 3	Cytoplasm	other
OGFOD1	4.43E-02	1.918	2-oxoglutarate and iron-dependent oxygenase domain containing 1	unknown	other
OK/SW-CL.36	5.33E-03	1.86	OK/SW-CL.36	unknown	other
OLA1	7.97E-04	2.003	Obg-like ATPase 1	Cytoplasm	other
OPA3	2.72E-04	1.948	optic atrophy 3 (autosomal recessive, with chorea and spastic paraplegia)	Cytoplasm	other
OPN1SW	2.27E-02	−1.588	opsin 1 (cone pigments), short-wave-sensitive	Plasma Membrane	G-protein coupled receptor
OR1F2P	2.67E-02	−3.898	olfactory receptor, family 1, subfamily F, member 2	unknown	other
OR2B3	3.62E-02	2.618	olfactory receptor, family 2, subfamily B, member 3	Plasma Membrane	other
OR2H1	3.06E-02	−1.61	olfactory receptor, family 2, subfamily H, member 1	Plasma Membrane	G-protein coupled receptor
OR7A10	2.19E-02	2.494	olfactory receptor, family 7, subfamily A, member 10	Plasma Membrane	G-protein coupled receptor
OR8G2	2.75E-02	−3.88	olfactory receptor, family 8, subfamily G, member 2	Plasma Membrane	G-protein coupled receptor
OSBPL8	9.81E-04	1.68	oxysterol binding protein-like 8	Cytoplasm	other
OSBPL10	4.46E-03	2.609	oxysterol binding protein-like 10	unknown	other
OSTCP1	1.08E-04	1.874	oligosaccharyltransferase	unknown	other

			complex subunit pseudogene 1		
OTOS	4.74E-02	−1.699	otospiralin	Extracellular Space	other
OTUD1	1.75E-05	−1.565	OTU domain containing 1	unknown	other
P2RY2	4.72E-03	3.106	purinergic receptor P2Y, G-protein coupled, 2	Plasma Membrane	G-protein coupled receptor
P2RY8	2.79E-03	2.276	purinergic receptor P2Y, G-protein coupled, 8	Plasma Membrane	G-protein coupled receptor
P2RY14	1.47E-02	1.549	purinergic receptor P2Y, G-protein coupled, 14	Plasma Membrane	G-protein coupled receptor
PA2G4	2.39E-03	1.836	proliferation-associated 2G4, 38kDa	Nucleus	transcription regulator
PABPC1	1.43E-03	−2.64	poly(A) binding protein, cytoplasmic 1	Cytoplasm	translation regulator
PABPC4L	2.73E-02	4.163	poly(A) binding protein, cytoplasmic 4-like	unknown	other
PAICS	5.41E-06	1.659	phosphoribosylaminoimidazole carboxylase, phosphoribosylaminoimidazole succinocarboxamide synthetase	Cytoplasm	enzyme
PANK2	9.29E-03	2.174	pantothenate kinase 2	Cytoplasm	kinase
PANX3	4.78E-02	3.334	pannexin 3	Plasma Membrane	other
PARVA	6.93E-05	3.146	parvin, alpha	Cytoplasm	other
PBK	2.40E-06	2.405	PDZ binding kinase	Cytoplasm	kinase
PBX1	3.62E-02	−2.08	pre-B-cell leukemia homeobox 1	Nucleus	transcription regulator
PCDH8	1.16E-02	1.627	protocadherin 8	Plasma Membrane	other
PCDHB7	8.26E-03	2.548	protocadherin beta 7	Plasma Membrane	other
PCOLCE2 (includes EG:26577)	1.00E-02	1.508	procollagen C-endopeptidase enhancer 2	Extracellular Space	other
PCSK6	6.51E-04	−1.646	proprotein convertase subtilisin/kexin type 6	Extracellular Space	peptidase
PDAP1	1.13E-02	1.555	PDGFA associated protein 1	Cytoplasm	other
PDCD2	1.03E-02	1.517	programmed cell death 2	Nucleus	other
PDE1A	2.69E-02	3.061	phosphodiesterase 1A, calmodulin-dependent	Cytoplasm	enzyme
PDE2A	2.40E-02	2.268	phosphodiesterase 2A, cGMP-stimulated	Cytoplasm	enzyme
PDE3A	2.79E-03	1.618	phosphodiesterase 3A, cGMP-	Cytoplasm	enzyme

			inhibited		
PDK2	4.66E-03	−2.139	pyruvate dehydrogenase kinase, isozyme 2	Cytoplasm	kinase
PDSS1	3.49E-06	1.86	prenyl (decaprenyl) diphosphate synthase, subunit 1	Cytoplasm	enzyme
PEG10	8.17E-06	3.604	paternally expressed 10	Nucleus	other
PELP1	6.26E-04	3.317	proline, glutamate and leucine rich protein 1	Nucleus	other
PER2	6.09E-04	−1.687	period homolog 2 (Drosophila)	Nucleus	other
PERP	1.02E-04	−1.529	PERP, TP53 apoptosis effector	Plasma Membrane	other
PEX5L	3.81E-07	3.107	peroxisomal biogenesis factor 5-like	Cytoplasm	ion channel
PFAS	7.56E-08	2.475	phosphoribosylformylglycinamidine synthase	Cytoplasm	enzyme
PHC2	4.07E-02	−2.286	polyhomeotic homolog 2 (Drosophila)	Nucleus	other
PHF1	8.86E-04	2.585	PHD finger protein 1	Nucleus	transcription regulator
PHF7	7.66E-05	4.185	PHD finger protein 7	Nucleus	other
PHF19	3.47E-04	2.119	PHD finger protein 19	unknown	other
PHKA1	8.22E-04	2.485	phosphorylase kinase, alpha 1 (muscle)	Cytoplasm	kinase
PHKB	1.63E-02	4.288	phosphorylase kinase, beta	Cytoplasm	kinase
PI4K2B	1.25E-02	3.789	phosphatidylinositol 4-kinase type 2 beta	Cytoplasm	kinase
PIGN	2.78E-04	−1.696	phosphatidylinositol glycan anchor biosynthesis, class N	Cytoplasm	enzyme
PIGY	8.99E-05	1.945	phosphatidylinositol glycan anchor biosynthesis, class Y	Plasma Membrane	other
PIH2	1.39E-05	5.931	pregnancy-induced hypertension syndrome-related protein	unknown	other
PIWIL1	3.33E-04	2.686	piwi-like 1 (Drosophila)	Cytoplasm	other
PLA2G4A	1.23E-06	2.226	phospholipase A2, group IVA (cytosolic, calcium-dependent)	Cytoplasm	enzyme
PLA2R1	6.64E-03	2.608	phospholipase A2 receptor 1, 180kDa	Plasma Membrane	transmembrane receptor
PLCE1	3.36E-02	2.121	phospholipase C, epsilon 1	Cytoplasm	enzyme
PLEK	2.19E-03	1.657	pleckstrin	Cytoplasm	other
PLEKHA8P1	1.93E-02	3.134	pleckstrin homology domain containing, family A member 8 pseudogene 1	unknown	other
PLEKHB1	1.61E-02	1.731	pleckstrin homology domain containing, family B (evectins) member 1	Cytoplasm	other
PLEKHH3	1.57E-02	−3.019	pleckstrin homology domain	unknown	other

			containing, family H (with MyTH4 domain) member 3		
PLK1	7.04E-05	2.526	polo-like kinase 1	Nucleus	kinase
PLXDC2	9.03E-05	−2.314	plexin domain containing 2	Extracellular Space	other
PMS2L2	4.29E-02	1.505	postmeiotic segregation increased 2-like 2 pseudogene	unknown	other
PNO1	2.35E-04	1.505	partner of NOB1 homolog (S. cerevisiae)	Nucleus	other
PNPLA4	3.03E-02	2.124	patatin-like phospholipase domain containing 4	Cytoplasm	enzyme
POC1A	4.00E-05	1.797	POC1 centriolar protein homolog A (Chlamydomonas)	Cytoplasm	peptidase
POF1B	3.44E-04	−2.358	premature ovarian failure, 1B	Plasma Membrane	other
POLE	7.01E-03	1.728	polymerase (DNA directed), epsilon	Nucleus	enzyme
POLH	2.13E-03	2.371	polymerase (DNA directed), eta	Nucleus	enzyme
POLR1A	1.28E-04	1.534	polymerase (RNA) I polypeptide A, 194kDa	Nucleus	enzyme
POLR2L	4.45E-05	2.58	polymerase (RNA) II (DNA directed) polypeptide L, 7.6kDa	Nucleus	enzyme
POU2AF1	5.45E-04	2.394	POU class 2 associating factor 1	Nucleus	transcription regulator
POU3F1	2.80E-02	1.903	POU class 3 homeobox 1	Nucleus	transcription regulator
PPIH	4.24E-04	1.59	peptidylprolyl isomerase H (cyclophilin H)	Nucleus	enzyme
PPIL2	1.35E-02	1.582	peptidylprolyl isomerase (cyclophilin)-like 2	Nucleus	enzyme
PPIP5K1	2.11E-03	1.636	diphosphoinositol pentakisphosphate kinase 1	Nucleus	phosphatase
PPM1G	9.11E-04	2.095	protein phosphatase, Mg2+/Mn2+ dependent, 1G	Nucleus	phosphatase
PPP1R37	2.53E-02	1.644	protein phosphatase 1, regulatory subunit 37	unknown	other
PRC1 (includes EG:233406)	5.33E-08	1.519	protein regulator of cytokinesis 1	Nucleus	other
PRIM2	9.14E-03	1.673	primase, DNA, polypeptide 2 (58kDa)	Nucleus	enzyme
PRKG1	2.09E-03	−2.016	protein kinase, cGMP-dependent, type I	Cytoplasm	kinase
PRMT2	1.94E-02	2.842	protein arginine methyltransferase 2	Nucleus	enzyme
PROS1	1.49E-03	−1.686	protein S (alpha)	Extracellular Space	other

PROSC	3.31E-02	2.024	proline synthetase co-transcribed homolog (bacterial)	Cytoplasm	enzyme
PROX1	1.54E-03	2.561	prospero homeobox 1	Nucleus	transcription regulator
PRPF19	8.43E-06	3.052	PRP19/PSO4 pre-mRNA processing factor 19 homolog (S. cerevisiae)	Nucleus	enzyme
PRPF31	1.38E-04	3.227	PRP31 pre-mRNA processing factor 31 homolog (S. cerevisiae)	Nucleus	other
PRPF40A	5.04E-04	1.545	PRP40 pre-mRNA processing factor 40 homolog A (S. cerevisiae)	Nucleus	other
PRR5	6.63E-05	3.211	proline rich 5 (renal)	unknown	other
PRSS12	3.58E-03	1.885	protease, serine, 12 (neurotrypsin, motopsin)	Extracellular Space	peptidase
PSAT1	7.85E-03	1.654	phosphoserine aminotransferase 1	Cytoplasm	enzyme
PSG1	1.98E-02	2.251	pregnancy specific beta-1-glycoprotein 1	Extracellular Space	other
PSMB10	2.44E-05	1.554	proteasome (prosome, macropain) subunit, beta type, 10	Cytoplasm	peptidase
PSMD3	3.22E-02	1.663	proteasome (prosome, macropain) 26S subunit, non-ATPase, 3	Cytoplasm	other
PSMD11	1.77E-02	2.746	proteasome (prosome, macropain) 26S subunit, non-ATPase, 11	Cytoplasm	other
PSME3	3.21E-02	1.553	proteasome (prosome, macropain) activator subunit 3 (PA28 gamma; Ki)	Cytoplasm	peptidase
PSMG4	1.84E-04	1.759	proteasome (prosome, macropain) assembly chaperone 4	unknown	transcription regulator
PSPC1	2.41E-02	1.602	paraspeckle component 1	Nucleus	other
PSPH	4.71E-02	1.779	phosphoserine phosphatase	Cytoplasm	phosphatase
PTER	1.21E-03	1.64	phosphotriesterase related	unknown	enzyme
PTGDR	3.16E-02	2.188	prostaglandin D2 receptor (DP)	Plasma Membrane	G-protein coupled receptor
PTGIS	1.38E-02	1.891	prostaglandin I2 (prostacyclin) synthase	Cytoplasm	enzyme
PTK7	5.76E-04	4.11	PTK7 protein tyrosine kinase 7	Plasma Membrane	kinase
PTK2B (includes EG:19229)	2.41E-02	1.654	PTK2B protein tyrosine kinase 2 beta	Cytoplasm	kinase
PTPN18	2.86E-03	1.522	protein tyrosine phosphatase, non-receptor type 18 (brain-derived)	Nucleus	phosphatase
PTPRM	8.28E-03	2.628	protein tyrosine phosphatase, receptor type, M	Plasma Membrane	phosphatase
PVR	4.12E-02	2.279	poliovirus receptor	Plasma	other

				Membrane	
PWWP2B	1.15E-02	−1.869	PWWP domain containing 2B	unknown	other
PYHIN1	4.99E-02	2.936	pyrin and HIN domain family, member 1	Nucleus	other
QRICH2	4.73E-02	1.903	glutamine rich 2	unknown	other
QSOX2	5.73E-03	−2.425	quiescin Q6 sulfhydryl oxidase 2	unknown	enzyme
RAB3B	1.21E-02	1.912	RAB3B, member RAS oncogene family	Cytoplasm	enzyme
RAB40AL	2.81E-02	2.396	RAB40A, member RAS oncogene family-like	Plasma Membrane	other
RABL3	8.68E-05	3.363	RAB, member of RAS oncogene family-like 3	unknown	enzyme
RAC2	1.47E-03	2.318	ras-related C3 botulinum toxin substrate 2 (rho family, small GTP binding protein Rac2)	Cytoplasm	enzyme
RAC3	2.35E-02	2.385	ras-related C3 botulinum toxin substrate 3 (rho family, small GTP binding protein Rac3)	Cytoplasm	enzyme
RAD51AP1	1.09E-07	1.693	RAD51 associated protein 1	Nucleus	other
RARA	3.42E-03	−1.851	retinoic acid receptor, alpha	Nucleus	ligand-dependent nuclear receptor
RASGRP3	9.11E-03	1.52	RAS guanyl releasing protein 3 (calcium and DAG-regulated)	Cytoplasm	other
RASSF4	2.47E-02	2.661	Ras association (RalGDS/AF-6) domain family member 4	unknown	other
RASSF5	1.15E-02	−1.734	Ras association (RalGDS/AF-6) domain family member 5	Plasma Membrane	other
RBAK	4.18E-03	3.786	RB-associated KRAB zinc finger	Nucleus	transcription regulator
RBM15	2.30E-03	1.617	RNA binding motif protein 15	Nucleus	other
RBM22	7.87E-03	1.503	RNA binding motif protein 22	Nucleus	other
RBP3	3.18E-02	−2.417	retinol binding protein 3, interstitial	Extracellular Space	transporter
REEP6	4.35E-02	1.872	receptor accessory protein 6	Plasma Membrane	other
REPIN1	2.15E-03	1.547	replication initiator 1	Nucleus	other
RHAG	8.52E-03	2.682	Rh-associated glycoprotein	Plasma Membrane	peptidase
RHBG	4.42E-06	−2.334	Rh family, B glycoprotein (gene/pseudogene)	Plasma Membrane	transporter
RHEB	3.36E-02	2.263	Ras homolog enriched in brain	Cytoplasm	other
RHEBL1	3.33E-02	1.511	Ras homolog enriched in brain like 1	Cytoplasm	enzyme
RHOC	3.00E-03	1.772	ras homolog gene family, member	Plasma	enzyme

			C	Membrane	
RIPPLY2	4.81E-02	1.529	ripply2 homolog (zebrafish)	Nucleus	other
RNASEH2B	4.04E-03	1.848	ribonuclease H2, subunit B	Nucleus	other
RNF41	1.40E-03	2.458	ring finger protein 41	Cytoplasm	enzyme
RNF167	4.05E-03	1.912	ring finger protein 167	Cytoplasm	enzyme
RNF181	4.63E-03	1.779	ring finger protein 181	unknown	other
RNPS1	3.34E-05	2.816	RNA binding protein S1, serine-rich domain	Nucleus	other
ROR2	4.94E-03	2.572	receptor tyrosine kinase-like orphan receptor 2	Plasma Membrane	kinase
RPAP3	2.44E-02	1.639	RNA polymerase II associated protein 3	unknown	enzyme
RPL22L1	2.56E-06	1.814	ribosomal protein L22-like 1	unknown	other
RPL37A	1.81E-02	1.684	ribosomal protein L37a	Cytoplasm	other
RPP30	3.97E-04	3.004	ribonuclease P/MRP 30kDa subunit	Nucleus	enzyme
RPS2P45	1.09E-02	3.077	ribosomal protein S2 pseudogene 45	unknown	other
RPS6KA6	4.38E-02	1.543	ribosomal protein S6 kinase, 90kDa, polypeptide 6	Cytoplasm	kinase
RRH	8.46E-03	1.508	retinal pigment epithelium-derived rhodopsin homolog	Plasma Membrane	G-protein coupled receptor
RRM2	2.19E-05	1.647	ribonucleotide reductase M2	Nucleus	enzyme
RRN3	4.45E-04	1.614	RRN3 RNA polymerase I transcription factor homolog (S. cerevisiae)	Nucleus	transcription regulator
RRP15 (includes EG:327053)	8.64E-05	1.541	ribosomal RNA processing 15 homolog (S. cerevisiae)	Nucleus	other
RRS1	1.93E-05	3.492	RRS1 ribosome biogenesis regulator homolog (S. cerevisiae)	Nucleus	other
RSBN1	4.09E-02	1.555	round spermatid basic protein 1	Nucleus	other
RSG1	2.25E-04	2.59	REM2 and RAB-like small GTPase 1	Cytoplasm	other
RSPH4A	4.03E-02	3.177	radial spoke head 4 homolog A (Chlamydomonas)	Nucleus	other
RTKN	2.07E-03	2.224	rhotekin	Cytoplasm	other
RUFY3	4.86E-03	1.696	RUN and FYVE domain containing 3	Plasma Membrane	other
RUSC1-AS1	3.41E-03	−1.698	RUSC1 antisense RNA 1 (non-protein coding)	unknown	other
RUVBL1	3.97E-04	1.723	RuvB-like 1 (E. coli)	Nucleus	transcription regulator
S100A11	2.79E-06	3.248	S100 calcium binding protein A11	Cytoplasm	other
S1PR5	8.85E-03	−1.73	sphingosine-1-phosphate receptor	Plasma	G-protein

			5	Membrane	coupled receptor
SALL3	9.76E-04	3.44	sal-like 3 (Drosophila)	Nucleus	other
SAMD4A	2.71E-02	3.054	sterile alpha motif domain containing 4A	Cytoplasm	translation regulator
SAMD9L	8.51E-03	2.684	sterile alpha motif domain containing 9-like	unknown	other
SAMHD1	9.89E-03	3.149	SAM domain and HD domain 1	Nucleus	enzyme
SAP30L	1.78E-02	3.517	SAP30-like	unknown	other
SAV1	1.95E-04	1.561	salvador homolog 1 (Drosophila)	Cytoplasm	other
SCCPDH	4.14E-04	1.807	saccharopine dehydrogenase (putative)	Cytoplasm	other
SCGB2A1	4.53E-02	2.256	secretoglobin, family 2A, member 1	unknown	other
SCLT1	3.26E-03	2.764	sodium channel and clathrin linker 1	Plasma Membrane	transporter
SCNN1A	3.64E-02	−1.543	sodium channel, nonvoltage-gated 1 alpha	Plasma Membrane	ion channel
SCPEP1	3.17E-05	−1.871	serine carboxypeptidase 1	Cytoplasm	peptidase
SDK1	1.61E-02	−2.602	sidekick cell adhesion molecule 1	Plasma Membrane	other
SEC23IP	1.07E-02	1.613	SEC23 interacting protein	Cytoplasm	other
SECTM1	5.65E-03	1.763	secreted and transmembrane 1	Extracellular Space	cytokine
SENP3	1.29E-04	2.002	SUMO1/sentrin/SMT3 specific peptidase 3	Nucleus	peptidase
SENP6	2.06E-03	1.767	SUMO1/sentrin specific peptidase 6	Cytoplasm	peptidase
6-Sep	1.49E-02	2.184	septin 6	Cytoplasm	other
8-Sep	3.02E-06	−2.107	septin 8	Extracellular Space	other
9-Sep	4.52E-06	3.109	septin 9	Cytoplasm	enzyme
SERPINB12	1.08E-02	3.034	serpin peptidase inhibitor, clade B (ovalbumin), member 12	Cytoplasm	other
SET	4.01E-04	2.072	SET nuclear oncogene	Nucleus	phosphatase
SFTA1P	4.83E-04	1.795	surfactant associated 1, pseudogene	unknown	other
SFXN4	8.89E-05	1.975	sideroflexin 4	Cytoplasm	transporter
SGTA	4.26E-02	−1.794	small glutamine-rich tetratricopeptide repeat (TPR)-containing, alpha	Cytoplasm	other
SH3TC1	2.67E-02	2.191	SH3 domain and tetratricopeptide repeats 1	unknown	other
SHANK2	2.71E-03	1.74	SH3 and multiple ankyrin repeat domains 2	Plasma Membrane	other
SHC4	1.51E-03	1.825	SHC (Src homology 2 domain	Cytoplasm	other

			containing) family, member 4		
SHF	8.16E-04	−1.576	Src homology 2 domain containing F	unknown	other
SHKBP1	3.37E-02	2.301	SH3KBP1 binding protein 1	unknown	other
SIDT1	4.68E-02	−2.187	SID1 transmembrane family, member 1	unknown	other
SIGLEC11	2.30E-02	3.336	sialic acid binding Ig-like lectin 11	Plasma Membrane	other
SIRT4	3.31E-02	1.658	sirtuin 4	Cytoplasm	enzyme
SKIV2L2	3.54E-02	1.662	superkiller viralicidic activity 2-like 2 (S. cerevisiae)	Nucleus	other
SLA	1.09E-03	2.095	Src-like-adaptor	Plasma Membrane	other
SLAMF9	4.83E-02	2.695	SLAM family member 9	Extracellular Space	other
SLC13A2	7.42E-03	2.471	solute carrier family 13 (sodium-dependent dicarboxylate transporter), member 2	Plasma Membrane	transporter
SLC16A10	2.77E-02	2.605	solute carrier family 16, member 10 (aromatic amino acid transporter)	Plasma Membrane	transporter
SLC22A3	1.76E-04	−1.521	solute carrier family 22 (extraneuronal monoamine transporter), member 3	Plasma Membrane	transporter
SLC22A8	4.28E-02	2.88	solute carrier family 22 (organic anion transporter), member 8	Plasma Membrane	transporter
SLC23A3	7.47E-04	3.023	solute carrier family 23 (nucleobase transporters), member 3	unknown	transporter
SLC25A15	4.42E-04	1.765	solute carrier family 25 (mitochondrial carrier; ornithine transporter) member 15	Cytoplasm	transporter
SLC25A37	9.59E-05	1.864	solute carrier family 25, member 37	Cytoplasm	transporter
SLC28A1	1.86E-02	1.997	solute carrier family 28 (sodium-coupled nucleoside transporter), member 1	Plasma Membrane	transporter
SLC2A8	1.36E-02	1.985	solute carrier family 2 (facilitated glucose transporter), member 8	Plasma Membrane	transporter
SLC31A1	1.06E-04	−2.021	solute carrier family 31 (copper transporters), member 1	Plasma Membrane	transporter
SLC38A9	1.23E-03	3.097	solute carrier family 38, member 9	unknown	other
SLC39A2	4.43E-03	−1.508	solute carrier family 39 (zinc transporter), member 2	Plasma Membrane	transporter
SLC43A3	9.05E-05	1.641	solute carrier family 43, member	Extracellular	other

			3	Space	
SLC44A1	2.20E-05	−1.506	solute carrier family 44, member 1	Plasma Membrane	transporter
SLC44A5	8.66E-04	−1.671	solute carrier family 44, member 5	Plasma Membrane	transporter
SLC6A2	1.63E-02	−1.617	solute carrier family 6 (neurotransmitter transporter, noradrenalin), member 2	Plasma Membrane	transporter
SLC6A6	1.30E-03	−2.966	solute carrier family 6 (neurotransmitter transporter, taurine), member 6	Plasma Membrane	transporter
SLC9A1	2.47E-02	−2.631	solute carrier family 9 (sodium/hydrogen exchanger), member 1	Plasma Membrane	ion channel
SLC9A9	5.91E-03	−1.85	solute carrier family 9 (sodium/hydrogen exchanger), member 9	Cytoplasm	transporter
SLC9B1	3.59E-02	1.58	solute carrier family 9, subfamily B (cation proton antiporter 2), member 1	Plasma Membrane	other
SLFN5	7.17E-04	2.411	schlafen family member 5	Nucleus	enzyme
SMAD9	5.40E-03	1.596	SMAD family member 9	Nucleus	transcription regulator
SMC5	7.59E-04	1.508	structural maintenance of chromosomes 5	Nucleus	other
SMG6	1.36E-02	−2.058	smg-6 homolog, nonsense mediated mRNA decay factor (C. elegans)	Nucleus	enzyme
SMOX	9.65E-03	2.116	spermine oxidase	Cytoplasm	enzyme
SNAP23	1.27E-02	1.571	synaptosomal-associated protein, 23kDa	Plasma Membrane	transporter
SNHG8	1.52E-05	1.703	small nucleolar RNA host gene 8 (non-protein coding)	unknown	other
SNHG15	2.27E-05	1.847	small nucleolar RNA host gene 15 (non-protein coding)	unknown	other
SNRPD1	2.00E-05	1.681	small nuclear ribonucleoprotein D1 polypeptide 16kDa	Nucleus	other
SNRPF	1.02E-06	1.757	small nuclear ribonucleoprotein polypeptide F	Nucleus	other
SNTB2	6.24E-03	3.264	syntrophin, beta 2 (dystrophin-associated protein A1, 59kDa, basic component 2)	Plasma Membrane	other
SNX31	4.05E-02	−2.553	sorting nexin 31	unknown	other
SORBS2	2.70E-02	2.4	sorbin and SH3 domain containing 2	Plasma Membrane	other
SORCS1	8.74E-04	1.561	sortilin-related VPS10 domain	Plasma	transporter

			containing receptor 1	Membrane	
SORCS2	3.59E-02	−1.791	sortilin-related VPS10 domain containing receptor 2	Plasma Membrane	transporter
SOX30	4.01E-03	3.807	SRY (sex determining region Y)-box 30	Nucleus	transcription regulator
SP100	3.40E-04	2.274	SP100 nuclear antigen	Nucleus	transcription regulator
SPAG16	9.56E-03	−3.254	sperm associated antigen 16	Cytoplasm	other
SPAG11B	2.11E-02	−2.826	sperm associated antigen 11B	Extracellular Space	other
SPEF2	7.39E-03	−1.784	sperm flagellar 2	unknown	other
SPIN3	3.40E-05	2.569	spindlin family, member 3	unknown	other
SPIN4	7.45E-05	−2.021	spindlin family, member 4	unknown	other
SPN	1.96E-02	2.006	sialophorin	Plasma Membrane	transmembrane receptor
SPTLC3	1.05E-04	−2.935	serine palmitoyltransferase, long chain base subunit 3	Cytoplasm	enzyme
SQLE	7.52E-04	−1.748	squalene epoxidase	Cytoplasm	enzyme
SRFBP1	2.40E-02	1.54	serum response factor binding protein 1	Nucleus	other
SRM	1.37E-05	1.579	spermidine synthase	Cytoplasm	enzyme
SRPR	3.21E-02	2.781	signal recognition particle receptor (docking protein)	Cytoplasm	other
SRSF7	6.37E-06	1.691	serine/arginine-rich splicing factor 7	Nucleus	other
SSB	1.71E-05	1.699	Sjogren syndrome antigen B (autoantigen La)	Nucleus	enzyme
SSH2	5.52E-03	1.889	slingshot homolog 2 (Drosophila)	Cytoplasm	phosphatase
SSPN	2.24E-02	2.448	sarcospan (Kras oncogene-associated gene)	Plasma Membrane	other
ST3GAL1	3.74E-02	2.551	ST3 beta-galactoside alpha-2,3-sialyltransferase 1	Cytoplasm	enzyme
ST7-AS1	4.43E-02	−2.145	ST7 antisense RNA 1 (non-protein coding)	unknown	other
STAT1	8.35E-03	2.408	signal transducer and activator of transcription 1, 91kDa	Nucleus	transcription regulator
STAT5B	1.11E-04	−3.375	signal transducer and activator of transcription 5B	Nucleus	transcription regulator
STK10	2.57E-05	2.579	serine/threonine kinase 10	Cytoplasm	kinase
STK32C	4.27E-03	2.223	serine/threonine kinase 32C	unknown	kinase
STMN4	1.10E-03	2.202	stathmin-like 4	Cytoplasm	other
STX11	1.22E-02	3.358	syntaxin 11	Plasma Membrane	transporter
STX16	2.84E-02	1.592	syntaxin 16	Cytoplasm	transporter
SUN5	3.34E-03	1.814	Sad1 and UNC84 domain containing 5	unknown	other

SUPT16H	1.40E-02	4.056	suppressor of Ty 16 homolog (S. cerevisiae)	Nucleus	transcription regulator
SUSD4	5.69E-03	3.403	sushi domain containing 4	unknown	other
SUV39H2	1.82E-02	1.811	suppressor of variegation 3-9 homolog 2 (Drosophila)	Nucleus	transcription regulator
SUV420H2	4.80E-02	1.679	suppressor of variegation 4-20 homolog 2 (Drosophila)	Nucleus	enzyme
SYNGR1	2.55E-02	−2.174	synaptogyrin 1	Plasma Membrane	transporter
SYT12	3.42E-03	−1.627	synaptotagmin XII	Plasma Membrane	transporter
TACR2	1.61E-02	2.873	tachykinin receptor 2	Plasma Membrane	G-protein coupled receptor
TAF9	2.05E-06	1.65	TAF9 RNA polymerase II, TATA box binding protein (TBP)-associated factor, 32kDa	Nucleus	transcription regulator
TAF7L	2.14E-04	2.36	TAF7-like RNA polymerase II, TATA box binding protein (TBP)-associated factor, 50kDa	Nucleus	transcription regulator
TAGLN2	1.20E-02	1.653	transgelin 2	Cytoplasm	other
TAMM41	1.88E-03	2.019	TAM41, mitochondrial translocator assembly and maintenance protein, homolog (S. cerevisiae)	Cytoplasm	other
TARS2	1.18E-03	2.238	threonyl-tRNA synthetase 2, mitochondrial (putative)	Cytoplasm	enzyme
TAS2R4	3.88E-02	2.335	taste receptor, type 2, member 4	Plasma Membrane	G-protein coupled receptor
TBC1D30	8.83E-04	3.571	TBC1 domain family, member 30	Cytoplasm	other
TBX15	3.82E-03	2.406	T-box 15	Nucleus	transcription regulator
TCEANC2	6.54E-04	2.46	transcription elongation factor A (SII) N-terminal and central domain containing 2	unknown	other
TCP11L2	9.71E-03	−1.602	t-complex 11 (mouse)-like 2	unknown	other
TDP1 (includes EG:104884)	2.04E-03	1.924	tyrosyl-DNA phosphodiesterase 1	Nucleus	enzyme
TEAD2	3.87E-02	−2.789	TEA domain family member 2	Nucleus	transcription regulator
TERF2	3.66E-04	2.044	telomeric repeat binding factor 2	Nucleus	other
TEX101	1.31E-02	−1.713	testis expressed 101	Plasma Membrane	other
TFAM	3.29E-03	1.944	transcription factor A, mitochondrial	Cytoplasm	transcription regulator

TFPI2	1.20E-06	2.222	tissue factor pathway inhibitor 2	Extracellular Space	other
TFPI	2.97E-02	3.225	tissue factor pathway inhibitor (lipoprotein-associated coagulation inhibitor)	Extracellular Space	other
TFR2	4.22E-03	2.22	transferrin receptor 2	Plasma Membrane	transporter
TFRC	3.59E-02	1.842	transferrin receptor (p90, CD71)	Plasma Membrane	transporter
THBS1	1.32E-02	2.078	thrombospondin 1	Extracellular Space	other
THRAP3	1.53E-04	3.788	thyroid hormone receptor associated protein 3	Nucleus	transcription regulator
TIMM13	4.84E-02	2.48	translocase of inner mitochondrial membrane 13 homolog (yeast)	Cytoplasm	transporter
TIMM21	5.32E-05	1.612	translocase of inner mitochondrial membrane 21 homolog (yeast)	Cytoplasm	other
TMCO6	1.38E-05	2.276	transmembrane and coiled-coil domains 6	unknown	other
TMCO5A	6.54E-03	3.112	transmembrane and coiled-coil domains 5A	unknown	other
TMEM17	3.93E-04	1.776	transmembrane protein 17	unknown	other
TMEM158	1.24E-06	3.549	transmembrane protein 158 (gene/pseudogene)	Plasma Membrane	other
TMEM175	1.09E-02	−2.089	transmembrane protein 175	unknown	other
TMEM237	5.55E-03	2.111	transmembrane protein 237	unknown	other
TMEM242	8.30E-06	3.29	transmembrane protein 242	unknown	other
TMEM132E	4.84E-02	−2.286	transmembrane protein 132E	unknown	other
TMEM184A	2.31E-02	−2.343	transmembrane protein 184A	unknown	other
TMEM185A	4.40E-02	−1.89	transmembrane protein 185A	unknown	other
TMEM45A	4.99E-07	−1.546	transmembrane protein 45A	Plasma Membrane	other
TMPRSS13	5.94E-03	−2.357	transmembrane protease, serine 13	unknown	peptidase
TMX3	3.43E-03	3.433	thioredoxin-related transmembrane protein 3	Cytoplasm	enzyme
TNFRSF25	2.92E-04	1.727	tumor necrosis factor receptor superfamily, member 25	Plasma Membrane	transmembrane receptor
TNFSF9	3.26E-05	−1.953	tumor necrosis factor (ligand) superfamily, member 9	Extracellular Space	cytokine
TNS1	3.99E-02	2.001	tensin 1	Plasma Membrane	other
TOB2	2.91E-02	2.145	transducer of ERBB2, 2	Nucleus	other
TOE1	1.93E-04	3.008	target of EGR1, member 1 (nuclear)	Nucleus	other
TOMM5	2.62E-06	1.857	translocase of outer mitochondrial	Cytoplasm	other

			membrane 5 homolog (yeast)		
TOP2A	9.34E-05	2.358	topoisomerase (DNA) II alpha 170kDa	Nucleus	enzyme
TPCN1	4.66E-03	−2.692	two pore segment channel 1	Plasma Membrane	ion channel
TPD52	3.21E-04	1.669	tumor protein D52	Cytoplasm	other
TPK1	6.51E-03	2.027	thiamin pyrophosphokinase 1	Cytoplasm	kinase
TPM3	8.28E-03	2.341	tropomyosin 3	Cytoplasm	other
TPX2	4.44E-05	2.578	TPX2, microtubule-associated, homolog (Xenopus laevis)	Nucleus	other
TRAF1	1.17E-02	2.018	TNF receptor-associated factor 1	Cytoplasm	other
TRMT11	6.46E-06	1.557	tRNA methyltransferase 11 homolog (S. cerevisiae)	unknown	other
TRNP1	2.82E-02	−1.797	TMF1-regulated nuclear protein 1	unknown	other
TRPC5	1.07E-02	3.045	transient receptor potential cation channel, subfamily C, member 5	Plasma Membrane	ion channel
TRPC6	3.65E-02	2.863	transient receptor potential cation channel, subfamily C, member 6	Plasma Membrane	ion channel
TRUB1	2.45E-02	2.039	TruB pseudouridine (psi) synthase homolog 1 (E. coli)	unknown	enzyme
TSEN15	4.52E-05	1.531	tRNA splicing endonuclease 15 homolog (S. cerevisiae)	Nucleus	other
TSFM	1.96E-03	2.405	Ts translation elongation factor, mitochondrial	Cytoplasm	translation regulator
TSLP	4.21E-06	2.559	thymic stromal lymphopoietin	Extracellular Space	cytokine
TTC12	4.54E-06	3.246	tetratricopeptide repeat domain 12	unknown	other
TTC18	3.08E-03	−1.65	tetratricopeptide repeat domain 18	unknown	other
TTC33	4.32E-02	1.855	tetratricopeptide repeat domain 33	unknown	other
TTF2	3.32E-03	1.623	transcription termination factor, RNA polymerase II	Nucleus	transcription regulator
TUBB1	2.92E-03	−1.973	tubulin, beta 1 class VI	Cytoplasm	other
TYROBP	2.35E-02	2.076	TYRO protein tyrosine kinase binding protein	Plasma Membrane	other
U2AF2	7.70E-04	1.607	U2 small nuclear RNA auxiliary factor 2	Nucleus	other
UBE2Q1	1.22E-03	2.464	ubiquitin-conjugating enzyme E2Q family member 1	unknown	enzyme
UBR4	4.77E-02	1.806	ubiquitin protein ligase E3 component n-recognin 4	Nucleus	other
UCP1	6.16E-03	−1.99	uncoupling protein 1 (mitochondrial, proton carrier)	Cytoplasm	transporter
UCP3	3.73E-02	2.846	uncoupling protein 3 (mitochondrial, proton carrier)	Cytoplasm	transporter
UEVLD	5.18E-03	2.031	UEV and lactate/malate dehyrogenase domains	Cytoplasm	enzyme

UGGT1	1.68E-05	−1.669	UDP-glucose glycoprotein glucosyltransferase 1	Cytoplasm	enzyme
UGT2B15	2.06E-04	3.16	UDP glucuronosyltransferase 2 family, polypeptide B15	Cytoplasm	enzyme
UHMK1	1.59E-03	1.581	U2AF homology motif (UHM) kinase 1	Nucleus	kinase
UNC5C	4.52E-02	−1.638	unc-5 homolog C (C. elegans)	Plasma Membrane	transmembrane receptor
UPF3A	3.08E-02	1.616	UPF3 regulator of nonsense transcripts homolog A (yeast)	Nucleus	transporter
USP7	6.28E-03	1.564	ubiquitin specific peptidase 7 (herpes virus-associated)	Nucleus	peptidase
USP22	1.38E-02	−2.5	ubiquitin specific peptidase 22	Nucleus	peptidase
USP24	4.66E-03	1.976	ubiquitin specific peptidase 24	unknown	peptidase
USP9Y	4.72E-02	2.834	ubiquitin specific peptidase 9, Y-linked	Cytoplasm	peptidase
UTP15	1.25E-04	1.592	UTP15, U3 small nucleolar ribonucleoprotein, homolog (S. cerevisiae)	Nucleus	other
VAMP1	4.33E-02	1.61	vesicle-associated membrane protein 1 (synaptobrevin 1)	Plasma Membrane	transporter
VASH1	2.33E-02	−1.637	vasohibin 1	Extracellular Space	other
VAT1L	4.76E-03	−1.68	vesicle amine transport protein 1 homolog (T. californica)-like	unknown	enzyme
VCX2	3.68E-02	2.493	variable charge, X-linked 2	unknown	other
VEGFA	6.88E-05	−1.5	vascular endothelial growth factor A	Extracellular Space	growth factor
VEZT	3.24E-02	1.535	vezatin, adherens junctions transmembrane protein	Plasma Membrane	other
VPRBP	1.98E-02	−1.546	Vpr (HIV-1) binding protein	Nucleus	other
VPS8 (includes EG:209018)	1.56E-04	2.83	vacuolar protein sorting 8 homolog (S. cerevisiae)	unknown	other
VSIG10L	2.93E-04	−2.294	V-set and immunoglobulin domain containing 10 like	unknown	other
VWA3A	1.68E-02	1.939	von Willebrand factor A domain containing 3A	unknown	other
VWA5B1	3.84E-02	−2.915	von Willebrand factor A domain containing 5B1	Extracellular Space	other
WARS2	2.70E-02	1.704	tryptophanyl tRNA synthetase 2, mitochondrial	Cytoplasm	enzyme
WDR4	1.38E-02	2.215	WD repeat domain 4	Nucleus	other
WDR18	2.18E-03	1.558	WD repeat domain 18	Nucleus	other
WDR38	2.13E-02	2.252	WD repeat domain 38	unknown	other
WDR61	1.90E-03	2.52	WD repeat domain 61	unknown	other
WHSC1	1.80E-05	2.83	Wolf-Hirschhorn syndrome	Nucleus	enzyme

			candidate 1		
WISP3	1.18E-02	2.535	WNT1 inducible signaling pathway protein 3	Extracellular Space	growth factor
WNT4	1.97E-03	−2.093	wingless-type MMTV integration site family, member 4	Extracellular Space	cytokine
WNT5A	4.68E-02	−1.629	wingless-type MMTV integration site family, member 5A	Extracellular Space	cytokine
XPO5	3.85E-04	1.538	exportin 5	Nucleus	transporter
YBX1	8.62E-04	1.664	Y box binding protein 1	Nucleus	transcription regulator
YKT6	5.17E-04	1.637	YKT6 v-SNARE homolog (S. cerevisiae)	Cytoplasm	enzyme
YLPM1	1.00E-02	2.449	YLP motif containing 1	Nucleus	other
YRDC	1.59E-04	1.853	yrdC domain containing (E. coli)	unknown	other
ZBTB40	8.33E-03	2.729	zinc finger and BTB domain containing 40	Nucleus	other
ZCCHC4	3.47E-04	1.619	zinc finger, CCHC domain containing 4	unknown	enzyme
ZCCHC7	1.68E-03	1.757	zinc finger, CCHC domain containing 7	Nucleus	other
ZDHHC14	1.50E-04	2.76	zinc finger, DHHC-type containing 14	unknown	other
ZDHHC19	2.88E-03	1.937	zinc finger, DHHC-type containing 19	unknown	other
ZDHHC21	1.11E-03	−1.533	zinc finger, DHHC-type containing 21	Plasma Membrane	enzyme
ZEB2	1.03E-02	−2.772	zinc finger E-box binding homeobox 2	Nucleus	transcription regulator
ZFP1	4.42E-02	4.3	zinc finger protein 1 homolog (mouse)	Nucleus	other
ZFR	2.02E-02	2.318	zinc finger RNA binding protein	Nucleus	other
ZIM3	5.18E-03	3.066	zinc finger, imprinted 3	Nucleus	other
ZMYM6NB	3.62E-03	3.849	ZMYM6 neighbor	unknown	other
ZNF22	5.59E-04	2.042	zinc finger protein 22 (KOX 15)	Nucleus	other
ZNF43	2.74E-02	1.966	zinc finger protein 43	Nucleus	other
ZNF71	1.85E-05	2.186	zinc finger protein 71	Nucleus	other
ZNF80	3.65E-02	−2.405	zinc finger protein 80	Nucleus	transcription regulator
ZNF193	1.18E-03	2.542	zinc finger protein 193	Nucleus	transcription regulator
ZNF202	1.50E-02	2.348	zinc finger protein 202	Nucleus	transcription regulator
ZNF221	1.88E-02	−1.917	zinc finger protein 221	Nucleus	other
ZNF233	2.71E-03	3.756	zinc finger protein 233	Nucleus	other
ZNF318	2.03E-02	2.506	zinc finger protein 318	Nucleus	other
ZNF326	1.07E-04	3.159	zinc finger protein 326	Nucleus	transcription

					regulator
ZNF330	1.50E-03	1.517	zinc finger protein 330	Nucleus	other
ZNF415	7.04E-03	2.584	zinc finger protein 415	Cytoplasm	other
ZNF416	1.84E-02	−2.317	zinc finger protein 416	Nucleus	other
ZNF436	2.72E-03	1.862	zinc finger protein 436	Nucleus	other
ZNF439	3.19E-02	−2.845	zinc finger protein 439	Nucleus	other
ZNF485	2.71E-04	3.961	zinc finger protein 485	Nucleus	other
ZNF493	4.71E-02	2.85	zinc finger protein 493	Nucleus	other
ZNF498	1.08E-04	1.548	zinc finger protein 498	unknown	other
ZNF501	1.83E-02	1.636	zinc finger protein 501	Nucleus	other
ZNF519	5.64E-03	3.101	zinc finger protein 519	Nucleus	other
ZNF589	2.26E-05	2.587	zinc finger protein 589	unknown	other
ZNF605	2.93E-02	2.509	zinc finger protein 605	unknown	other
ZNF609	3.71E-02	−1.779	zinc finger protein 609	unknown	other
ZNF681	1.12E-02	−1.781	zinc finger protein 681	unknown	other
ZNF738	8.44E-03	1.526	zinc finger protein 738	unknown	enzyme
ZNF771	1.84E-02	1.591	zinc finger protein 771	unknown	other
ZNF784	3.03E-03	1.553	zinc finger protein 784	unknown	other
ZNF883	4.12E-02	2.311	zinc finger protein 883	unknown	other
ZNF705G	7.21E-03	4.91	zinc finger protein 705G	unknown	other
ZNF826P	3.96E-04	3.734	zinc finger protein 826, pseudogene	unknown	other
ZWILCH	2.10E-04	1.863	Zwilch, kinetochore associated, homolog (Drosophila)	Nucleus	other

Supplementary Table 4. 121 genes are common to 0 h and 4 h post-heat timepoint. The name, p-value, fold change, location and family of each gene are indicated. Genes were filtered for an absolute value log2 ration ≥1.5 and a significance value of p ≤ 0.05.

**Symbol**	**p-value (0h)**	**Log Ratio (0h)**	**p-value (4h)**	**Log Ratio (4h)**	**Gene Name**	**Location**	**Family**
ADAMTS9-AS2	2.25E-02	−1.544	3.48E-03	−2.355	ADAMTS9 antisense RNA 2 (non-protein coding)	unknown	other
ADCK4	4.20E-03	2.354	2.72E-03	2.573	aarF domain containing kinase 4	Cytoplasm	kinase
ANKRD44	8.59E-03	−3.244	1.01E-02	3.128	ankyrin repeat domain 44	unknown	other
ARHGAP20	2.24E-02	−2.482	3.27E-02	−2.245	Rho GTPase activating protein 20	Cytoplasm	other
ASMTL-AS1	3.33E-02	−1.589	2.05E-02	1.797	ASMTL antisense RNA 1 (non-protein	unknown	other

					coding)		
ATM	1.33E-02	2.471	3.52E-02	2.454	ataxia telangiectasia mutated	Nucleus	kinase
AXIN2	8.46E-04	1.89	2.65E-04	−2.343	axin 2	Cytoplasm	other
BEND5	2.99E-02	1.555	5.19E-03	−2.353	BEN domain containing 5	Cytoplasm	other
C11orf96	3.49E-06	−5.034	1.83E-05	−3.791	chromosome 11 open reading frame 96	unknown	other
C17orf56	8.65E-03	1.804	1.08E-02	1.715	chromosome 17 open reading frame 56	unknown	other
C19orf6	1.25E-03	−2.964	1.96E-02	−3.557	chromosome 19 open reading frame 6	unknown	other
C20orf196	1.06E-02	−2.991	2.46E-02	1.772	chromosome 20 open reading frame 196	unknown	other
C5AR1	1.63E-02	−1.505	6.65E-03	1.847	complement component 5a receptor 1	Plasma Membrane	G-protein coupled receptor
C8orf45	3.69E-02	−2.408	2.32E-02	−2.728	chromosome 8 open reading frame 45	unknown	other
CALHM2	4.75E-02	1.972	2.49E-02	2.36	calcium homeostasis modulator 2	unknown	other
CAMKK2	9.32E-03	2.262	4.44E-02	1.522	calcium/calmodulin-dependent protein kinase kinase 2, beta	Cytoplasm	kinase
CAMTA1	2.38E-03	1.798	4.77E-02	2.631	calmodulin binding transcription activator 1	unknown	other
CARS2	6.32E-03	2.213	2.98E-02	1.528	cysteinyl-tRNA synthetase 2, mitochondrial (putative)	Cytoplasm	enzyme
CCL18	4.04E-02	2.583	4.55E-02	−2.118	chemokine (C-C motif) ligand 18 (pulmonary and activation-regulated)	Extracellular Space	cytokine
CKAP2	2.91E-02	1.577	1.46E-02	2.84	cytoskeleton associated	Cytoplasm	other

					protein 2		
CLDN10	1.19E-03	1.948	1.92E-03	1.774	claudin 10	Plasma Membrane	other
CLTB	1.56E-02	1.962	1.10E-02	2.131	clathrin, light chain B	Plasma Membrane	other
DCN	2.60E-02	1.665	4.82E-02	−2.343	decorin	Extracellular Space	other
DLGAP1	2.70E-02	2.236	4.97E-03	−3.323	discs, large (Drosophila) homolog-associated protein 1	Plasma Membrane	other
DPYD	2.21E-03	−2.5	1.19E-02	1.744	dihydropyrimidine dehydrogenase	Cytoplasm	enzyme
EFCAB1	1.54E-02	−2.234	3.71E-02	−2.489	EF-hand calcium binding domain 1	unknown	other
ENAM	3.33E-02	−1.959	2.18E-02	−2.191	enamelin	Extracellular Space	other
EPHA7	4.83E-02	1.889	3.77E-02	2.272	EPH receptor A7	Plasma Membrane	kinase
EXOC4	2.55E-02	−1.796	1.96E-02	2.119	exocyst complex component 4	Cytoplasm	transporter
FAM124A	6.97E-03	−3.126	7.39E-03	−3.087	family with sequence similarity 124A	unknown	other
FAM46D	5.83E-03	3.621	4.70E-02	2.161	family with sequence similarity 46, member D	unknown	other
FCRL4	8.54E-03	−3.439	4.79E-02	−2.219	Fc receptor-like 4	unknown	other
FGF18	6.03E-03	−2.55	1.61E-02	2.038	fibroblast growth factor 18	Extracellular Space	growth factor
FGF7	4.79E-03	2.143	1.13E-04	4.37	fibroblast growth factor 7	Extracellular Space	growth factor
GEM	1.13E-05	−3.309	2.03E-05	2.99	GTP binding protein overexpressed in skeletal muscle	Plasma Membrane	enzyme
GGA2	3.28E-02	1.999	3.31E-02	1.995	golgi-associated, gamma adaptin	Cytoplasm	transporter

					ear containing, ARF binding protein 2		
GKN1	1.73E-02	−1.833	3.66E-02	−1.506	gastrokine 1	Extracellular Space	growth factor
GLS2	7.96E-03	2.31	3.22E-02	−3.044	glutaminase 2 (liver, mitochondrial)	Cytoplasm	enzyme
GOLGA6A	1.90E-02	2.017	8.91E-03	2.412	golgin A6 family, member A	unknown	other
GPRC6A	4.70E-02	−1.692	1.59E-02	2.257	G protein-coupled receptor, family C, group 6, member A	Plasma Membrane	G-protein coupled receptor
GRAPL	1.43E-02	2.145	2.52E-02	1.861	GRB2-related adaptor protein-like	unknown	other
GRIN1	3.69E-02	−1.989	1.47E-02	2.243	glutamate receptor, ionotropic, N-methyl D-aspartate 1	Plasma Membrane	ion channel
HIST1H4A	6.52E-04	1.895	1.19E-04	2.585	histone cluster 1, H4a	Nucleus	other
HMGA2	8.38E-03	3.825	1.27E-02	3.478	high mobility group AT-hook 2	Nucleus	enzyme
ID2B	1.11E-04	2.298	5.32E-05	2.614	inhibitor of DNA binding 2B, dominant negative helix-loop-helix protein (pseudogene)	unknown	other
IER5L	6.41E-05	2.433	8.40E-05	2.32	immediate early response 5-like	unknown	other
IGFBP1	4.37E-02	−1.692	2.46E-02	−1.982	insulin-like growth factor binding protein 1	Extracellular Space	other
IL6R	4.81E-02	−2.054	2.51E-02	−2.46	interleukin 6 receptor	Plasma Membrane	transmembrane receptor
IPCEF1	3.78E-02	−1.572	4.16E-03	2.656	interaction protein for cytohesin	Cytoplasm	enzyme

					exchange factors 1		
ISYNA1	2.22E-02	−2.603	9.06E-03	2.022	inositol-3-phosphate synthase 1	unknown	enzyme
ITGB8	1.75E-04	2.189	8.44E-04	−1.756	integrin, beta 8	Plasma Membrane	other
JMJD4	2.62E-02	1.808	2.73E-04	2.313	jumonji domain containing 4	unknown	other
JMJD6	1.71E-02	−1.706	1.68E-02	−1.713	jumonji domain containing 6	Plasma Membrane	other
KCNJ13	2.70E-02	2.069	1.47E-02	1.567	potassium inwardly-rectifying channel, subfamily J, member 13	Plasma Membrane	ion channel
KIAA0509	1.45E-02	1.867	1.32E-03	3.102	hypothetical LOC57242	unknown	other
KIAA1430	3.23E-02	3.407	2.58E-02	3.618	KIAA1430	unknown	other
LINC00324	1.78E-03	3.638	2.07E-03	3.53	long intergenic non-protein coding RNA 324	unknown	other
LOC100216546	2.14E-05	−3.658	4.12E-02	2.271	hypothetical LOC100216546	unknown	other
LOC283481	2.05E-02	2.166	1.50E-02	2.337	hypothetical LOC283481	unknown	other
LOC285696	7.57E-03	1.777	1.13E-02	1.625	hypothetical LOC285696	unknown	other
LOC644656	8.33E-03	−3.02	3.90E-02	−2.057	hypothetical LOC644656	unknown	other
LYG1	8.92E-04	1.509	4.90E-04	1.688	lysozyme G-like 1	Extracellular Space	other
MAGEA8	2.75E-03	3.796	9.71E-03	2.9	melanoma antigen family A, 8	unknown	other
MCTP1	2.68E-02	−2.138	2.71E-03	2.499	multiple C2 domains, transmembrane 1	unknown	other
MIR17HG	1.09E-02	−2.82	5.14E-03	-3.326	miR-17-92 cluster host gene (non-protein coding)	unknown	other
MMAA	1.57E-02	1.73	2.39E-02	1.557	methylmalonic aciduria	Cytoplasm	other

					(cobalamin deficiency) cblA type		
MSX1	1.67E-05	2.125	3.96E-05	1.83	msh homeobox 1	Nucleus	transcription regulator
MUC20	3.61E-02	−1.934	5.79E-03	−3.009	mucin 20, cell surface associated	Plasma Membrane	other
NFATC1	2.55E-02	1.65	2.92E-02	−1.847	nuclear factor of activated T-cells, cytoplasmic, calcineurin-dependent 1	Nucleus	transcription regulator
NPAS1	7.11E-04	2.239	1.20E-04	−3.099	neuronal PAS domain protein 1	Nucleus	transcription regulator
NT5DC4	3.44E-02	1.653	2.32E-02	3.092	5'-nucleotidase domain containing 4	unknown	other
NUMA1	1.01E-02	3.587	3.55E-03	−1.841	nuclear mitotic apparatus protein 1	Nucleus	other
NUP210L	3.80E-02	−2.692	7.33E-03	4.036	nucleoporin 210kDa-like	unknown	other
PDYN	2.46E-02	−1.711	2.02E-02	−1.798	prodynorphin	Extracellular Space	transporter
PEX14	2.58E-02	1.931	1.24E-02	2.315	peroxisomal biogenesis factor 14	Cytoplasm	transcription regulator
PPP2R2A	7.22E-04	−2.376	7.36E-04	2.367	protein phosphatase 2, regulatory subunit B, alpha	Cytoplasm	phosphatase
PRDM13	2.42E-03	1.623	2.43E-04	2.506	PR domain containing 13	Nucleus	other
PRPF38B	1.36E-03	−1.835	1.83E-03	−1.73	PRP38 pre-mRNA processing factor 38 (yeast) domain containing B	unknown	other
PYGO1	1.79E-02	2.897	4.36E-02	4.22	pygopus homolog 1 (Drosophila)	Nucleus	other
RAB7A	4.01E-02	3.192	1.87E-02	3.91	RAB7A, member RAS	Cytoplasm	enzyme

					oncogene family		
RABGAP1	5.48E-03	2.131	2.02E-02	1.579	RAB GTPase activating protein 1	Cytoplasm	other
RBFOX3	2.60E-02	−1.78	1.21E-03	2.013	RNA binding protein, fox-1 homolog (C. elegans) 3	Nucleus	other
RBM47	1.01E-02	−1.995	3.92E-04	−1.74	RNA binding motif protein 47	unknown	other
RGS13	1.58E-02	1.9	1.91E-02	1.813	regulator of G-protein signaling 13	Nucleus	other
RGS4	9.77E-03	−2.302	3.66E-02	1.654	regulator of G-protein signaling 4	Cytoplasm	other
RHOBTB2	1.73E-02	−1.645	7.89E-04	3.27	Rho-related BTB domain containing 2	unknown	enzyme
SCN2A	9.20E-03	1.968	1.45E-03	2.891	sodium channel, voltage-gated, type II, alpha subunit	Plasma Membrane	ion channel
SCNN1G	6.22E-03	−3.159	9.90E-04	2.532	sodium channel, nonvoltage-gated 1, gamma	Plasma Membrane	ion channel
SERGEF	1.56E-02	1.537	1.54E-03	2.519	secretion regulating guanine nucleotide exchange factor	unknown	other
SERPIND1	4.69E-02	1.781	4.58E-02	1.793	serpin peptidase inhibitor, clade D (heparin cofactor), member 1	Extracellular Space	other
SGCD	3.47E-02	2.354	4.72E-02	−2.157	sarcoglycan, delta (35kDa dystrophin-associated glycoprotein)	Cytoplasm	other
SHB	4.51E-02	1.643	2.46E-02	1.942	Src homology 2 domain containing adaptor protein B	unknown	other

SHD	4.10E-02	1.943	4.88E-02	1.846	Src homology 2 domain containing transforming protein D	Cytoplasm	other
SLC26A3	2.73E-02	−3.091	1.23E-02	−3.768	solute carrier family 26, member 3	Plasma Membrane	transporter
SLC2A11	3.83E-02	−1.516	5.94E-03	2.386	solute carrier family 2 (facilitated glucose transporter), member 11	Plasma Membrane	other
SLC4A4	7.26E-03	1.79	1.47E-02	1.524	solute carrier family 4, sodium bicarbonate cotransporter, member 4	Plasma Membrane	transporter
SMCHD1	2.07E-02	2.971	2.40E-02	2.862	structural maintenance of chromosomes flexible hinge domain containing 1	Nucleus	other
SMPD3	7.20E-04	−1.948	2.08E-04	2.449	sphingomyelin phosphodiesterase 3, neutral membrane (neutral sphingomyelinase II)	Cytoplasm	enzyme
SNCAIP	2.28E-02	1.996	3.67E-02	−1.757	synuclein, alpha interacting protein	Cytoplasm	transcription regulator
SNX24	1.93E-02	2.677	3.40E-02	1.859	sorting nexin 24	unknown	transporter
SPAG4	3.49E-03	3.477	2.34E-02	2.256	sperm associated antigen 4	Cytoplasm	other
SPAG5-AS1	8.37E-04	2.871	2.57E-03	2.308	SPAG5 antisense RNA 1 (non-protein coding)	unknown	other
SPATA6	2.87E-02	1.931	1.12E-02	2.437	spermatogenesis associated 6	unknown	other
SRSF3	4.97E-02	−2.048	1.53E-03	3.891	serine/arginine-	Nucleus	other

					rich splicing factor 3		
TAF6L	2.35E-02	1.59	3.24E-02	2.334	TAF6-like RNA polymerase II, p300/CBP-associated factor (PCAF)-associated factor, 65kDa	Nucleus	transcription regulator
TBC1D2B	2.57E-03	1.835	2.28E-02	−1.501	TBC1 domain family, member 2B	unknown	other
THRA	1.85E-02	1.792	1.73E-02	1.823	thyroid hormone receptor, alpha	Nucleus	ligand-dependent nuclear receptor
TIAM1	2.19E-02	2.036	1.95E-04	−1.947	T-cell lymphoma invasion and metastasis 1	Cytoplasm	other
TLR2	2.27E-03	1.815	3.00E-03	−1.716	toll-like receptor 2	Plasma Membrane	transmembrane receptor
TMEM132B	9.76E-03	−3.713	1.16E-02	−3.569	transmembrane protein 132B	unknown	other
TRAF3IP2-AS1	3.04E-03	4.26	3.76E-02	2.366	TRAF3IP2 antisense RNA 1 (non-protein coding)	unknown	other
TRPM1	8.24E-03	3.261	1.18E-02	3.008	transient receptor potential cation channel, subfamily M, member 1	Plasma Membrane	ion channel
UTS2	4.88E-02	−3.57	2.02E-02	4.542	urotensin 2	Extracellular Space	other
VSTM2L	1.22E-02	−2.419	4.83E-02	1.689	V-set and transmembrane domain containing 2 like	unknown	other
WFDC2	4.27E-02	−1.512	1.67E-07	−1.54	WAP four-disulfide core domain 2	Extracellular Space	other
WNT10B	3.96E-02	−1.912	2.13E-02	−2.255	wingless-type MMTV	Extracellular Space	other

					integration site family, member 10B		
ZFHX2	8.15E-03	−1.703	1.61E-03	−2.386	zinc finger homeobox 2	Nucleus	other
ZG16	3.71E-04	1.872	5.07E-05	2.672	zymogen granule protein 16 homolog (rat)	Extracellular Space	other
ZMAT1	1.63E-04	2.545	2.51E-05	3.537	zinc finger, matrin-type 1	Nucleus	other
ZNF132	1.00E-02	3.217	2.00E-02	-2.727	zinc finger protein 132	Nucleus	transcription regulator
ZNF559	1.22E-04	1.713	3.43E-02	2.506	zinc finger protein 559	Nucleus	other

Supplementary Table 5. 107 genes are common to 0 h and 24 h post-heat timepoint. The name, p-value, fold change, location and family of each gene are indicated. Genes were filtered for an absolute value log2 ration ≥1.5 and a significance value of p ≤ 0.05.

**Symbol**	**p-value (0h)**	**Log Ratio (0h)**	**p-value (24h)**	**Log Ratio (24h)**	**Gene Name**	**Location**	**Family**
ADAM22	4.16E-02	−3.202	2.96E-02	1.934	ADAM metallopeptidase domain 22	Plasma Membrane	peptidase
ADCY1	1.08E-03	−1.864	1.22E-03	−1.819	adenylate cyclase 1 (brain)	Plasma Membrane	enzyme
ALX3	2.10E-02	2.186	1.76E-02	2.285	ALX homeobox 3	Nucleus	transcription regulator
AMH	2.19E-03	−2.145	5.08E-03	−1.804	anti-Mullerian hormone	Extracellular Space	growth factor
APOL6	2.60E-02	2.108	2.46E-02	−2.136	apolipoprotein L, 6	Extracellular Space	transporter
ARPC4	3.68E-02	1.706	1.49E-02	2.155	actin related protein 2/3 complex, subunit 4, 20kDa	unknown	other
ASTN1	2.29E-02	−1.989	1.76E-03	3.495	astrotactin 1	unknown	other
ATF7IP	6.58E-03	2.219	1.46E-03	1.668	activating transcription factor 7 interacting protein	Nucleus	transcription regulator
ATOH8	2.69E-02	2.163	1.71E-05	2.877	atonal homolog 8 (Drosophila)	unknown	other
ATR	2.61E-02	−1.782	5.28E-05	2.007	ataxia telangiectasia and Rad3 related	Nucleus	kinase
BDP1	3.79E-02	3.383	4.54E-02	3.211	B double prime 1, subunit of RNA	Nucleus	transcription regulator

					polymerase III transcription initiation factor IIIB		
CACHD1	1.22E-02	−1.722	2.11E-03	−1.532	cache domain containing 1	unknown	other
CAPS2	4.18E-03	−3.125	1.18E-02	−2.487	calcyphosine 2	unknown	other
CEACAM1	2.73E-04	2.821	4.61E-03	1.834	carcinoembryonic antigen-related cell adhesion molecule 1 (biliary glycoprotein)	Plasma Membrane	transmembrane receptor
CIB3	3.54E-02	−2.108	1.82E-02	−2.511	calcium and integrin binding family member 3	unknown	other
CLEC10A	4.77E-03	1.761	5.28E-04	2.714	C-type lectin domain family 10, member A	Plasma Membrane	other
COL12A1	8.18E-06	3.534	2.97E-04	1.882	collagen, type XII, alpha 1	Extracellular Space	other
CPM	2.47E-03	2.828	2.84E-03	−1.673	carboxypeptidase M	Plasma Membrane	peptidase
CTNND2	2.19E-03	2.411	9.87E-03	1.752	catenin (cadherin-associated protein), delta 2 (neural plakophilin-related arm-repeat protein)	Plasma Membrane	other
CXCL2	3.87E-03	−4.866	9.93E-07	2.874	chemokine (C-X-C motif) ligand 2	Extracellular Space	cytokine
CYP3A43	1.27E-06	−1.896	2.42E-06	1.699	cytochrome P450, family 3, subfamily A, polypeptide 43	Cytoplasm	enzyme
CYP4F11	2.26E-02	−2.568	2.32E-02	2.552	cytochrome P450, family 4, subfamily F, polypeptide 11	Cytoplasm	enzyme
DAB1	1.06E-04	2.349	3.26E-05	2.889	disabled homolog 1 (Drosophila)	Cytoplasm	other
DHX35	2.56E-02	−1.638	4.72E-03	−2.426	DEAH (Asp-Glu-Ala-His) box polypeptide 35	unknown	enzyme
DPP6	1.87E-04	4.217	1.08E-02	2.402	dipeptidyl-peptidase 6	Plasma Membrane	peptidase
ERG	5.38E-03	−2.247	2.07E-02	2.721	v-ets erythroblastosis virus E26 oncogene homolog (avian)	Nucleus	transcription regulator
FAM81B	3.92E-02	1.884	1.05E-02	2.627	family with sequence similarity	unknown	other

					81, member B		
FBLN7	2.82E-04	2.124	3.05E-05	−3.149	fibulin 7	Extracellular Space	other
FBXO9	3.72E-03	1.862	2.81E-03	1.973	F-box protein 9	Cytoplasm	enzyme
FLJ41484	6.42E-03	2.101	6.82E-04	3.29	hypothetical LOC650669	unknown	other
FONG	2.95E-02	−1.742	7.51E-03	−2.424	hypothetical LOC348751	unknown	other
FRMPD4	4.47E-02	2.701	4.53E-02	2.69	FERM and PDZ domain containing 4	unknown	other
GABRB3	2.54E-02	2.505	4.95E-02	2.08	gamma-aminobutyric acid (GABA) A receptor, beta 3	Plasma Membrane	ion channel
GAD1	1.25E-02	−1.582	1.37E-03	2.521	glutamate decarboxylase 1 (brain, 67kDa)	Cytoplasm	enzyme
GH2	4.23E-02	2.888	3.34E-02	3.087	growth hormone 2	Extracellular Space	other
GPR37	4.77E-02	1.644	4.98E-02	2.092	G protein-coupled receptor 37 (endothelin receptor type B-like)	Plasma Membrane	G-protein coupled receptor
GPR61	9.38E-04	2.862	4.86E-02	1.944	G protein-coupled receptor 61	Plasma Membrane	G-protein coupled receptor
GRRP1	3.43E-02	−2.046	2.67E-02	−2.189	glycine/arginine rich protein 1	unknown	other
GUCY1B3	5.65E-06	4.47	3.75E-04	2.141	guanylate cyclase 1, soluble, beta 3	Cytoplasm	enzyme
HECA	3.80E-02	−1.63	2.89E-02	−1.757	headcase homolog (Drosophila)	unknown	other
HERC2	1.25E-02	2.815	4.91E-02	1.968	hect domain and RLD 2	Cytoplasm	enzyme
HNRNPAB	9.32E-03	−2.714	5.05E-03	−3.104	heterogeneous nuclear ribonucleoprotein A/B	Nucleus	enzyme
HP09025	2.10E-02	−2.233	1.74E-02	−2.341	hypothetical protein HP09025	unknown	other
IFT80	3.27E-03	3.892	1.51E-02	2.78	intraflagellar transport 80 homolog (Chlamydomonas)	unknown	other
IL31RA	2.19E-04	3.719	2.75E-03	2.302	interleukin 31 receptor A	Plasma Membrane	transmembrane receptor

IQSEC3	5.52E-03	1.541	3.03E-03	1.746	IQ motif and Sec7 domain 3	Cytoplasm	other
KIAA1161	1.73E-02	2.188	4.32E-02	1.717	KIAA1161	Nucleus	other
KLF2	2.47E-02	−2.92	2.37E-03	−2.016	Kruppel-like factor 2 (lung)	Nucleus	transcription regulator
KLHL25	2.46E-04	−2.763	4.12E-03	−1.608	kelch-like 25 (Drosophila)	Cytoplasm	other
KRTAP19-1	9.14E-05	−3.124	1.26E-03	1.928	keratin associated protein 19-1	unknown	other
LAMA2	3.46E-02	4.019	2.81E-05	1.804	laminin, alpha 2	Extracellular Space	other
LILRA6	2.51E-02	−1.654	3.38E-02	−1.528	leukocyte immunoglobulin-like receptor, subfamily A (with TM domain), member 6	unknown	other
LINC00256B	4.28E-02	1.744	2.43E-02	−2.037	long intergenic non-protein coding RNA 256B	unknown	other
LOC100505633	1.31E-02	2.133	3.36E-06	2.095	hypothetical LOC100505633	unknown	other
LOC100506388	2.46E-02	1.671	1.47E-02	1.899	hypothetical LOC100506388	unknown	other
LOC100507300	4.11E-02	2.916	6.00E-03	−1.761	hypothetical LOC100507300	unknown	other
LOC100509864	4.89E-02	−1.821	1.86E-03	3.904	transmembrane phosphoinositide 3-phosphatase and tensin homolog 2 pseudogene 2	unknown	other
LOC283089	2.02E-02	1.598	6.38E-03	2.089	hypothetical LOC283089	unknown	other
LRRC23	3.55E-03	2.339	2.42E-02	1.51	leucine rich repeat containing 23	Plasma Membrane	other
LST1	1.43E-03	1.862	1.25E-02	1.665	leukocyte specific transcript 1	Plasma Membrane	other
MAGEC2	1.08E-02	1.99	5.19E-03	−2.34	melanoma antigen family C, 2	Plasma Membrane	other
MCF2L	1.35E-02	−2.653	3.99E-02	−2.007	MCF.2 cell line derived transforming sequence-like	Cytoplasm	other
MECOM	2.79E-02	−2.28	6.35E-03	1.745	MDS1 and EVI1 complex locus	Nucleus	transcription regulator
MGC12760	3.31E-04	2.157	1.71E-04	2.433	ciliary rootlet coiled-coil,	unknown	other

					rootletin pseudogene 2		
NOL4	1.55E-02	−2.604	6.82E-03	3.143	nucleolar protein 4	Nucleus	other
NR4A2	2.75E-02	−1.782	4.51E-02	1.552	nuclear receptor subfamily 4, group A, member 2	Nucleus	ligand-dependent nuclear receptor
NRARP	1.38E-04	−2.979	1.81E-03	-1.846	NOTCH-regulated ankyrin repeat protein	Nucleus	transcription regulator
NTRK3	2.77E-02	1.606	1.94E-03	2.052	neurotrophic tyrosine kinase, receptor, type 3	Plasma Membrane	kinase
OFCC1	1.28E-03	−3.174	2.56E-02	1.648	orofacial cleft 1 candidate 1	Cytoplasm	other
OGFR	1.27E-02	1.97	3.06E-02	1.582	opioid growth factor receptor	Plasma Membrane	other
PACSIN3	4.65E-02	−1.551	1.08E-02	2.834	protein kinase C and casein kinase substrate in neurons 3	Cytoplasm	other
PADI4	3.85E-02	1.536	3.17E-02	1.621	peptidyl arginine deiminase, type IV	Cytoplasm	enzyme
PARD3B	7.74E-03	2.541	3.72E-04	1.737	par-3 partitioning defective 3 homolog B (C. elegans)	Plasma Membrane	other
PCF11 (human)	1.09E-03	−1.677	1.26E-03	−1.63	PCF11, cleavage and polyadenylation factor subunit, homolog (S. cerevisiae)	Nucleus	other
PMS1	3.15E-02	1.768	2.40E-02	2.026	PMS1 postmeiotic segregation increased 1 (S. cerevisiae)	Nucleus	enzyme
PSMD6	3.05E-02	−1.824	1.56E-02	2.164	proteasome (prosome, macropain) 26S subunit, non-ATPase, 6	Cytoplasm	enzyme
RFC3	1.28E-02	−2.344	6.53E-05	1.886	replication factor C (activator 1) 3, 38kDa	Nucleus	enzyme
RGN	1.42E-03	1.684	5.68E-05	3.036	regucalcin (senescence marker	Nucleus	enzyme

					protein-30)		
RNF213	7.71E-03	1.787	3.76E-02	2.857	ring finger protein 213	Plasma Membrane	other
RPL10	2.35E-02	1.987	4.34E-06	1.858	ribosomal protein L10	Cytoplasm	other
SEPP1	9.48E-03	2.887	1.83E-02	2.472	selenoprotein P, plasma, 1	Extracellular Space	other
SEZ6L	2.76E-02	2.06	3.50E-02	1.714	seizure related 6 homolog (mouse)-like	Plasma Membrane	other
SFSWAP	7.89E-04	−2.434	1.61E-02	2.937	splicing factor, suppressor of white-apricot homolog (Drosophila)	Nucleus	other
SLC16A5	2.21E-02	1.558	2.03E-02	1.592	solute carrier family 16, member 5 (monocarboxylic acid transporter 6)	Plasma Membrane	transporter
SLC25A31	4.77E-03	2.883	3.38E-02	1.811	solute carrier family 25 (mitochondrial carrier; adenine nucleotide translocator), member 31	Cytoplasm	transporter
SLC27A5	3.50E-03	−2.353	1.66E-04	2.439	solute carrier family 27 (fatty acid transporter), member 5	Cytoplasm	transporter
SLC7A13	2.94E-02	−1.749	2.42E-02	1.841	solute carrier family 7 (anionic amino acid transporter), member 13	Plasma Membrane	transporter
SOCS3	1.08E-03	−2.092	5.42E-03	1.51	suppressor of cytokine signaling 3	Cytoplasm	other
SRC	1.86E-04	−2.286	1.47E-02	2.771	v-src sarcoma (Schmidt-Ruppin A-2) viral oncogene homolog (avian)	Cytoplasm	kinase
ST6GAL1	2.20E-02	−2.595	1.42E-03	1.642	ST6 beta-galactosamide alpha-2,6-sialyltranferase 1	Cytoplasm	enzyme
STK31	5.01E-03	2.266	1.64E-02	1.734	serine/threonine kinase 31	Cytoplasm	kinase
STXBP4	3.02E-02	2.523	1.21E-02	−3.172	syntaxin binding protein 4	Cytoplasm	other

SULT1C2	3.17E-02	2.38	3.62E-02	3.384	sulfotransferase family, cytosolic, 1C, member 2	Cytoplasm	enzyme
TAOK2	4.86E-04	1.966	3.71E-05	3.115	TAO kinase 2	Cytoplasm	kinase
TAPT1	1.62E-02	−2.227	3.66E-02	−1.803	transmembrane anterior posterior transformation 1	Plasma Membrane	G-protein coupled receptor
TASP1	2.65E-02	1.598	3.39E-04	3.983	taspase, threonine aspartase, 1	Nucleus	peptidase
TEX11	3.20E-02	1.594	3.98E-02	−2.631	testis expressed 11	Nucleus	other
TLR8-AS1	1.23E-02	−1.804	2.11E-02	1.583	TLR8 antisense RNA 1 (non-protein coding)	unknown	other
TRDN	3.07E-02	2.404	6.94E-03	3.441	triadin	Cytoplasm	other
TTC39C	4.37E-02	1.909	1.09E-04	−1.56	tetratricopeptide repeat domain 39C	unknown	other
VWA3B	2.80E-02	1.979	5.73E-03	2.816	von Willebrand factor A domain containing 3B	unknown	other
WDR1	1.71E-05	2.453	4.09E-03	1.808	WD repeat domain 1	Extracellular Space	other
ZNF277	1.07E-06	4.714	1.45E-02	2.136	zinc finger protein 277	Nucleus	transcription regulator
ZNF366	1.02E-03	3.019	1.64E-02	1.677	zinc finger protein 366	Nucleus	other
ZNF483	1.77E-03	1.939	3.18E-03	1.723	zinc finger protein 483	Nucleus	transcription regulator
ZNF81	2.57E-03	2.395	4.72E-02	2.45	zinc finger protein 81	Nucleus	transcription regulator
ZNF92	7.11E-03	2.788	4.63E-03	3.057	zinc finger protein 92	Nucleus	transcription regulator

Supplementary Table 6. 183 genes are common to 4 h and 24 h post-heat timepoint. The name, p-value, fold change, location and family of each gene are indicated. Genes were filtered for an absolute value log2 ration ≥1.5 and a significance value of p ≤ 0.05.

**Symbol**	**p-value (4h)**	**Log Ratio (4h)**	**p-value (24h)**	**Log Ratio (24h)**	**Gene Name**	**Location**	**Family**
SULT4A1	1.15E-03	3.532	8.11E-03	2.365	sulfotransferase family 4A, member 1	Cytoplasm	enzyme
ABHD1	1.69E-04	3.493	9.95E-04	2.518	abhydrolase domain containing 1	unknown	enzyme
ACSM3	1.43E-03	2.385	1.23E-02	1.514	acyl-CoA synthetase	Cytoplasm	enzyme

					medium-chain family member 3		
ADAMTS10	2.87E-02	2.028	4.09E-03	−2.547	ADAM metallopeptidase with thrombospondin type 1 motif, 10	Extracellular Space	peptidase
ADAMTS5	3.15E-02	1.803	4.70E-02	1.609	ADAM metallopeptidase with thrombospondin type 1 motif, 5	Extracellular Space	peptidase
ANKRD31	1.31E-02	1.931	9.35E-03	−2.088	ankyrin repeat domain 31	unknown	other
ANKRD33B	1.95E-03	−2.798	1.73E-03	2.863	ankyrin repeat domain 33B	unknown	other
ANKRD9	1.36E-02	−2.354	1.06E-02	−2.496	ankyrin repeat domain 9	unknown	other
ANO6	3.84E-02	2.802	1.39E-02	3.641	anoctamin 6	Plasma Membrane	other
APOBEC3A	8.83E-05	−2.226	4.43E-04	1.665	apolipoprotein B mRNA editing enzyme, catalytic polypeptide-like 3A	Cytoplasm	enzyme
APOC2	1.84E-03	−2.841	1.05E-02	1.635	apolipoprotein C-II	Extracellular Space	transporter
ARHGAP26	4.31E-02	3.01	1.56E-03	2.639	Rho GTPase activating protein 26	Cytoplasm	other
ARPM1	3.35E-04	2.133	3.77E-05	3.147	actin related protein M1	unknown	other
ATF6B	2.06E-02	2.989	4.31E-02	2.142	activating transcription factor 6 beta	Nucleus	transcription regulator
ATF7IP2	7.25E-04	2.019	2.10E-03	1.643	activating transcription factor 7 interacting protein 2	Nucleus	other
B3GALNT1	7.50E-03	3.091	6.10E-03	2.15	beta-1,3-N-acetylgalactosaminyltransferase 1 (globoside blood group)	Cytoplasm	enzyme
B3GALNT2	4.70E-03	−1.942	7.81E-03	1.74	beta-1,3-N-acetylgalactosaminyltransferase 2	unknown	enzyme

BBS4	1.87E-04	2.499	3.58E-05	3.345	Bardet-Biedl syndrome 4	Cytoplasm	other
BCL2L13	6.53E-06	2.248	1.00E-02	1.94	BCL2-like 13 (apoptosis facilitator)	Cytoplasm	other
BMP2K	1.04E-03	−1.675	1.20E-03	−1.629	BMP2 inducible kinase	Nucleus	kinase
C12orf60	1.03E-03	1.772	4.33E-04	2.084	chromosome 12 open reading frame 60	unknown	other
C17orf51	1.83E-03	2.716	3.12E-03	2.44	chromosome 17 open reading frame 51	unknown	other
CACNB2	4.34E-05	4.243	1.59E-02	-2.859	calcium channel, voltage-dependent, beta 2 subunit	Plasma Membrane	ion channel
CALCA	1.16E-02	1.839	3.83E-03	2.344	calcitonin-related polypeptide alpha	Plasma Membrane	other
CCDC103	3.50E-02	1.887	4.35E-02	1.773	coiled-coil domain containing 103	unknown	other
CCDC149	1.94E-03	2.532	6.94E-04	2.515	coiled-coil domain containing 149	unknown	other
CCDC150	4.52E-03	2.09	6.97E-03	1.904	coiled-coil domain containing 150	unknown	other
CCDC8	2.33E-05	2.61	7.35E-05	2.136	coiled-coil domain containing 8	Plasma Membrane	other
CCDC88A	3.59E-02	1.775	4.51E-03	1.507	coiled-coil domain containing 88A	Cytoplasm	other
CCL20	2.47E-05	−2.239	5.31E-05	1.96	chemokine (C-C motif) ligand 20	Extracellular Space	cytokine
CCL26	4.90E-02	2.091	7.01E-03	3.409	chemokine (C-C motif) ligand 26	Extracellular Space	cytokine
CD19	1.01E-02	−2.049	1.91E-02	1.762	CD19 molecule	Plasma Membrane	other
CDRT1	9.09E-03	2.214	1.51E-02	1.966	CMT1A duplicated region transcript 1	unknown	other
CELF5	6.82E-05	2.117	5.28E-03	3	CUGBP, Elav-like family member 5	unknown	other
CHAF1B	1.73E-02	2.787	2.35E-02	2.579	chromatin assembly factor 1, subunit B (p60)	Nucleus	other
CLU	1.20E-02	2.071	1.86E-05	1.74	clusterin	Extracellular Space	other
CNTLN	3.54E-04	1.704	1.79E-04	1.927	centlein, centrosomal protein	unknown	other

COL2A1	1.39E-03	3.021	9.33E-04	2.085	collagen, type II, alpha 1	Extracellular Space	other
COX4I1	3.28E-02	−2.144	3.92E-02	2.04	cytochrome c oxidase subunit IV isoform 1	Cytoplasm	enzyme
COX6A1	6.41E-04	−3.348	2.83E-04	1.586	cytochrome c oxidase subunit VIa polypeptide 1	Cytoplasm	enzyme
CSGALNACT1	4.93E-02	−1.599	3.40E-02	−1.78	chondroitin sulfate N-acetylgalactosaminyltransferase 1	Cytoplasm	enzyme
CYCS	9.23E-04	1.635	3.33E-04	1.977	cytochrome c, somatic	Cytoplasm	enzyme
CYP39A1	3.70E-02	1.803	3.32E-04	−1.875	cytochrome P450, family 39, subfamily A, polypeptide 1	Cytoplasm	enzyme
DDX49	2.43E-02	2.086	4.25E-02	1.791	DEAD (Asp-Glu-Ala-Asp) box polypeptide 49	Nucleus	enzyme
DEPDC4	8.17E-03	3.568	3.08E-02	2.583	DEP domain containing 4	unknown	other
DICER1	4.47E-02	2.157	3.51E-03	2.772	dicer 1, ribonuclease type III	Cytoplasm	enzyme
DNAJB4	4.21E-06	2.636	1.38E-05	2.151	DnaJ (Hsp40) homolog, subfamily B, member 4	Nucleus	other
DNAJC9	3.38E-03	3.064	3.71E-03	3.007	DnaJ (Hsp40) homolog, subfamily C, member 9	unknown	other
EDA	1.97E-02	1.742	2.00E-02	2.609	ectodysplasin A	Extracellular Space	cytokine
EDN2	7.29E-05	4.291	1.71E-03	2.399	endothelin 2	Extracellular Space	growth factor
EFCAB6	1.87E-02	2.486	9.84E-03	−2.036	EF-hand calcium binding domain 6	unknown	other
ELMOD1	1.18E-03	1.802	5.39E-04	−2.089	ELMO/CED-12 domain containing 1	unknown	other
EMCN	3.29E-02	−1.749	1.60E-02	2.901	endomucin	Extracellular Space	other
EMP1	1.15E-04	−1.63	7.07E-06	1.542	epithelial membrane protein	Plasma Membrane	other

					1		
EXOSC2	6.12E-03	3.058	9.84E-03	2.752	exosome component 2	Nucleus	enzyme
EXOSC3	9.07E-04	1.547	3.06E-02	1.643	exosome component 3	Nucleus	enzyme
FAHD2A	5.42E-03	−2.556	1.09E-03	−3.53	fumarylacetoacetate hydrolase domain containing 2A	Cytoplasm	enzyme
FAM125B	1.24E-02	2.311	3.56E-02	1.77	family with sequence similarity 125, member B	unknown	other
FAM26E	3.96E-02	1.565	6.04E-03	-2.477	family with sequence similarity 26, member E	unknown	other
FAM46A	3.59E-06	3.621	5.06E-04	1.519	family with sequence similarity 46, member A	unknown	other
FLJ37638	2.88E-03	−1.724	3.36E-04	2.602	hypothetical LOC400660	unknown	other
FUZ	1.23E-02	1.595	6.28E-03	−1.856	fuzzy homolog (Drosophila)	Plasma Membrane	other
FXR1	3.54E-03	1.841	7.77E-03	1.557	fragile X mental retardation, autosomal homolog 1	Cytoplasm	other
FXYD2	1.24E-03	3.753	1.88E-02	2.097	FXYD domain containing ion transport regulator 2	Plasma Membrane	ion channel
GADD45B	2.09E-03	1.992	1.35E-06	1.801	growth arrest and DNA-damage-inducible, beta	Cytoplasm	other
GFER	4.18E-04	2.471	1.64E-04	2.927	growth factor, augmenter of liver regeneration	Nucleus	other
GNL1	1.35E-02	2.172	8.79E-04	1.772	guanine nucleotide binding protein-like 1	unknown	other
GPR88	4.85E-02	2.474	1.44E-02	3.412	G protein-coupled receptor 88	Plasma Membrane	G-protein coupled receptor
HHIP	2.62E-02	−2.636	3.64E-02	2.411	hedgehog interacting protein	Plasma Membrane	other
HIST1H3A	2.73E-02	2.794	7.78E-03	1.774	histone cluster 1, H3a	Nucleus	other
HNRNPC	6.99E-05	−1.656	3.23E-03	1.869	heterogeneous nuclear	Nucleus	other

					ribonucleoprotein C (C1/C2)		
ICAM1	1.84E-03	−1.764	1.34E-04	2.167	intercellular adhesion molecule 1	Plasma Membrane	transmembrane receptor
ID4	2.02E-02	1.637	2.23E-02	1.598	inhibitor of DNA binding 4, dominant negative helix-loop-helix protein	Nucleus	transcription regulator
IL8	8.78E-06	−1.8	1.65E-06	2.392	interleukin 8	Extracellular Space	cytokine
INSL4	5.29E-04	3.375	1.87E-02	1.606	insulin-like 4 (placenta)	Extracellular Space	other
IRF6	6.34E-05	−2.23	9.60E-03	1.622	interferon regulatory factor 6	Nucleus	transcription regulator
ITFG2	1.32E-02	2.702	5.81E-03	3.384	integrin alpha FG-GAP repeat containing 2	unknown	other
ITGA2B	1.86E-02	−2.409	4.17E-02	1.944	integrin, alpha 2b (platelet glycoprotein IIb of IIb/IIIa complex, antigen CD41)	Plasma Membrane	transmembrane receptor
KLF12	1.67E-03	−2.415	1.27E-02	1.685	Kruppel-like factor 12	Nucleus	transcription regulator
KRT19 (human)	2.21E-02	1.509	4.04E-03	2.221	keratin 19	Cytoplasm	other
KRTAP3-2	1.97E-02	1.853	2.29E-04	4.602	keratin associated protein 3-2	unknown	other
LINC00476	1.46E-03	2.533	7.57E-04	2.874	long intergenic non-protein coding RNA 476	unknown	other
LINC00485	6.43E-03	−4.088	2.23E-02	3.057	long intergenic non-protein coding RNA 485	unknown	other
LIPJ	4.65E-02	2.687	1.41E-02	3.674	lipase, family member J	unknown	other
LMOD3	3.83E-03	3.486	1.04E-02	2.804	leiomodin 3 (fetal)	unknown	other
LOC100130522	1.30E-03	1.585	3.18E-05	2.771	hypothetical LOC100130522	unknown	other
LOC285957	3.73E-02	−1.673	2.47E-02	−1.869	hypothetical protein LOC285957	unknown	other
LOC374443	3.40E-04	3.698	2.15E-02	1.565	CLR pseudogene	unknown	other
LOC388210	3.86E-02	−1.562	1.43E-02	−2.019	apolipophorins-like	unknown	other
LPP	1.33E-04	2.018	1.43E-02	1.659	LIM domain containing	Nucleus	other

					preferred translocation partner in lipoma		
LRRFIP2	1.50E-02	1.758	8.41E-03	2.01	leucine rich repeat (in FLII) interacting protein 2	unknown	other
MAOB	6.88E-04	−2.309	4.65E-03	−1.582	monoamine oxidase B	Cytoplasm	enzyme
MAP2K5	3.38E-02	1.828	1.93E-02	−2.118	mitogen-activated protein kinase kinase 5	Cytoplasm	kinase
MEGF10	1.49E-02	1.804	2.07E-02	−1.665	multiple EGF-like-domains 10	Plasma Membrane	other
MGC16121	1.27E-03	3.798	4.63E-02	−1.671	hypothetical protein MGC16121	unknown	other
MMP19	3.44E-02	1.945	2.68E-02	2.08	matrix metallopeptidase 19	Extracellular Space	peptidase
MMP3	9.28E-05	1.66	5.51E-06	2.707	matrix metallopeptidase 3 (stromelysin 1, progelatinase)	Extracellular Space	peptidase
MTFP1	2.09E-02	1.537	1.76E-02	1.604	mitochondrial fission process 1	Cytoplasm	other
MYEF2	3.38E-04	1.537	6.23E-05	2.079	myelin expression factor 2	Nucleus	transcription regulator
MYLK3	1.18E-03	2.926	4.90E-02	1.842	myosin light chain kinase 3	Cytoplasm	kinase
N6AMT1	2.26E-02	2.41	1.12E-02	2.859	N-6 adenine-specific DNA methyltransferase 1 (putative)	unknown	enzyme
NAALAD2	1.09E-02	4.081	2.49E-02	1.605	N-acetylated alpha-linked acidic dipeptidase 2	Plasma Membrane	peptidase
NAP1L2	4.80E-05	1.615	6.03E-07	3.411	nucleosome assembly protein 1-like 2	Nucleus	other
NAPEPLD	1.54E-03	2.369	3.57E-02	1.641	N-acyl phosphatidylethanolamine phospholipase D	Cytoplasm	enzyme
NDUFS4	1.21E-02	−2.833	1.50E-02	−2.694	NADH dehydrogenase (ubiquinone) Fe-S protein 4, 18kDa	Cytoplasm	enzyme

					(NADH-coenzyme Q reductase)		
NFIB	2.31E-02	3.317	1.01E-03	1.507	nuclear factor I/B	Nucleus	transcription regulator
NKAPL	7.02E-05	4.501	7.13E-04	2.26	NFKB activating protein-like	unknown	other
NMT1	1.78E-03	3.496	2.51E-02	1.905	N-myristoyltransferase 1	Cytoplasm	enzyme
NRP2	2.07E-02	1.561	6.08E-03	1.566	neuropilin 2	Plasma Membrane	kinase
NUFIP1	4.76E-03	1.538	1.03E-04	3.186	nuclear fragile X mental retardation protein interacting protein 1	Nucleus	other
PALLD	4.63E-03	1.843	1.16E-03	2.432	palladin, cytoskeletal associated protein	Cytoplasm	other
PCBP3	4.22E-02	−2.211	1.14E-02	3.092	poly(rC) binding protein 3	Nucleus	other
PCDH1	1.22E-02	−1.616	1.93E-02	1.944	protocadherin 1	Plasma Membrane	other
PCDHB5	6.07E-03	−2.895	1.19E-02	−2.491	protocadherin beta 5	Plasma Membrane	other
PCSK5	9.93E-03	3.957	1.39E-03	−1.897	proprotein convertase subtilisin/kexin type 5	Extracellular Space	peptidase
PDE1C	6.59E-03	2.841	3.71E-02	−2.534	phosphodiesterase 1C, calmodulin-dependent 70kDa	Cytoplasm	enzyme
PDE4DIP	1.74E-03	1.527	3.06E-03	3.065	phosphodiesterase 4D interacting protein	Cytoplasm	enzyme
PDIA2	4.18E-02	1.722	3.19E-03	3.164	protein disulfide isomerase family A, member 2	Cytoplasm	enzyme
PGA3	2.38E-02	2.179	3.35E-02	1.99	pepsinogen 3, group I (pepsinogen A)	Extracellular Space	peptidase
PKIA	1.81E-04	2.597	4.94E-02	2.302	protein kinase (cAMP-dependent, catalytic) inhibitor alpha	Cytoplasm	other
PKIB	2.77E-03	−2.292	4.55E-05	4.903	protein kinase (cAMP-dependent, catalytic) inhibitor	unknown	other

					beta		
PLAU	8.21E-07	−1.92	2.16E-03	2.028	plasminogen activator, urokinase	Extracellular Space	peptidase
PLXNC1	4.80E-02	−1.887	1.44E-02	2.593	plexin C1	Plasma Membrane	other
PPARA	2.17E-04	2.282	3.39E-02	2.001	peroxisome proliferator-activated receptor alpha	Nucleus	ligand-dependent nuclear receptor
PRICKLE1	2.22E-03	1.596	1.21E-03	−1.799	prickle homolog 1 (Drosophila)	Nucleus	other
PRKAA2	4.62E-02	1.982	2.28E-02	−2.406	protein kinase, AMP-activated, alpha 2 catalytic subunit	Cytoplasm	kinase
PTPN12	1.28E-02	−3.212	1.18E-03	1.822	protein tyrosine phosphatase, non-receptor type 12	Cytoplasm	phosphatase
PTPRD	4.98E-02	2.828	1.90E-02	3.673	protein tyrosine phosphatase, receptor type, D	Plasma Membrane	phosphatase
PYCR2	7.91E-04	1.678	3.82E-04	1.922	pyrroline-5-carboxylate reductase family, member 2	Cytoplasm	enzyme
RAD54B	1.66E-03	3.391	4.61E-02	1.574	RAD54 homolog B (S. cerevisiae)	Nucleus	enzyme
RAP2B	1.86E-04	−1.529	9.16E-05	3.796	RAP2B, member of RAS oncogene family	Plasma Membrane	enzyme
RECQL	1.39E-06	2.077	1.33E-07	3.08	RecQ protein-like (DNA helicase Q1-like)	Nucleus	enzyme
RETNLB	5.57E-06	−2.492	2.49E-05	1.927	resistin like beta	Extracellular Space	other
RGS16	6.43E-05	2.693	2.63E-04	2.095	regulator of G-protein signaling 16	Cytoplasm	other
RGS2	4.33E-06	3.905	5.12E-04	1.688	regulator of G-protein signaling 2, 24kDa	Nucleus	other
RIF1	5.86E-03	2.312	8.33E-03	−2.141	RAP1 interacting factor homolog (yeast)	Nucleus	other
RPAIN	2.02E-06	1.526	7.06E-04	2.379	RPA interacting protein	Nucleus	other
RTN4IP1	1.14E-02	1.591	9.62E-04	2.655	reticulon 4	Cytoplasm	enzyme

					interacting protein 1		
SFT2D3	1.18E-02	−1.776	1.88E-02	−1.59	SFT2 domain containing 3	unknown	other
SHISA9	2.19E-02	1.959	2.26E-02	−1.944	shisa homolog 9 (Xenopus laevis)	Plasma Membrane	other
SLAMF7	6.23E-03	1.857	2.85E-03	1.603	SLAM family member 7	Plasma Membrane	other
SLC13A3	7.21E-03	−2.433	2.69E-02	−1.776	solute carrier family 13 (sodium-dependent dicarboxylate transporter), member 3	Plasma Membrane	transporter
SLC30A4	3.76E-02	2.535	2.31E-02	1.941	solute carrier family 30 (zinc transporter), member 4	Cytoplasm	transporter
SLC39A12	1.66E-02	1.795	7.23E-03	2.174	solute carrier family 39 (zinc transporter), member 12	unknown	transporter
SLC7A2	3.82E-02	2.707	2.43E-02	3.058	solute carrier family 7 (cationic amino acid transporter, y+ system), member 2	Plasma Membrane	transporter
SLC9A5	9.55E-03	2.432	1.77E-02	2.103	solute carrier family 9 (sodium/hydrogen exchanger), member 5	Plasma Membrane	transporter
SMAD3	9.16E-04	−1.631	2.45E-02	1.832	SMAD family member 3	Nucleus	transcription regulator
SOX6	2.38E-03	1.923	7.11E-04	1.831	SRY (sex determining region Y)-box 6	Nucleus	transcription regulator
SP110	1.59E-02	2.22	3.72E-02	1.78	SP110 nuclear body protein	Nucleus	other
SPIB	4.95E-03	−2.611	3.56E-02	−1.629	Spi-B transcription factor (Spi-1/PU.1 related)	Nucleus	transcription regulator
ST8SIA4	6.19E-03	−2.922	1.04E-03	−4.191	ST8 alpha-N-acetyl-neuraminide alpha-2,8-sialyltransferase 4	Cytoplasm	enzyme
STK32A	1.54E-02	1.624	8.99E-03	2.843	serine/threonine	unknown	kinase

					kinase 32A		
TADA2A	3.24E-03	1.64	1.21E-02	1.651	transcriptional adaptor 2A	Nucleus	transcription regulator
TANC2	1.57E-04	3.404	4.68E-02	1.973	tetratricopeptide repeat, ankyrin repeat and coiled-coil containing 2	Nucleus	transcription regulator
TBL1X	4.26E-02	−1.915	3.82E-02	1.568	transducin (beta)-like 1X-linked	Nucleus	transcription regulator
TBX22	8.65E-03	−2.666	1.41E-02	2.38	T-box 22	Nucleus	transcription regulator
TCTE3	4.28E-02	3.288	7.79E-03	2.92	t-complex-associated-testis-expressed 3	Plasma Membrane	other
TGM4	4.53E-02	1.617	1.28E-02	2.248	transglutaminase 4 (prostate)	Extracellular Space	enzyme
TIPIN	1.85E-03	2.078	3.51E-03	1.826	TIMELESS interacting protein	Nucleus	other
TJP3	1.54E-02	−2.177	6.20E-03	−2.678	tight junction protein 3 (zona occludens 3)	Plasma Membrane	other
TLN2	7.86E-04	1.876	2.43E-02	1.705	talin 2	Nucleus	other
TMEFF2	4.86E-04	3.23	1.22E-03	−1.702	transmembrane protein with EGF-like and two follistatin-like domains 2	Plasma Membrane	other
TNC	1.23E-04	2.749	1.71E-03	3.149	tenascin C	Extracellular Space	other
TPM1	1.39E-03	1.702	3.04E-02	1.77	tropomyosin 1 (alpha)	Cytoplasm	other
TRA2A	3.01E-02	−2.052	1.91E-03	2.748	transformer 2 alpha homolog (Drosophila)	Nucleus	other
TRA2B	2.12E-03	1.933	8.89E-03	2.865	transformer 2 beta homolog (Drosophila)	Nucleus	other
TRAFD1	9.79E-03	1.82	7.25E-04	−3.095	TRAF-type zinc finger domain containing 1	unknown	other
TSHR	1.16E-02	−2.456	4.37E-02	1.746	thyroid stimulating hormone receptor	Plasma Membrane	G-protein coupled receptor
UBE2T	9.96E-05	1.513	3.55E-06	2.692	ubiquitin-conjugating enzyme E2T (putative)	Nucleus	enzyme

UBR3	4.43E-02	−1.813	1.63E-02	−2.363	ubiquitin protein ligase E3 component n-recognin 3 (putative)	unknown	enzyme
USP34	8.21E-03	2.85	1.44E-02	2.505	ubiquitin specific peptidase 34	unknown	peptidase
VGLL3	4.63E-06	−3.209	3.66E-03	4.047	vestigial like 3 (Drosophila)	unknown	other
WNT3	1.58E-04	2.5	3.45E-02	2.653	wingless-type MMTV integration site family, member 3	Extracellular Space	other
WRNIP1	9.96E-03	2.403	4.91E-02	1.593	Werner helicase interacting protein 1	Nucleus	enzyme
XPNPEP3	1.66E-02	2.347	7.42E-03	1.897	X-prolyl aminopeptidase (aminopeptidase P) 3, putative	Cytoplasm	peptidase
YOD1	4.24E-02	−1.534	2.87E-02	−1.71	YOD1 OTU deubiquinating enzyme 1 homolog (S. cerevisiae)	unknown	enzyme
ZBTB32	7.50E-04	−2.029	1.39E-03	−1.802	zinc finger and BTB domain containing 32	Nucleus	transcription regulator
ZNF10	1.09E-04	5.256	3.07E-02	1.678	zinc finger protein 10	Cytoplasm	other
ZNF226	1.37E-02	1.816	1.53E-03	−2.893	zinc finger protein 226	Nucleus	transcription regulator
ZNF627	3.02E-02	1.585	1.53E-02	1.885	zinc finger protein 627	unknown	other
ZNF678	1.55E-02	−2.935	1.26E-02	3.081	zinc finger protein 678	Nucleus	other
ZNF77	1.53E-02	2.728	3.22E-03	2.85	zinc finger protein 77	Nucleus	other
ZNRD1-AS1	5.48E-03	1.565	1.01E-03	4.199	ZNRD1 antisense RNA 1 (non-protein coding)	unknown	other
